# *Corynebacterium parakroppenstedtii* secretes a novel glycolipid to promote the development of granulomatous lobular mastitis

**DOI:** 10.1038/s41392-024-01984-0

**Published:** 2024-10-21

**Authors:** Ran Liu, Zixuan Luo, Chong Dai, Yuchen Wei, Shuqing Yan, Xinwen Kuang, Kuan Qi, Aisi Fu, Yinxin Li, Shuai Fu, Zhengning Ma, Wen Dai, Xiao Xiao, Qing Wu, Haokui Zhou, Yan Rao, Jingping Yuan, Ting Shi, Zixin Deng, Chuang Chen, Tiangang Liu

**Affiliations:** 1 Renmin Hospital of Wuhan University, Wuhan University, 430071 Wuhan, China; 2 State Key Laboratory of Microbial Metabolism, Joint International Research Laboratory of Metabolic & Developmental Sciences, and School of Life Sciences and Biotechnology, Shanghai Jiao Tong University, 200240 Shanghai, China; 3 CAS Key Laboratory of Quantitative Engineering Biology, Shenzhen Institute of Synthetic Biology, Shenzhen Institute of Advanced Technology, Chinese Academy of Sciences, 518055 Shenzhen, China; 4 Key Laboratory of Combinatorial Biosynthesis and Drug Discovery, Ministry of Education and School of Pharmaceutical Sciences, Wuhan University, 430072 Wuhan, China; 5 Department of Microbiology, The Chinese University of Hong Kong, Hong Kong, China; 6 Zhongnan Hospital of Wuhan University, Wuhan University, 430071 Wuhan, China; 7 Dgensee Co., Ltd, 430073 Wuhan, China; 8 Hesheng Tech, Co., Ltd, 430073 Wuhan, China; 9 Animal Biosafety Level III Laboratory at the Center for Animal Experiment, Wuhan University School of Medicine, Wuhan University, 430071 Wuhan, China; 10 TaiKang Center for Life and Medical Sciences, Wuhan University, 430072 Wuhan, China

**Keywords:** Infectious diseases, Microbiology, Infection

## Abstract

Granulomatous lobular mastitis (GLM) is a chronic idiopathic granulomatous mastitis of the mammary gland characterized by significant pain and a high propensity for recurrence, the incidence rate has gradually increased, and has become a serious breast disease that should not be ignored. GLM is highly suspected relative to microbial infections, especially those of *Corynebacterium* species; however, the mechanisms involved are unclear, and prevention and treatment are difficult. In this study, we demonstrated the pathogenicity of *Corynebacterium parakroppenstedtii* in GLM using Koch’s postulates. Based on the drug sensitization results of *C. parakroppenstedtii*, and utilizing a retrospective study in conjunction with a comprehensive literature review, we suggested an efficacious, targeted antibiotic treatment strategy for GLM. Subsequently, we identified the pathogenic factor as a new type of glycolipid (named corynekropbactins) secreted by *C. parakroppenstedtii*. Corynekropbactins may chelate iron, cause the death of mammary cells and other mammary -gland-colonizing bacteria, and increase the levels of inflammatory cytokines. We further analyzed the prevalence of *C. parakroppenstedtii* infection in patients with GLM. Finally, we suggested that the lipophilicity of *C. parakroppenstedtii* may be associated with its infection route and proposed a possible model for the development of GLM. This research holds significant implications for the clinical diagnosis and therapeutic management of GLM, offering new insights into targeted treatment approaches.

## Introduction

Granulomatous lobular mastitis (GLM) is the most common type of non-lactational inflammatory mastitis or non-puerperal mastitis. It is a type of chronic idiopathic granulomatous mastitis first reported by Kessler in 1972.^1^ Over the past 50 years, GLM has been considered a rare benign breast disease in Western countries. However, over the past few decades, an increasing number of cases of GLM have been reported. Between 1995 and 2014, approximately 1,400 cases were reported,^2^ while from 2013 to 2018, an estimated 3060 cases were reported.^3^ According to published papers, 17,257 cases have been reported in English language journals and 16,711 cases have been reported in Chinese language journals in the last 10 years (2014–2023). Therefore, GLM has become a common breast disease in the last 10 years and has received increasing attention from clinicians and the public. GLM may have a major impact on quality of life because of its common delayed course and relapse of symptoms, such as breast masses/abscesses, tenderness, and skin changes ^4^(Supplementary Fig. 1a). The recurrence rate of GLM is as high as 24.8%.^5^ Moreover, patients with GLM may have approximately twice the risk of developing breast cancer compared with those without GLM.^6^ Therefore, GLM is a serious breast disease, and its pathogenic mechanisms in patients with GLM need to be further elucidated.

The etiology of GLM remains uncertain, and clear and reasonable guidance for its treatment has not yet been developed, although GLM has been established as an individual disease for more 50 years. GLM is associated with several factors, such as autoimmunity, bacterial infections, psychiatric disorders, trauma, and hyperprolactinemia.^7^ Specifically, since GLM has been reported to coexist with erythema nodosum and/or arthritis ^8^ and Ayca et al. observed significant differences in lymphocyte subsets in GLM patients compared with healthy volunteers,^9^ GLM has been considered an underrecognized systemic autoimmune disease. In addition, several investigations have shown that GLM may be associated with psychiatric disorders^10^ and hyperprolactinemia, including drug-induced hyperprolactinemia^11^ and hyperprolactinemia caused by intracranial lesions.^12^ However, this is a very small subset of the GLM population, and the key mechanisms involved remain unclear. An association between GLM and region was observed because GLM cases have been widely reported in the Mediterranean region and developing Asian countries.^7^ Steroids, antibiotics, irrigation, and surgical excision are the treatment options;^7^ however, the efficacies of these treatments remain controversial. For example, Kazuhisa et al. concluded that steroid therapy may be the preferred treatment strategy for GLM because among the patients they reviewed, those who were treated with surgery or irrigation experience recurrence, while those treated with corticosteroids did not.^13^ However, a study showed that patients with GLM treated with steroids still endured recurrence and side effects occurred.^14^ Similarly, another study suggested that surgery may be the first choice for GLM treatment.^15^ Antibiotics also were chosen for treatment. However, Ercan et al.^16^ and Bhattarai et al.^17^ independently reviewed patients with GLM in two hospitals and concluded that empirical antibiotic therapy does not effectively increase cure rates. However, a study reported that extended courses of lipophilic antibiotics are a largely unexplored, but potentially effective, treatment option for GLM.^18^ Notably, Farouk et al. confirmed the effectiveness of rifampicin (a lipophilic antibiotic) administration as a stand-alone pharmacological treatment in GLM patients as an alternative to surgery and corticosteroids.^19^ Therefore, the pathogenic mechanisms underlying GLM must be elucidated to allow the development of precise treatments.

The association between GLM and microbial infections deserves further attention. Various microorganisms have been isolated from patients with GLM, including *Corynebacterium* species,^20^ *Mycobacterium abscessus*,^21^ *Staphylococcus* species,^22^ *Rhodococcus equi*,^23^ and *Gordonia terrae*.^24^ However, none of these microorganisms have been proven to cause GLM. In recent years, *Corynebacterium kroppenstedtii* has been frequently isolated from patients with GLM and detected by polymerase chain reaction (PCR) or sequencing.^25–29^ However, Luo et al. classified *C. kroppenstedtii*-like strains into three species, *C. kroppenstedtii* and two novel species (*C. parakroppenstedtii* and *C. pseudokroppenstedtii*).^30^ They investigated all *C. kroppenstedtii*-like isolates cultured from female patients with mastitis in their hospital, and the isolates belonged to the two new species. Therefore, *C. kroppenstedtii*-like strains isolated from patients with mastitis or GLM reported in previous studies may be *C. parakroppenstedtii* or *C. pseudokroppenstedtii*, rather than *C. kroppenstedtii*. Although *C. kroppenstedtii*-like bacteria are highly suspected to be associated with GLM, whether GLM is caused by infection is unknown, and the associated pathogenic mechanisms are unclear.

To understand the associations between GLM and microbial infection, we systematically explored the etiology and treatment of this disease and prevention strategies. First, we identified and confirmed the pathogenicity of *C. parakroppenstedtii* in GLM by Koch’s postulates. Then, we test the drug sensitization of *C. parakroppenstedtii* to explore possible effective treatment strategy for GLM. We then investigated the pathogenic mechanisms of *C. parakroppenstedtii*. Moreover, we investigated the prevalence of *C. parakroppenstedtii* in patients with GLM. Finally, we explored its possible infection route of *C. parakroppenstedtii* and proposed a possible model for the development of GLM.

## Results

### 16S rRNA gene sequencing to explore possible infectious microorganisms in patients with GLM

**Fig. 1:**
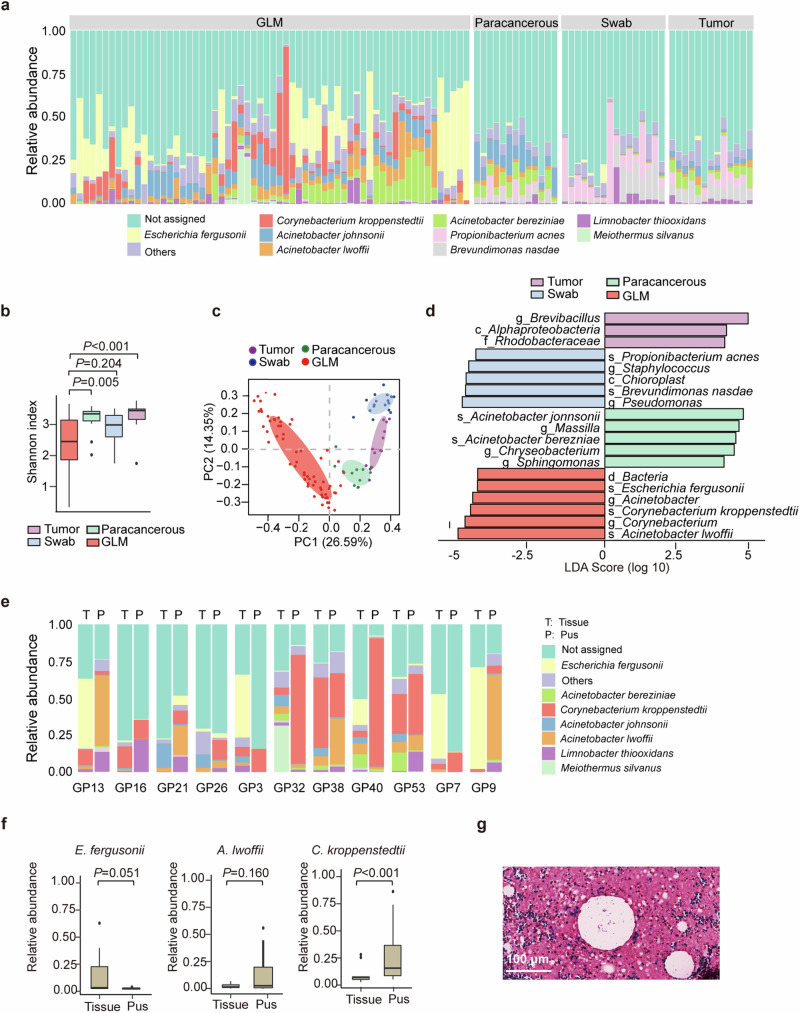
Exploration of possible causative pathogen of Granulomatous lobular mastitis (GLM). **a** Relative abundance of bacteria in different breast samples. **b** Comparison of the alpha-diversity and Shannon index values of the bacteria in different breast samples. Data are expressed as median + interquartile range (IQR), and statistical significance was calculated using the Wilcoxon rank sum test with the Bonferroni method for multiple testing correction. **c** Principal coordinate analysis of the microbiota based on Bray‒Curtis heterogeneity of bacteria from different breast samples. **d** Linear discriminant analysis effect size of the bacterial microbiota in different breast samples. g: genus, c: class, f: family, s: species, d: domain. **e** Relative abundance of bacteria in the pus and breast tissues of 11 patients with GLM. **f** The relative abundances of *Escherichia fergusonii, Corynebacterium kroppenstedtii*, and *Acinetobacter lwoffi* in the pus and breast tissues of 11 patients with GLM. Data are expressed as the median + IQR, and a paired Wilcoxon rank sum test was performed for comparison. **g** Gram staining results of tissue sections from patients with GLM

To identify possible pathogenic microbes in patients with GLM, we collected 62 residual breast tissue samples from patients with GLM (Supplementary Table 1), 13 pairs of residual breast tumor tissue and paracancerous tissue from patients with breast cancer, and 16 breast epidermal swab specimens from healthy volunteers for 16S rRNA gene sequencing. Figure 1a illustrates the species-level relative abundances of all samples, showing that *C. kroppenstedtii*, *Escherichia fergusonii*, *Acinetobacter lwoffi, A. johnsonii*, and *A. bereziniae* exhibited higher proportions in the GLM group. The alpha-diversity results showed that the GLM group had less microbial diversity than the other three groups, and significant differences in the Shannon index values were found between the GLM and tumor groups, and between the GLM and paracancerous tissue groups (Fig. 1b). However, no significant differences in the Shannon index values were observed between the GLM and swab groups. Bray-Curtis dissimilarity analysis among the four groups showed that the composition of the microbiota in the GLM samples differed significantly from that in the other three groups (Fig. 1c). Linear discriminant analysis effect size was used to identify specific taxa that could explain the differential microbial composition among the groups. The results showed that the GLM group was enriched in *C. kroppenstedtii* (the databases were unable to distinguish *C. kroppenstedtii*-like bacteria, which were uniformly identified as *C. kroppenstedtii*), *E. fergusonii*, and *A. lwoffi* at the species level (Fig. 1d), suggesting that these bacteria are suspected pathogenic bacteria.

The development of GLM usually begins with the development of redness and swelling in breast tissue, which gradually deteriorates to form pus as the disease progresses. Because pus is a product of the immune response to infection and some other infectious disease studies have confirmed that the abundance of pathogenic bacteria in pus is higher than that in tissue or other samples,^31,32^ we hypothesized that the abundance of pathogenic bacteria in pus was also higher than that in breast tissue in patients with GLM. Therefore, we collected breast tissue and pus samples from 11 patients with GLM and performed 16S rRNA gene sequencing (Supplementary Table 2) to compare the relative abundance of the three suspected pathogenic bacteria. The results showed that the relative abundance of *C. kroppenstedtii* was consistently higher in the pus samples than the tissue samples from eight of these 11 patients (Fig. 1e), among the three suspected bacteria, only *C. kroppenstedtii* had a significantly higher relative abundance in pus than in tissues from patients with GLM (Fig. 1f).

The sequencing approach does not discriminate between live, dead, and contaminating microorganisms during sample collection, preparation, and sequencing. Therefore, *C. kroppenstedtii*, *E. fergusonii*, and *A. lwoffi* may not be the actual microorganisms present in patients with GLM. Indeed, only *C. kroppenstedtii* was cultured in these samples, and the other two bacteria were not cultured in these samples (Supplementary Table 1). Moreover, *E. fergusonii* and *A. lwoffi* have not been reported to be cultured from samples of patients with GLM/mastitis, whereas *C. kroppenstedtii* has been reported to be the dominant strain in patients with GLM at hospitals in different regions.^25,33,34^ Therefore, it is likely that *E. fergusonii* and *A. lwoffi* are not present in clinical GLM samples, whereas *C. kroppenstedtii* is widely distributed in patients with GLM. Since *C. kroppenstedtii* is a gram-positive bacterium, we subsequently selected pathological tissues from patients with GLM for Gram staining, and gram-positive bacteria were observed in tissue sections from several patients (Fig. 1g), which further deepened the suspicion of *C. kroppenstedtii*. Of the 62 GLM samples collected in our study, *C. kroppenstedtii* was detected in 50 (Supplementary Table 1), indicating that *C. kroppenstedtii*-infection was highly correlated with the GLM.

### Koch’s postulates verify the pathogenicity of *C. parakroppenstedtii* to GLM

**Fig. 2:**
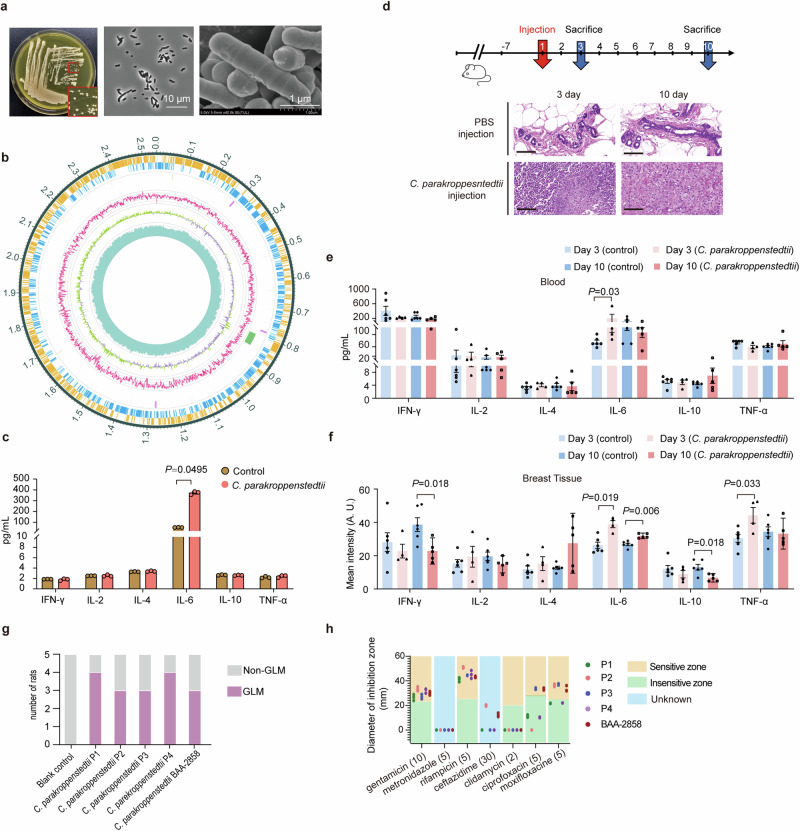
*C. parakroppenstedtii* causes GLM in rats. **a** Images of *C. parakroppenstedtii*. Left: typical colonies of *C. parakroppenstedtii* in BHIY with Tween 80. Middle: microscopic view of *C. parakroppenstedtii*. Right: *C. parakroppenstedtii* observed by scanning electron microscopy. **b** Genome circle map of *C. parakroppenstedtii* P1. From outside to inside, coding genes (positive-sense strand), coding genes (negative-sense strand), tRNA (orange) and rRNA (purple), gene islands (green), GC ratio, GC skew, and sequencing depth. **c** *C. parakroppenstedtii* P1 causes changes in cytokine levels in MCF-10A cells. Three biological replicates were set up for each group. Data are presented as the mean values ± standard deviation (SD), and Kruskal–Wallis tests were performed to calculate statistical significance. **d** Verification of the occurrence of GLM after *C. parakroppenstedtii* P1 infection. The experiment consisted of a control (phosphate-buffered saline [PBS]) group (12 rats) and a *C. parakroppenstedtii* P1 group (12 rats). After breast fat pad injection, six rats in each group were sacrificed on day 3 and day 10. Pathologic results are typical representative images selected from the supplementary fig. 2. Scale bar represents 100 μm. **e** The serum cytokine levels in the rats were determined using a Luminex assay. Data were compared between the rats with obvious inflammation (day 3: *n* = 4; day 10*: n* = 5) and those in the control group (*n* = 6). Data are presented as the mean values ± standard error of the mean (SEM), and Kruskal–Wallis tests were performed to calculate statistical significance. **f** The levels of cytokines in the breast tissue of rats were determined by immunofluorescence staining. Data were compared between the rats with obvious inflammation (day 3: *n* = 4; day 10: *n* = 5) and those in the control group (*n* = 6). Data are presented as the mean values ± SEM, and Kruskal–Wallis tests were performed to calculate statistical significance. **g** Verification of the occurrence of GLM after infection with different *C. parakroppenstedtii* strains or the blank control (PBS). There were five rats in each group. The results were observed after sacrifice on day 10 after breast fat pad injection. **h** Antimicrobial susceptibility testing of *C. parakroppenstedtii* with different antibiotics performed via disk diffusion (Becton Dickinson Deutschland, Heidelberg, Germany) as suggested for *Corynebacterium* by the European Committee on Antimicrobial Susceptibility Testing (http://www.eucast.org)

To verify whether the *C. kroppenstedtii*-like strain is a pathogenic bacterium of GLM, we first isolated a strain from the pus of a patient with GLM (Fig. 2a) using brain–heart infusion broth with Tween 80, which is suitable for the growth of *C. kroppenstedtii*.^35^ We sequenced the strain using next-generation sequencing (NGS) and Nanopore combined sequencing to obtain a complete genome map of approximately 2.5 Mb (Fig. 2b). Using bioinformatic analysis, this strain was classified as *C. parakroppenstedtii* and named *C. parakroppenstedtii* P1. Microscopic observations of *C. parakroppenstedtii* P1 showed that some bacteria adhered to each other and were arranged in clusters (Fig. 2a). The scanning electron microscopy results showed multiple ring-shaped bands on the surface of the bacteria (Fig. 2a).

Clinical diagnoses of GLM were based on histological investigations using hematoxylin and eosin (H&E) staining (Supplementary Fig. 1b). However, because one of the clinical difficulties in the diagnosis of GLM is the search for diagnostic markers, besides the gold standard for GLM diagnosis, we further tested six cytokines that are commonly detected clinically to search as possible clinical markers. Therefore, we reviewed patients with GLM at our hospital who underwent serum cytokine Th1/Th2 level testing (*n* = 13). Among the six tested cytokines, the levels of IL-2, IL-4, IL-10, IFN-γ, and TNF-α were in the normal range in all of the patients, while upregulation of the inflammatory factor IL-6 was observed in 54% of the patients (Supplementary Table 3), which is consistent with the findings of a previous study.^36^ Next, we selected six pathological GLM breast tissues and 12 breast samples that were similar to normal breast tissues (six breast fibroadenoma tissues and six paracancerous tissues) (Supplementary Table 4) to perform immunofluorescence staining of cytokines. The levels of IL-6 and TNF-α were significantly higher in GLM tissue than in fibroadenoma tissue and paracancerous tissue, but there was no significant difference in the levels IL-2, IL-4, IL-10, or IFN-γ among the groups (Supplementary Fig. 1c). These results suggest that the typical clinical characteristics of patients with GLM include typical GLM-related pathological histomorphology, the upregulation of serum IL-6 levels, and the upregulation of IL-6 and TNF-α levels in breast tissue.

To verify whether *C. parakroppenstedtii* P1 can cause GLM, we cultured *C. parakroppenstedtii* P1 with human mammary epithelial MCF-10A cells for 24 h, and cell supernatants were collected for Th1/Th2 cytokine assays. The results showed significant IL-6 upregulation compared to the control group, whereas the levels of the other five cytokines were not significantly different from those in the control group (Fig. 2c). We then injected *C. parakroppenstedtii* P1 (experimental group) or phosphate-buffered saline (PBS; control group) into the mammary fat pads of rats and observed pathological changes in the mammary glands and changes in cytokine levels in the blood and mammary glands on days 3 and 10, respectively (Fig. 2d). H&E staining revealed typical pathological morphology of granulomatous foci, tissue necrosis, and multinucleated giant cells in the experimental group (Fig. 2d). GLM developed in all rats in the experimental group (Supplementary Fig. 2); among them, two rats on day 3 and one rat on day 10 had a smaller area of inflammation than other rats in the experimental group (Supplementary Fig. 3). The presence of *C. parakroppenstedtii* in the infected mammary tissue was confirmed after culturing individual colonies from pathological tissue and performing Sanger sequencing of the 16S rRNA gene and Nanopore targeted sequencing (NTS) directly on the pathological tissue.

Next, we measured the cytokine levels in the serum and breast tissues of rats. The experimental group with apparent inflammation was compared with the corresponding control group. Only serum IL-6 levels were significantly upregulated in the experimental group compared to the control group in the rats sacrificed on day 3 (Fig. 2e). In the breast tissue, IL-6 and TNF-α levels were significantly higher in the experimental group than the control group in rats sacrificed on day 3, and IL-6 levels were significantly higher in the experimental group than the control group in rats sacrificed on day 10 (Fig. 2f). The upregulation of inflammatory factors on day 3 in rats with GLM was consistent with the results observed in most patients with GLM. This is probably because most of serum and biopsy samples from patients with GLM used for cytokine assays are collected at the time of the initial diagnosis, when most of them are in the relatively early stage of GLM. These results confirmed that *C. parakroppenstedtii* P1 causes GLM in rats, and suggested that *C. parakroppenstedtii* P1 could cause GLM in humans.

Next, we isolated three *C. kroppenstedtii*-like strains from breast pus of three patients with GLM. We acquired one strain (BAA-2858) from the American Type Culture Collection that was isolated from vaginal swabs to distinguish it from the strain isolated from breast pus. These strains were subjected to NGS and Nanopore sequencing to obtain their complete genomes. Bioinformatics analysis showed genomic similarity of 97.4–99.9% among the five strains. The strains were identified as *C. parakroppenstedtii* and named *C. parakroppenstedtii* P2, *C. parakroppenstedtii* P3, *C. parakroppenstedtii* P4, and *C. parakroppenstedtii* BAA-2858. These five strains were used in rat mammary gland infection experiments (Fig. 2g and Supplementary Fig. 4) resulting in 60%-80% of the rats in each group developing GLM, indicating that all five strains could cause GLM. However, some rats did not have obvious GLM symptoms, and we speculate that these rats may have had self-healing abilities, which is consistent with the clinical observation that some patients with GLM improve without treatment. These results suggested that all *C. parakroppenstedtii* strains may cause GLM.

As we confirmed that *C. parakroppenstedtii* is the pathogenic bacterium of GLM, we tested the susceptibility of *C. parakroppenstedtii* to several antibiotics (Fig. 2h). All five strains of *C. parakroppenstedtii* were sensitive to gentamicin and rifampicin, but not to metronidazole or clindamycin. Ciprofloxacin and moxifloxacin showed different efficacies among the strains. Three strains had no zone of inhibition with ceftazidime, and because there were no criteria for judging the results, we were unable to determine whether the other two strains were sensitive. Overall, the results showed that *C. parakroppenstedtii* is a multidrug-resistant strain, for which common empirical antibiotic treatments are less effective; however, the antimycobacterial drug rifampicin was more effective. We reviewed the cases and conducted telephone interviews with 16 patients with GLM who tested positive for *C. kroppenstedtii* at our center between June 2017 and May 2019 to calculate their recurrence rates. Based on the results of drug susceptibility to *C. parakroppenstedtii*, we divided the patients into a sensitive group (management protocols including gentamicin or rifampicin) and an insensitive group (management protocols without gentamicin or rifampicin). Until the time of our investigation (August 2022), all patients (Supplementary Table 5) in the sensitive group had no recurrent GLM, while 4 of the 6 patients in the insensitive group had recurrent GLM within 1.5 to 2 years after the initial cure. Moreover, previous studies concluded that empirical antibiotic therapy is ineffective at improving the cure rate of GLM,^16,17^ while a prospective study by Farouk et al. confirmed the effectiveness of rifampicin in the treatment of GLM,^19^ and another prospective study by Zhou et al. showed that 79% (65/82) of patients with GLM were cured with rifampicin-based triple therapy alone.^37^ In addition, Li et al. retrospectively investigated the efficiency of rifampicin for the treatment of 107 patients with GLM who tested positive for *C. kroppenstedtii* (current clinical microbiology test methods do not distinguish *C. kroppenstedtii*-like strains, all of which were recognized as *C. kroppenstedtii)*.^33^ The recurrence rate was significantly lower in the group in which management protocols included rifampicin than in those not taking rifampicin. Therefore, management protocols including antibiotics to which *C. parakroppenstedtii* is sensitive are efficient treatment protocols for patients with GLM.

### One of the pathogenic factors in *C. parakroppenstedtii* is a glycolipid

**Fig. 3:**
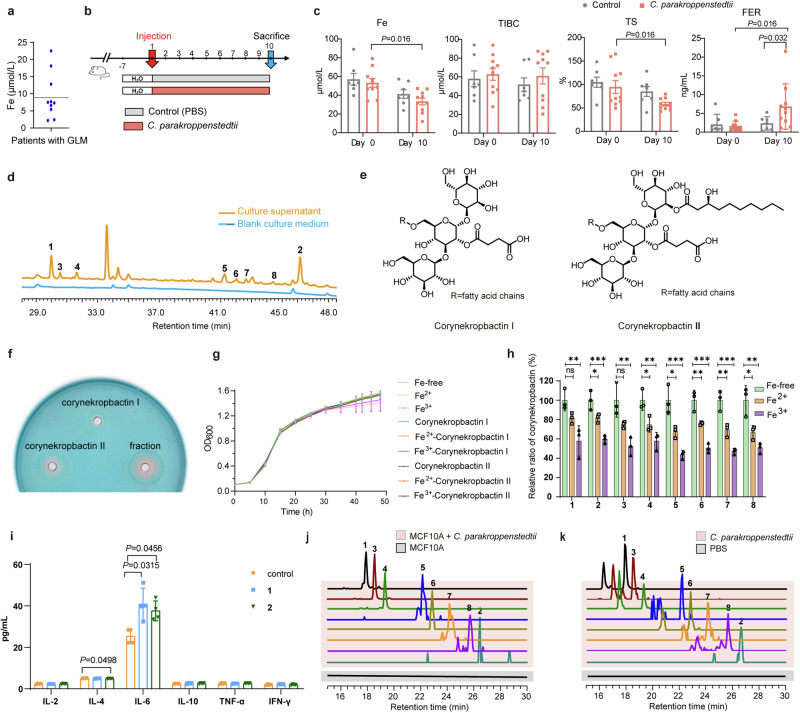
Novel glycolipids generated by *C. parakroppenstedtii* and their roles. **a** Concentration of serum iron in 10 patients with GLM who had a confirmed diagnosis of *C. kroppenstedtii* infection. **b** Verification of iron deficiency in rats infected with *C. parakroppenstedtii* P1. The experiment consisted of a control (PBS) group (7 rats) and a *C. parakroppenstedtii* P1 group (10 rats). After breast fat pad injection, rats in each group were sacrificed on day 10. **c** Iron-related indicators in the serum of rats with GLM. Fe: iron ions; TIBC: total iron binding capacity; TS: transferrin saturation; FER: ferritin. Data are presented as the mean values ± SEM, and group comparisons were calculated using Kruskal–Wallis test or Wilconxon singed rank exact test (*P* value was adjusted using Benjamini-Hochberg correction) as appropriate. **d** High-performance liquid cohrmatography analysis of culture supernatants from *C. parakroppenstedtii* fermentation. **e** Structures of corynekropbactins from *C. parakroppenstedtii*. **f** Compounds on CAS detection media, corynekropbactin I: 0.8 mg; corynekropbactin II: 0.8 mg; fraction: 50 µL fraction containing corynekropbactins during purification. **g** Growth curves of *C. parakroppenstedtii* P1 in different PGT culture media with Tween 80. Except for the Fe-free group, the corresponding compounds of the remaining groups were added at 10 µM. Three biological replicates were set up for each group. Data are presented as the mean values ± SD. **h** The relative ratio of corynekropbactins in different PGT culture media with soy oil. The relative ratio was calculated as the ratio of the production of corynekropbactins in each group to that in the Fe-free group. Fe^2+^ and Fe^3+^ were added at 10 µM. Three biological replicates were set up for each group. Data are presented as the mean values ± SD, and ordinary one-way analysis of variance with Bonferroni correction for multiple comparison was performed to calculate statistical significance. *P < 0.05; **P < 0.01; ***P < 0.001. **i** Compounds **1** and **2** caused changes in the levels of cytokines in MCF-10A cells. Four biological replicates were set up for each group. Data are presented as the mean values ± SD, and Dunn’s test with Benjamini–Hochberg correction for multiple comparisons was performed to calculate statistical significance. **j** Liquid chromatography (LC)-tandem mass spectrometry (MS/MS) extracted ion chromatograms of supernatants of MCF-10A or *C. parakroppenstedtii*-treated MCF-10A cells. m/z of extracted ions: **1-**811.3582, **2-**1037.5491, **3-**837.3735, **4-**813.3735, **5-**979.4710, **6-**1007.5027, **7-**955.4711, **8**-983.5058. (**k**) LC-MS/MS extracted ion chromatogram of PBS-injected or *C. parakroppenstedtii*-injected rats. m/z of extracted ions: **1-**811.3582, **2-**1037.5491, **3-**837.3735, **4-**813.3735, **5-**979.4710, **6-**1007.5027, **7-**955.4711, **8**-983.5058. The experiment consisted of a control (PBS) group (6 rats) and a *C. parakroppenstedtii* P1 group (6 rats). Breast fad pad injections were performed on day 1 and day 4, and the rats in each group were sacrificed on day 8

We confirmed that *C. parakroppenstedtii* can cause GLM, but no obvious pathogenic factors were annotated from the genomic information. Therefore, we investigated the characteristics of the patients for clues and observed that 10 of them with confirmed *C. kroppenstedtii*-like strain infection had serum iron levels tested, seven of whom showed serum iron levels below the normal range (Fig. 3a, Supplementary Table 6). This suggested that iron deficiency may occur in patients with GLM.

Subsequently, we investigated whether iron deficiency was caused by *C. parakroppenstedtii* infection. We measured iron-related indicators in the sera of rats infected with *C. parakroppenstedtii* P1 (experimental group) or PBS (control group) (Fig. 3b). Ten days after injection, the ferritin concentration was significantly higher in the *C. parakroppenstedtii* group than the control group (Fig. 3c). Moreover, in the *C. parakroppenstedtii* group, the concentrations of serum iron and transferrin saturation were significantly lower after *C. parakroppenstedtii* infection than before *C. parakroppenstedtii* infection, and the concentration of ferritin was significantly higher than before *C. parakroppenstedtii* infection (Fig. 3c). These results suggested that *C. parakroppenstedtii* infection causes iron deficiency in rats.

Siderophores are low molecular weight, high-affinity iron chelators produced by bacteria. Bacteria utilize siderophores to take in iron from the host organism for growth and metabolism during infection; therefore, siderophores are crucial pathogenic factors.^38^ Given that *C. parakroppenstedtii* infection causes iron deficiency in rats, we suspected that *C. parakroppenstedtii* generates siderophores. Therefore, we attempted to isolate siderophores from culture supernatants of *C. parakroppenstedtii*. Using the classical chrome azurol S (CAS) reagent as an indicator,^39^ several compounds (**1–8**) absent in the blank culture medium (Fig. 3d) were isolated from the culture supernatant and named corynekropbactins. The structures of these compounds were resolved using a combination of spectroscopic and chemical methods (Fig. 3e, Supplemental Results). Corynekropbactins consist of an oligosaccharide core that is defined as α-D-glucopyranoside, α-D-glucopyranosyl-O-β-D-glucopyranosyl-(1 → 3) and at least two aglycones that contain succinic acid and fatty acids. Succinic acid (Agly′) is fixed to C-Glu″-2 of the oligosaccharide core and a fatty acid chain is connected to C-Glu″-6. The corynekropbactins were categorized into type I (with H) and type II (with 3-hydroxycapric acid) based on the group connected to the C-Glu′-2 of the oligosaccharide core (Supplementary Fig. 5). Corynekropbactins are structurally defined glycolipids. The classical glycolipids, trehalose-6′,6′-dimycolate and trehalose-6′-mycolate, identified in *Mycobacterium tuberculosis*,^40^ and trehalose dicorynomycolate and trehalose monocorynomycolate, identified in *Corynebacterium* species,^41^ with a structure consisting of a trehalose and long-chain fatty acids (C > 20), are also crucial classes of pathogenic factors. The corynekropbactins identified in this study have a structure that differs from previously reported structures in terms of the glycosyl group composition and an extra succinic acid.

However, the corynekropbactins we identified have a structure that is inconsistent with the four classic structures that are currently considered siderophores.^42^ To test whether corynekropbactins can chelate iron ions, we first tested the colorimetric responses of corynekropbactins I, II, and a fraction containing corynekropbactins during purification using a CAS plate. The results showed that the fractions and corynekropbactin II had obvious orange circles (Fig. 3f). We chose **1** and **2** to represent corynekrobactin I and II, respectively, to react with the CAS solution. When 40 µM of **1** and **2** reacted with CAS, the absorbance of the reaction solution of **1** at 630 nm decreased from 0.3263 to 0.3216 and that of **2** from 0.3263 to 0.2693. Next, to test the specificity of the chelating ability of the corynekropbactins, six metal ions (Fe^2+^, Fe^3+^, Cu^2+^, Mn^2+^, Co^2+^, and Ni^2+^) were reacted with **1** and **8**. The results showed no apparent difference in the λmax values of the corynekropbactins and their metal complexes (Supplementary Fig. 6), which may be because corynekropbactins appear to have only terminal absorption in the UV spectra and no distinct characteristic absorption peaks in the 300–1,000 nm range. However, the absorbances of the **Fe**^2+^**-complex** and **Fe**^3+^**-complex** differed from those of **1** and **8**. To confirm whether corynekropbactins react chemically with Fe^2+^ and Fe^3+^, we performed nuclear magnetic resonance (NMR) analysis of **8** and its **Fe**^2+^ **-complex** and **Fe**^3+^**-complex**. A comparison of the ^13^C NMR data of **8** with those of **Fe**^2+^**-complex** and **Fe**^3+^**-complex** showed that the resonances of C-Glu′-2 were shifted upfield by Δ*δ*_C_ 0.2 ppm, and the signals of C-Agly′-1 were significantly weakened (Supplementary Fig. 7), suggesting that **8** may chelate both Fe^2+^ and Fe^3+^ and that the carbonyl oxygen at the C-Agly′-1 and the hydroxyl group at the C-Glu′-2 are both binding sites for the chelation reaction of **8**.

To test whether the corynekropbactins are siderophores of *C. parakroppenstedtii*, we examined the effects of corynekropbactins and their ferric complexes on the growth of *C. parakroppenstedtii*. The growth rate of *C. parakroppenstedtii* in PGT medium, with or without iron ions, corynekropbactins, and ferric corynekropbactins, showed no apparent differences (Fig. 3g), indicating that *C. parakroppenstedtii* does not depend on iron ions for growth. However, we tested whether iron ions affected the production of corynekropbactins. Fe^2+^ and Fe^3+^ significantly inhibited corynekroptobactin production (Fig. 3h). Therefore, corynekropbactins may chelate iron, and their production is correlated with the extracellular iron content; however, the growth of *C. parakroppenstedtii* does not seem to depend on iron. Therefore, corynekropbactins do not satisfy all the characteristics of a siderophore, and their physiological function in *C. parakroppenstedtii* remains to be determined.

Subsequently, we explored whether corynekropbactins produced by *C. parakroppenstedtii* play a role in the pathogenesis of this bacterium. We co-cultured **1** and **2** with MCF-10A cells and measured their growth status after 48 h. These compounds inhibited the growth of mammary cells, with **2** (50% inhibitory concentration [IC_50_] = 52.667 μM) being more efficient than **1 (**IC_50_ = 300.388 μM**)**. Next, we co-cultured MCF-10A cells with **1** and **2** at their IC_50_ for 6 h and measured changes in cytokine levels. IL-6 was significantly upregulated in the supernatants of cells treated with **1** and **2** compared to the supernatants of control cells, but there were almost no changes in the levels of the other five cytokines, except for a slight downregulation of IL-4 after treatment with **2** (Fig. 3i). Next, we prepared equal amounts of live *C. parakroppenstedtii* and heat-killed *C. parakroppenstedtii*. Liquid chromatography-mass spectrometry (LC-MS) detection and quantification revealed no difference in corynekropbactins production between the two groups (Supplementary Fig. 8a). After co-culturing MCF-10A cells with live and heat-killed *C. parakroppenstedtii* for 12 h, both groups of samples showed upregulated IL-6 levels, with the live group showing significantly higher levels than the heat-killed group (Supplementary Fig. 8b), indicating the presence of pathogenic factors other than corynekropbactins in *C. parakroppenstedtii*. This suggested that corynekropbactins may be one of pathogenic factors in *C. parakroppenstedtii*.

Moreover, we tested bacterial inhibition by compounds **1** and **2** (0.01 μmol) using agar diffusion assays. Compound **2** inhibited *Lactococcus lactis* J1-004, *L. lactis* MG1363, and *C. glutamicum* ATCC13032, but not *Lactobacillus reuteri* DSM17938 or the gram-negative bacterium *Escherichia coli* DH10b (Supplementary Table 7). Furthermore, we confirmed the inhibitory ability of compound **2** against *L. lactis* J1-004 (minimum inhibitory concentration [MIC] = 400 μM), *L. lactis* MG1363 (MIC = 200 μM), and *C. glutamicum* ATCC13032 (MIC = 12 μM).

Next, we verified whether corynekropbactins were generated during the interaction between *C. parakroppenstedtii* and breast tissue. We co-cultured MCF-10A cells with *C. parakroppenstedtii*, and the results (Fig. 3j) showed that **1–8** could be detected in the cell culture supernatant after 72 h, and the concentrations of **1,** **3**, and **4** were sufficient for MS/MS confirmation (Supplementary Fig. 9a). We then injected rat breasts with PBS (control group) or *C. parakroppenstedtii* (experimental group). Compounds **1–8** could be detected in the experimental group (Fig. 3k), and the concentrations of **1,** **3,** **4,** **6,** **7**, and **8** were sufficient for MS/MS confirmation (Supplementary Fig. 9b). These results confirmed that *C. parakroppenstedtii* can generate corynekropbactins during interaction with the breast.

Together, these results implied that the corynekropbactins produced by *C. parakroppenstedtii* may consume iron, kill mammary cells, and stimulate the upregulation of the host IL-6 to cause GLM. In addition, **2** can kill common colonizing bacteria of the breast (e.g., *L. lactis*) and some gram-positive bacteria (e.g., *C. glutamicum*), thus establishing their colonization status.

### The prevalence of *C. parakroppenstedtii* infection in patients with GLM

**Fig. 4:**
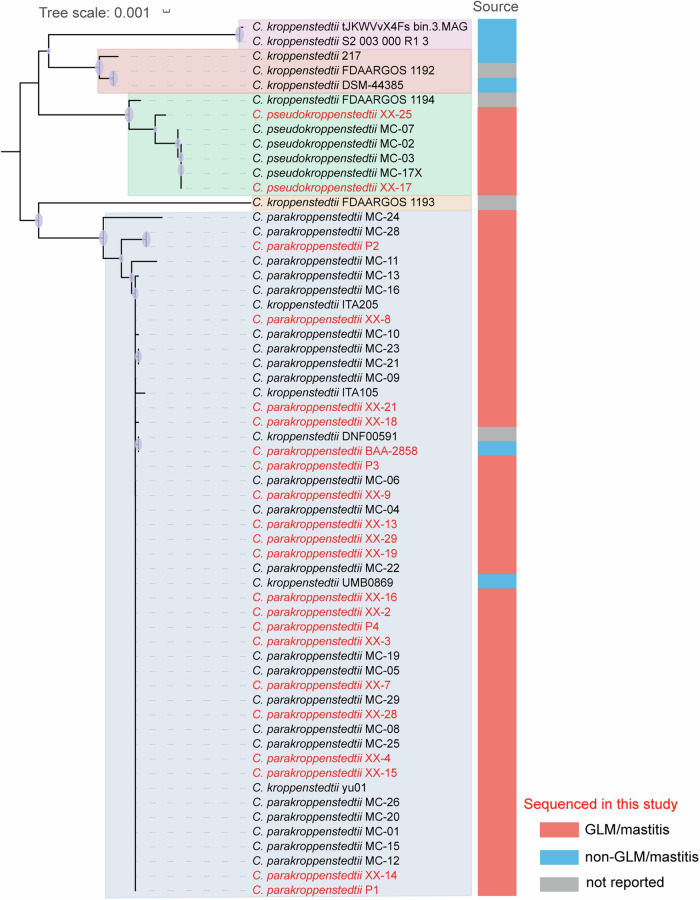
Phylogenetic trees of *C. kroppenstedtii*-like strains. The phylogenetic tree was constructed using whole-genome sequences, the strains marked in red were whole-genome sequenced in this study, while the whole-genome sequences of the strains marked in black were obtained from the NCBI database. Five major clusters with different background colors indicate that the *C. kroppenstedtii*-like strains can be divided into five species. The strains sequenced in this study were isolated from patients with GLM, and the sources of the remaining strains were defined according to their reported genome databases

To investigate whether *C. parakroppenstedtii* is the major infectious *C. kroppenstedtii*-like bacterium in GLM, we performed NGS and Nanopore combined whole-genome sequencing of 17 other *C. kroppenstedtii*-like bacteria isolated from clinical specimens of patients with mastitis at Zhongnan Hospital, Wuhan, China. We also searched all uploaded genomes of *C. kroppenstedtii*-like strains in the NCBI database until 2022 (Supplementary Table 8). Phylogenetic tree analysis showed that these strains could be divided into five species, rather than the three previously reported species,^30^ with minor evolutionary differences (Fig. 4). In the *C. parakroppenstedtii* group, 44/47 strains were isolated from patients with GLM or mastitis, two were isolated from female urinary microbiota (UMB0869) or vaginal swabs (BAA-2858), and one did not have the source reported. In the *C. pseudokroppenstedtii* group, 6/7 strains were isolated from patients with GLM or mastitis, and the source of the other strain was not reported. Six other strains comprised three species, including *C. kroppenstedtii*, but none were isolated from patients with GLM or mastitis. Among the strains isolated from patients with GLM or mastitis, 88% (44/50) were *C. parakroppenstedtii* and 12% (6/50) were *C. pseudokroppenstedtii*, illustrating the prevalence of *C. parakroppenstedtii* infection in patients with GLM from different regions.

### The lipophilicity of *C. parakroppenstedtiii* and its association with infection

**Fig. 5:**
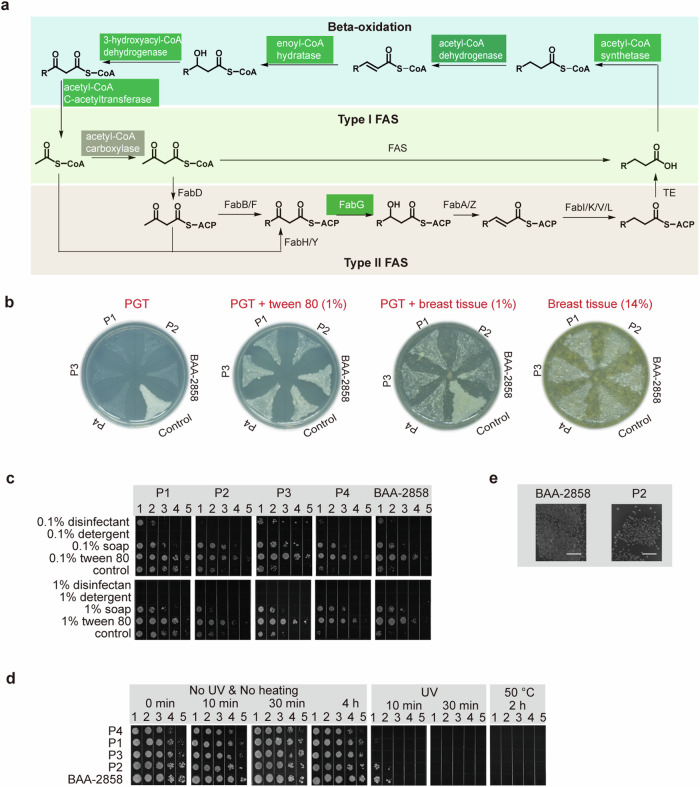
Lipophilicity of *C. parakroppenstedtii* and its possible infection route. **a** Biosynthetic and oxidative pathways of fatty acids in bacteria. The green-labeled genes were predicted by a Kyoto Encyclopedia of Genes and Genomes (KEGG) orthology search of the genome of *C. parakroppenstedtii*, and the alpha and beta subunits of acetyl-CoA carboxylase (gray) were predicted by KEGG orthology search of the genome of *C. parakroppenstedtii*; however, it is uncertain whether acetyl-CoA carboxylase can fully function. The unlabeled genes were not predicted. **b** Growth status of *C. parakroppenstedtii* and *C. amycolatum* in different culture media. P4: *C. parakroppenstedtii* P4; P3: *C. parakroppenstedtii* P3; P1: *C. parakroppenstedtii* P1; P2: *C. parakroppenstedtii* P2; BAA-2858: *C. parakroppenstedtii* BAA-2858; Control: *C. amycolatum*. **c** Growth status of *C. parakroppenstedtii* in BHIY medium supplemented with different washing agents at 0.1% or 1%. 1–5, 10-fold serial dilutions of sample. **d** The growth status of *C. parakroppenstedtii* after UV irradiation or drying. 1–5, 10-fold serial dilutions of sample. **e** Growth status of *C. parakroppenstedtii* after natural shade drying. Scale bar represents 5 mm

Next, we analyzed the genomes of all collected *C. kroppenstedtii*-like strains. A Kyoto Encyclopedia of Genes and Genomes orthology search showed that, except 3-oxoacyl-ACP reductase, no type I or type II fatty acid synthases were predicted (Fig. 5a), indicating that they may lack the ability to synthesize fatty acids from acetyl-CoA/malonyl-CoA. However, all strains possess all the genes required to oxidize fatty acids, indicating that they can obtain fatty acids from the environment to survive. However, we did not confirm whether non-*C. parakroppenstedtii* strains can cause GLM; therefore, we focused only on the relationship between fatty acids in the breast and *C. parakroppenstedtii*. We cultured five *C. parakroppenstedtii* strains and *C. amycolatum* (with fatty acid synthase verified by genome sequencing) in different culture media and found that *C. parakroppenstedtii* can use fatty acids from mammary tissue as a fatty acid source, and that mammary tissue can provide all the nutrients needed for the growth of *C. parakroppenstedtii* (Fig. 5b), which may be a factor in attracting *C. parakroppenstedtii* to infect the breast.

Considering the undergarment’s direct contact with the female breast, we suspected that it may be able to spread from the undergarments to the breast if *C. parakroppenstedtii* can survive in the undergarment washing and drying processes. In general, laundry detergents (mainly surfactants), disinfectants (mainly p-chlorocresol), and soaps (mainly fatty acyl metal salts) are used as washing agents. The drying process can be sun, machine, or shade drying. Therefore, we investigated whether *C. parakroppenstedtii* could be completely removed during these processes.

To investigate the effects of different washing agents on the removal of *C. parakroppenstedtii*, we used BHIY medium as the control group and added 0.1% (the common concentration for laundry) disinfectants, detergents, soaps, and Tween 80 to the medium as the experimental group to explore whether *C. parakroppenstedtii* could survive under these conditions. The results showed (Fig. 5c) that after 120 h, the five strains of *C. parakroppenstedtii* survived despite the absence of additional fatty acids in the control medium, indicating that *C. parakroppenstedtii* can survive for a long time without fatty acids, but cannot proliferate under such conditions. *C. parakroppenstedtii* did not grow in the detergent group, indicating that detergents can kill *C. parakroppenstedtii*. In the disinfectant group, only *C. parakroppenstedtii* P2 was completely killed, while the remaining strains were not. Notably, the abundance of *C. parakroppenstedtii* was higher in the soap group than the control group, suggesting that *C. parakroppenstedtii* can use fatty acyl metal salts in soap as a source of fatty acids and that soap can promote its proliferation. To confirm whether soap indeed does not kill *C. parakroppenstedtii*, we increased the concentration of all washing agents from 0.1% to 1%. The results showed (Fig. 5c) that 1% detergent and 1% disinfectant were sufficient to kill *C. parakroppenstedtii*, whereas 1% soap still did not kill *C. parakroppenstedtii*. These results strongly suggested that, if the undergarment is contaminated with *C. parakroppenstedtii*, using soap as a washing agent may fail to kill *C. parakroppenstedtii* and may promote its proliferation.

Next, to explore whether different drying methods could remove *C. parakroppenstedtii*, *C. parakroppenstedtii* was tested under simulated sun drying (UV), machine drying (50 °C, 2 h), and shade drying (no UV or heat) conditions. No apparent change in *C. parakroppenstedtii* growth was observed after shade drying for 4 h, but all *C. parakroppenstedtii* were killed after UV irradiation for 30 min or heating in a 50 °C oven for 2 h (Fig. 5d), demonstrating that *C. parakroppenstedtii* can be killed by sun drying or machine drying. To further determine whether *C. parakroppenstedtii* could be removed if the undergarment was shade dried, we placed two sterile cloth strips in a PBS solution containing *C. parakroppenstedtii* P2 and *C. parakroppenstedtii* BAA-2858, soaked the cloth strips thoroughly, and placed them in sterile Petri dishes. After 72 h of shade drying, the cloth strips were placed in contact with BHIY medium containing Tween 80. The results showed *C. parakroppenstedtii* colonies (demonstarted by 16S rRNA gene sequencing) in the contact areas of the cloth strips (Fig. 5e). Collectively, these results suggested that improper laundry practices may lead to the transmission of *C. parakroppenstedtii* to humans via undergarments. Our results suggest that washing undergarments in a non-soapy environment and drying them with UV or heat may prevent infection with *C. parakroppenstedtii*.

### The possible mechanisms of GLM caused by *C. parakroppenstedtii*

**Fig. 6:**
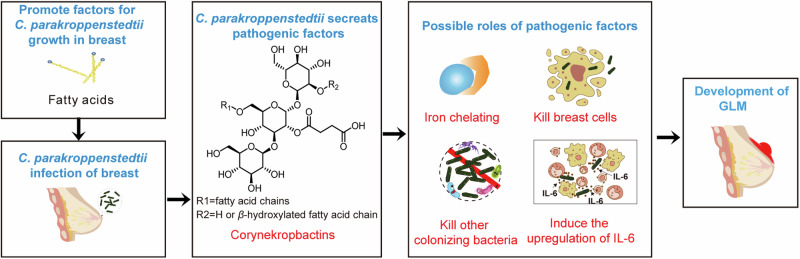
Hypothesis of the possible process of GLM development. This figure illustrates that fatty acids in the breast facilitate the infection of *C. parakroppenstedtii*, which subsequently secretes corynekropbactins. Corynekropbactins may then act as iron chelators, leading to the death of breast cells and other mammary-gland-colonizing-bacteria, and inducing the upregulation of IL-6. These multiple roles may contribute to the development of GLM

Based on the results of this study, we propose a possible process for the development of GLM (Fig. 6). Because *C. parakroppenstedtii* requires fatty acids to survive and proliferate, fatty acids in the breast and milk are factors that attract and promote its proliferation and colonization. *C. parakroppenstedtii* may then secrete corynekropbactins to chelate iron from the host, which may kill mammary cells and other mammary-gland-colonizing bacteria (e.g., *lactococcus*), thus establishing its colonial dominance. Moreover, corynekropbactins can induce inflammatory responses and contribute to the upregulation of IL-6. Eventually, the proliferation of *C. parakroppenstedtii* and the inflammatory response lead to the development of GLM.

## Discussion

GLM has become increasingly prevalent. The disease is most commonly reported in China, which may be attributed to China’s proactive measures, including the organization of specialized conferences on GLM, the formation of expert groups, and the development of expert consensus guidelines. These initiatives have significantly enhanced the understanding and awareness of GLM among Chinese healthcare professionals. In contrast, the paucity of international conferences on GLM may contribute to the limited recognition and underreporting of the disease in other regions. Consequently, there is a lack of a unified global understanding of GLM, with various national research teams holding disparate views. In this study, we investigated the pathogenicity of *C. parakroppenstedtii* in GLM in the manner of a detective. First, we identified the “suspect” using a probing tool (sequencing). Subsequently, the “crime scene” was reproduced using in vitro and in vivo experiments, and *C. parakroppenstedtii* was identified as the pathogenic bacterium of GLM. Based on the drug sensitization results of *C. parakroppenstedtii*, and utilizing a retrospective study in conjunction with a comprehensive literature review, we suggested an efficacious, targeted antibiotic treatment strategy for GLM. We then identified corynekropbactins as one of the “murder weapons” in *C. parakroppenstedtii* for the first time. The corynekropbactins that may chelate iron, kill mammary cells to obtain nutrients, and potentially kill other mammary-gland-colonizing bacteria (e.g., *lactococcus*), thus establishing their colonial dominance. Moreover, this pathogen induces an inflammatory response and contributes to the upregulation of the cytokine IL-6. Next, we proposed the prevalent lipophilicity of *C. parakroppenstedtii* as its “criminal motive”.

Previous studies have reported the isolation of *C. kroppenstedtii* from patients with GLM; however, its detection frequency has been relatively low. In our study, *C. kroppenstedtii*-like strains were cultured in only 5% of samples from patients with GLM, but 81% of samples from patients with GLM contain *C. kroppenstedtii*-like strains based on 16S rRNA gene sequencing. There are several possible reasons for this discrepancy. First, *C. kroppenstedtii*-like strains require exogenous fatty acids for growth and do not grow easily in commonly used culture media. Second, few infected bacteria can be cultured using traditional culture methods and dead bacteria cannot be cultured, whereas the sequencing method indiscriminately identifies both live and dead bacteria. Third, *Corynebacterium* species that have been identified as pathogenic bacteria in laboratory testing, such as *C. diphtheriae*, are reported as pathogenic, whereas other *Corynebacterium* species are generally considered to be colonizing bacteria or contaminating bacteria and thus, are not reported to clinicians. Although the pathogenicity of *C. parakroppenstedtii* is controversial, our study is the first to confirm the pathogenicity of *C. parakroppenstedtii* at the in vivo, in vitro, and molecular levels.

It is important to note that our study only confirmed that *C. parakroppenstedtii* can cause GLM. Whether bacterial infection is the only causative agent of GLM and whether there are other pathogenic bacteria have not been fully determined, and the precise infectious dose of *C. parakroppenstedtii* required to manifest the GLM phenotype in humans warrants further investigation. Nevertheless, some correlations can be found between *C. parakroppenstedtii* being the pathogenic bacteria of GLM and previously reported causes of GLM. For example, previous studies have suggested that GLM may be associated with pre-existing hyperprolactinemia,^12^ drug psychiatric disorders,^10^ and milk stasis.^7^ Medications that induce hyperprolactinemia include those used to treat psychiatric disorders, such as risperidone. Therefore, these previous studies indicate that GLM is associated with excessive lactation in patients. From our findings, we can infer that nutrients in milk, such as fatty acids, are essential for the growth of *C. parakroppenstedtii*. Therefore, factors that lead to high lactation might attract *C. parakroppenstedtii* colonization and increase the risk of developing GLM.

Based on the findings of this study, several avenues for future research merit further exploration. First, subsequent investigations should focus on elucidating the biosynthetic genes responsible for the production of corynekropbactins, which could provide critical insights into the molecular mechanisms underpinning corynekropbactin biosynthesis and potentially identify novel targets for therapeutic intervention. Second, given that patients diagnosed with GLM exhibit an elevated risk of developing breast cancer compared to non-GLM controls, our findings also imply that a substantial proportion of GLM cases may be attributable to infections by *C. parakroppenstedtii*. Therefore, the potential correlation between *C. parakroppenstedtii* infections and breast cancer necessitates further investigation. Specifically, it is crucial to explore whether there exists an association between the pathogenic mechanisms of corynekropbactins (its role in iron chelating, kill breast cells, modulation of the local microbiota, and induction of inflammatory cytokine upregulation) and the pathogenesis of breast cancer. Understanding these interactions at a molecular level could provide critical insights into the etiopathogenic links between GLM and breast cancer, thereby informing both preventative and therapeutic approaches. Furthermore, our research suggests that therapeutic regimens incorporating rifampicin may exhibit enhanced efficacy in the management of GLM. However, it is need to conduct prospective studies grounded in bacterial precision diagnostics to inform and tailor individualized treatment strategies for GLM. In addition, differences in economic status and behavioral habits may lead to differences in living conditions and lifestyles. Our study confirmed the lipophilicity of *C. parakroppenstedtii* and we hypothesize that fat-rich environments may promote bacterial infections, such as washing undergarments with soap. Therefore, whether the frequent occurrence of GLM in some regions is related to the method of washing undergarments or other environmental factors or habits that can contribute to fatty acid-rich environments for the growth of *C. parakroppenstedtii* warrants further epidemiological investigation.

There are still some unanswered questions arising from our study, such as whether there is a difference in the pathogenicity of GLM between the five *C. parakroppenstedtii*-like species, whether other bacteria besides *C. parakroppenstedtii* can cause GLM, whether GLM is affected by other non-bacterial infection factors, and what are the other pathogenic factors of *C. parakroppenstedtii*. However, based on our results, we suggest that early identification and targeted treatment of *C. parakroppenstedtii* infection are essential for breast health. In future clinical treatments, clinicians should initially determine whether the patient has GLM based on changes in inflammatory factors and iron levels in the patient, and then use more sensitive PCR or NTS to identify whether the infectious bacteria are *C. parakroppenstedtii*. If the pathogen is confirmed to be *C. parakroppenstedtii*, targeted treatment with suitable antibiotics (e.g., rifampicin) is a better way to cure the patients.

## Materials and methods

### Human samples and selection criteria

Clinical specimens used for sequencing were obtained from patients with GLM who underwent surgery or biopsy at the Renmin Hospital of Wuhan University. Detailed clinical information of the patients is shown in Supplementary Table 1. The tissue scrolls and sections used for H&E staining and immunofluorescence staining were from formalin-fixed paraffin-embedded mammary biopsy specimens provided by the Department of Pathology, Renmin Hospital of Wuhan University. Paracancerous tissue (normal) was defined as histologically normal adjacent breast tissue. Patients with GLM, fibroadenoma, or breast cancer were diagnosed based on pathological evaluation of fine-needle aspiration biopsy or surgical specimens. None of the patients with fibroadenoma or GLM had a history of malignant tumors. Patients with breast cancer who received neoadjuvant therapy were also excluded. The complete clinical information for these patients is presented in Supplementary Table 4.

Informed consent was acquired from all patients at Renmin Hospital of Wuhan University. Because only retained bacteria from Zhongnan Hospital of Wuhan University were used in this study, informed consent was waived for these patients. The study protocols were approved by the Ethics Committee of Renmin Hospital of Wuhan University (WDRY2020-K194 and WDRY2021-K093) and the Ethics Committee of Zhongnan Hospital of Wuhan University (2022225 K). All medical records were collected by a team of professional clinicians. Clinicians collected clinical information from the electronic medical records of the hospital information system (HIS). After exporting patients’ existing medical records from the HIS, we developed a standardized data collection form by extracting key information, such as clinical testing and culture results. These data were independently reviewed by two researchers to ensure the accuracy of data collection. If key information could not be obtained from the electronic medical records, the researchers collected it through communications with the attending doctors and other medical staff. If the data were still missing, the field was recorded as “unknown.”

### H&E staining

Human and rat mammary specimens were fixed overnight in phosphate-buffered 10% formalin. After dehydration, the samples were incubated in paraffin at 58 °C overnight and cast into molds covered with paraffin. Tissue sections were then prepared at a thickness of 5–10 μm. Paraffin sections were rehydrated with a decreasing gradient of ethanol concentrations and then stained with hematoxylin solution (0.1% hematoxylin, 5% Kal[SO_4_]_2_, and 0.02% KIO_3_). Counterstaining was performed by incubating the slides in eosin solution (1% eosin). Slices were dehydrated and cleared in xylene before mounting with neutral gum. H&E-stained images were acquired using a Panoramic MIDI system (3DHISTECH, Budapest, Hungary).

### Immunofluorescence staining of tissues from patients and rats

Nuclei were counterstained with 4′,6-diamidino-2-phenylindole. The images were captured using a panoramic MIDI slide scanner (3DHISTECH). Prior to immunofluorescence staining, mammary tissues extracted from animals and patients were fixed in 10% buffered formalin phosphate solution and embedded in paraffin according to standard protocols. Before antigen retrieval, the paraffin sections were dewaxed and rehydrated. Antigen retrieval was performed in ethylenediaminetetraacetic acid (EDTA) buffer (pH 8), and the sections were rinsed with PBS. The sections were then blocked using a blocking buffer for 30 min at room temperature and treated at 4 °C overnight in a humidified chamber with the primary antibody. Specific primary antibodies against IL-2 and IL-6 were obtained from Affinity Biosciences (OH, USA; AF5105, rabbit, 1:200; DF6087, rabbit, 1:200); IL-4 from Bo Ao Sen Biotechnology (Beijing, China; bs-0581R, rabbit, 1:100); IL-10 and IFN-γ from ProteinTech (Wuhan, China; 60269-1-Ig, mouse, 1:100; 15365-1-AP, rabbit, 1:100); and TNF-α from Boster Biological Technology (Wuhan, China; BA0131, rabbit, 1:100). After washing three times with PBS for 5 min each, the sections were incubated with a horseradish-peroxidase-conjugated secondary antibody for 50 min at room temperature in the dark. Quantitative image analysis was performed using ImageJ software (National Institutes of Health, Bethesda, MD, USA).

### 16S rRNA metagenomic sequencing

Samples were collected from patients and transported to the clinical laboratory within 2 h. All samples were then frozen immediately and stored at −80 °C prior to analysis. Each tissue sample was cut into small pieces using sterile scissors and homogenized. DNA was directly extracted from 200 μL of pus or pretreated rinse fluid using the QIAamp DNA Microbiome Kit (Qiagen, Hilden, Germany) following the manufacturer’s instructions.

The V3–V4 region of the bacterial 16S ribosomal RNA genes was amplified by PCR (95 °C for 3 min, followed by 35 cycles at 98 °C for 20 s, 58 °C for 15 s, and 72 °C for 20 s and a final extension at 72 °C for 5 min) using the barcoded primers, 341 F 5′-CCTACGGGRSGCAGCAG-3′ and 806 R 5′-GGACTACVVGGGTATCTAATC-3′. PCR was performed in a 30 μL mixture containing 15 μL of 2× KAPA Library Amplification ReadyMix, 1 μL of each primer (10 μM), 50 ng of template DNA, and ddH_2_O. Negative controls, consisting of empty sterile storage tubes, were processed for DNA extraction and amplification using the same procedures and reagents used for the tissue samples. No detectable amplification was observed in the negative controls. Amplicons were extracted from 2% agarose gels and purified using the AxyPrep DNA Gel Extraction Kit (Axygen Biosciences, Union City, CA, USA) according to the manufacturer’s instructions and quantified using a Qubit® 2.0 instrument (Invitrogen, Carlsbad, CA, USA). All quantified amplicons were pooled to equalize their concentrations for sequencing using an Illumina MiSeq (Illumina, Inc., San Diego, CA, USA). Paired-end reads of 300 bp were overlapped on their 3′ ends for concatenation into original longer tags using PANDAseq (https://github.com/neufeld/pandaseq, version 2.9).

### Bioinformatic analysis

The 16S rRNA sequences were clustered into operational taxonomic units using VSearch software and identity criteria of 97%. The Shannon index was used to compute the alpha diversity of the samples, and the Mann–Whitney U test was used to examine the differences in the Shannon index values among groups. We used the PERMANOVA test based on principal coordinate analysis with Bray–Curtis dissimilarity to compare the community composition of the microbiota among groups. The vegan package was used to perform alpha- and beta-diversity analyses in R. Linear discriminant analysis effect size was used to detect the taxa responsible for the different microbiota compositions. The logarithmic LDA cutoff score was set at 4.

### Isolation and culture of *C. parakroppenstedtii*

All the tissue or pus samples in our study were collected from patients with GLM who underwent routine culture in the clinic. The samples were incubated under aerobic and anaerobic conditions using one Columbia blood agar plate and one chocolate agar plate each at 35 °C. After observing the colony morphology of the microorganisms, suspected pathogenic microorganisms were selected for strain identification using matrix-assisted laser desorption/ionization time-of-flight (MALDI-TOF) MS analysis. For microorganisms that could not be differentiated by MALDI-TOF MS, biochemical reactions were used for strain identification. All 17 *C. kroppenstedtii*-like strains collected at the Zhongnan Hospital were isolated using routine culture methods.

For P1–P4 strain isolation, breast pus from the patients was used to inoculate BHIY medium (37 g/L brain-heart infusion broth and 10 g/L yeast extract) with 1% Tween 80, and 2% agar was added to make a solid plate. The plate was incubated at 37 °C in a 5% CO_2_ atmosphere for 48 h. The colonies on the plates were then isolated and identified as *C. parakroppenstedtii* using whole-genome sequencing. The bacteria were then preserved at −80 °C in 20% glycerol.

Subsequently, *C. parakroppenstedtii* was grown on BHIY with 1% Tween 80 culture plates to form single colonies. For subsequent interactions with animals or cells, one single colony was inoculated in 5 mL of BHIY with 1% Tween 80 liquid medium at 30 °C for 48 h, and all the bacteria were then transferred to 200 mL of BHIY with 1% Tween 80 liquid medium for culture at 30 °C until the optical density at 600 nm (OD_600_) = 2–3. Multiple vials of bacteria were cultured with the desired amount of cells and then collected by centrifugation and resuspended in PBS to a bacterial concentration of 1 × 10^5^ colony-forming units (cfu)/µL, which was then used for subsequent animal experiments or cell experiments. *C. parakroppenstedtii* was inactivated by pasteurization for 40 min at 70 °C for obtain heat-killed bacteria, and follow-up culture was used to confirm that all bacteria were inactivated.

For subsequent corynekropbactin isolation experiments, several single colonies were inoculated in 10 mL of low-iron PGT medium^43^ or BHIY medium with 1% soy oil at 37 °C for 48 h, and then all the bacteria were transferred to 800 mL of low-iron PGT medium or BHIY medium with 1% soy oil for culture at 37 °C for 60 h. Multiple vials of culture supernatants were used for subsequent corynekropbactin extraction experiments. For corynekropbactin production analysis, several single colonies were inoculated in 20 mL of iron-free PGT medium with 1% soy oil at 37 °C for 48 h. Then, 1 mL of the bacteria were transferred to 100 mL of PGT medium with 1% soy oil and iron ions at different concentrations (Fe-free, 10 μM Fe^2+^, 10 μM Fe^3+^). After 80 h of fermentation at 37 °C, 50 mL of the culture broth was centrifuged at 7,000 rpm for 15 min for subsequent quantification.

To test the effects of different fatty acid sources on bacterial growth, single colony were delineated on PGT solid medium (low-iron PGT medium with 2% agar) with different fatty acid sources or on 14% parametrial breast cancer tissue.

### Whole-genome sequencing of *C. kroppenstedtii*-like strains

The genomic DNA of *C. kroppenstedtii*-like strains was extracted using a Bacterial Genomic DNA Extraction Kit (Tiangen, Beijing, China) following the manufacturer’s instructions. Whole-genome sequencing was performed and the genomes were assembled by GrandOmics (Wuhan, China) using the T7 (MGI, Shenzhen, China) and PromethION (Oxford Nanopore, Oxford, UK) platforms. To analyze genome similarity, the whole-genome sequences of the five strains were aligned in a pairwise fashion using *bwa-mem* to calculate similarities at the nucleotide level.

### Cell experiments

The human normal breast cell line MCF-10A was obtained from Procell (Wuhan, China) and cultured at 37 °C and 5% CO_2_ in MCF-10A cell special culture medium (Procell). The cells were maintained in the logarithmic growth phase. The medium was renewed every 2 d, and the cells were trypsinized with 0.25% trypsin-EDTA and subcultured in the same medium.

For interaction with *C. parakroppenstedtii*, MCF-10A cells were seeded in six-well plates (to measure cytokines) or T75 bottles (to detect corynekropbactins) and allowed to adhere overnight until the cell monolayer occupied ~90% of the vessel. In a six-well plate, 9 × 10^7^ *C. parakroppenstedtii* cells were added into each well (2 mL) and cultured at 37 °C and 5% CO_2_ for 24 h, or 1 × 10^6^ live or heat-killed *C. parakroppenstedtii* cells were added to each well (2 mL) and cultured at 37 °C and 5% CO_2_ for 12 h. In a T75 bottle, 2 × 10^6^ *C. parakroppenstedtii* cells were added and cultured at 37 °C and 5% CO_2_ for 72 h. The cell culture supernatant was collected by centrifugation at 12,000 rpm for 10 min. One milliliter of supernatant from a six-well plate was collected and stored at −80 °C until further cytokine testing was performed, and 15 mL of supernatant from a T75 bottle was collected and stored at −80 °C until further corynekropbactins testing was performed.

For interactions with **1** and **2**, cells were seeded in 96-well plates at a density of 4 × 10^3^ cells/well. After 24 h, compounds **1** and **2** were added to the cells. Cytotoxicity was tested in MCF-10A cells using Cell Counting Kit-8 (CCK-8) (Dojindo Laboratories, Kumamoto, Japan). The blank control group was prepared by adding medium only or medium with 0.05% DMSO. Each group comprised six replicates. After 48 h, 80 μL of the cell supernatant was removed, and an equal amount of fresh medium was added to the same well. Ten microliters of CCK-8 reagent was then added and reacted for 2 h in the dark. The OD was measured at 450 nm using a microplate reader (BioTek, Winooski, VT, USA). Cell viability (%) was calculated as (OD treated - OD blank)/(OD control - OD blank) × 100%. The IC_50_ values for different drugs were determined using SPSS (IBM, Armonk, NY, USA). Subsequently, changes in cytokine levels were measured 6 h after the addition of **1** or **2** at their IC_50_ concentration (300 μmol/L and 50 μmol/L, respectively). Each group was comprised of four replicates. Eighty microliters of cell supernatants were removed and stored at −80 °C until further cytokine testing was performed.

### Animal experiments

All animal studies were reviewed and approved by the Laboratory Animal Welfare and Ethics Committee of Renmin Hospital of Wuhan University (Issue Nos. 20200702 and 20211001). Female Sprague–Dawley rats (8 weeks old for GLM-related experiments) used in this study were obtained from Hunan Silaike Jingda Laboratory Animal Co., Ltd. (Changsha, China). All animals were maintained on a 12-h light-dark cycle and housed in groups of three or four for at least 5 d before the experiments. All rats were provided with a standard laboratory diet and distilled water.

On the day of infection, the rats were anesthetized by continuous 2% isoflurane inhalation (1% oxygen). After anesthetization, approximately 1 × 10^7^ cfu/rat of *C. parakroppenstedtii* or 100 μL/rat of PBS were injected as indicated orthotopically into the fourth mammary fat pad with an insulin syringe. The rats were sacrificed at the indicated time points (3, 8, and 10 d).

All rats were euthanized by carbon dioxide inhalation and blood was collected in a heparin solution by cardiac puncture, according to the experimental design. Mammary tissues were collected using sterile dissection tools, according to the experimental design. Infections were conducted in 5–12 animals per group, as indicated.

### Analysis of cytokine levels in serum and cell culture

The levels of the cytokines IL-2, IL-4, IL-6, IL-10, IFN-γ, and TNF-α in rat serum were measured using multiplexed sandwich enzyme-linked immunosorbent assays (ELISA) via custom-made magnetic Luminex multiple assays (LXSARM-06, R&D Systems, Minneapolis, MN, USA) according to the manufacturer’s instructions, and the results were read on a Lumine X-200 instrument (Luminex, Austin, TX, USA).

The levels of TNF-α, IL-2, IL-4, IL-6, IL-10, and IFN-γ in MCF-10A cell culture were measured using an NMPA-approved kit (human Th1/Th2 subgroup detection kit; Cellger, Hangzhou, China) via a flow fluorescence assay (FACSCalibur, BD Biosciences, San Jose, USA) according to the manufacturer’s instructions.

### Assay of iron-related factors in rats

Rat serum samples were analyzed using an ADVIA 2400 Clinical Chemistry System (Siemens, USA) with Iron_2 reagents (Siemens Healthineers, USA). The total iron-binding capacity (TIBC) was measured by an ADVIA 2400 Clinical Chemistry System (Siemens) with TIBC reagents (Siemens). Transferrin saturation was calculated using the following equation: [total iron]/[TIBC] × 100.^44^ Ferritin levels were measured using an ELISA kit (Cusabio, Wuhan, China).^45^

### High-performance liquid chromatography analysis of the bacterial supernatant and culture medium

The culture supernatant and blank culture medium were absorbed by polystyrene-divinylbenzene copolymers (Amberlite XAD 16, Macklin) to enrich corynekropbactins and then eluted with methanol. High-performance liquid chromatography (HPLC) analysis was performed using an ODS column (ZORBAX SB-C18, 4.6 × 250 mm, 5 μm; 1 mL/min flow rate) with gradient elution. The UV spectra were recorded on a DAD instrument in the wavelength range of 200–600 nm. HPLC parameters were as follows: solvent A, H_2_O with 0.1% TFA; solvent B, 0.1% TFA in acetonitrile (ACN); gradient at a constant flow rate of 1 mL/min as follows: 0–60 min, 10–100% B and 60–70 min, 100% B; and detection by UV spectroscopy at 210 nm.

### Extraction and purification of corynekropbactins

After fermentation of *C. parakroppenstedtii* P1 in PGT or BHIY medium with soy oil, the culture broth was centrifuged at 7,000 rpm for 15 min. The resulting supernatant was stirred with preprocessed XAD-16 resin under strain cultivation conditions for 2 d. The resin was then filtered and the material was eluted with pure methanol. The crude extract was concentrated under reduced pressure.

To purify corynekropbactins, the crude extracts were eluted using a normal-phase silica gel column. Fractions 18 to 24 (CH_2_Cl_2_:CH_3_OH, 0:1) were subjected to Sephadex LH-20 column chromatography and eluted with CH_3_OH to yield 40 fractions (10 mL/fraction). Fractions 8 to 15 were separated using semipreparative HPLC (ZORBAX SB-C18, 5 µm, 9.4 × 250 mm; gradient elution 0–60 min, 10–100% ACN and 60–70 min, 100% ACN; H_2_O with 0.1% formic acid [FA]; and flow rate, 3 mL/min) to yield compounds **1–8**.

### CAS assay

CAS agar was prepared according to previously reported methods ^46^with minor modifications. To obtain 250 mL of CAS, the following reagents were used. Reagent A was prepared by mixing solution 1 (0.06 g of CAS dissolved in 50 mL of ddH_2_O) with 9 mL of solution 2 (0.0027 g of FeCl_3_ ∙ 6H_2_O dissolved in 10 mL of 10 mM HCl), which was then mixed with solution 3 (0.073 g of hexadecyl trimethyl ammonium bromide dissolved in 40 mL ddH_2_O) to obtain a blue dye, which was then autoclaved. Reagent B was prepared by dissolving 8.06 g of 1,4-piperazinediethanesulfonic acid in 225 mL of ddH_2_O. After adjusting the pH to 6.8, 3.75 g of agar was added, and the mixture was autoclaved and cooled to 50 ~ 60 °C. Subsequently, 25 mL of reagent A was slowly added to 225 mL of reagent B and mixed thoroughly. The solidified CAS agar plates were punched to a diameter of 5 mm, filled with 50 µL of sample, and then incubated at 37 °C for 2 d.

The ability of the corynekropbactins to chelate iron ions was determined using a previously reported protocol.^47^ For qualitative experiments, a liquid-type CAS assay was used. The CAS assay solution was prepared using the same method as that for CAS agar, but without the addition of agar. Sample solutions (100 µL) were mixed with the same volume of CAS assay solution in 96-well microtiter plates. To examine siderophores that induce a color change in the CAS solution, after 12 h of incubation at 30 °C, the OD_630 nm_ values of the CAS solution with the solvent methanol (Ar) and CAS solution with compound sample (As) were measured using a Tecan Spark Spectrophotometer (Tecan, Groedig, Austria). In addition, the OD_630 nm_ values of a series of two-fold dilutions of EDTA in methanol (12 µM–200 µM) were measured to draw a calibration curve to further compare the Fe(III)-binding activities with the compound samples.

### Determination of the ability of corynekropbactins to chelate metal ions

For metal chelating experiments, 60 µL of 600 µM purified corynekropbactin I, 400 μM corynekropbactin II, or methanol were incubated with an equal volume of 400 µM metal ion solutions (FeCl_3_, FeSO_4_, CuSO_4_, MnCl_2_, NiCl_2_, CoCl_2_) at 30 °C for 2 h, with 50% methanol used as the blank background. UV–vis spectra were recorded using a Multiskan GO microplate reader (Thermo Fisher Scientific) with a 250–1,000 nm range and 2 nm bandwidth.

### Exploring the effects of ferric corynekropbactins on the growth of *C. parakroppenstedtii*

For growth experiments, several colonies of *C. parakroppenstedtii* P1were inoculated in 5 mL of iron-free PGT medium with 1% Tween 80 liquid medium at 37 °C until OD_600_ = 2–3. The cultures were centrifuged at 3,000 × g for 15 min at 4 °C, and an equal amount of PBS was added to resuspend the bacteria, the sample was then centrifuged to remove the supernatant. After washing three times with PBS, an appropriate volume of PBS was added to resuspend the bacteria to OD_600_ = 0.5, and diluted 1:100 into each well of a flat-bottom 96-well plate containing 200 μL of PGT medium with 10 µM of iron, corynekropbactins, or ferric corynekropbactins. Ferric corynekropbactins were prepared by reacting 3 mM **1** or **8** with equal volumes of 3 mM FeCl_3_ or FeSO_4_ solution for 3 h. Excess iron ions within the reaction system were removed by semipreparative HPLC (ZORBAX SB-C18, 5 µm, 9.4 × 250 mm; gradient elution 0–60 min, 10–100% ACN and 60–70 min, 100% ACN; H_2_O with 0.1% formic acid [FA]; and flow rate, 3 mL/min). The corynekropbactins and their iron chelates were lyophilized, decontaminated with UV for 2 h, and diluted with iron-free PGT medium containing 1% Tween 80 to 10 µM. The plates were then incubated at 37 °C for 48 h in a Tecan Spark plate reader (Tecan Group Ltd. Switzerland), shaken at 220 rpm for 30 min every hour, and assayed for the OD_600_. Three replicates were used for each fraction, and iron-free PGT medium containing 1% Tween 80 was used as the control.

### Antibacterial assays

For agar diffusion assays, the appropriate agar medium (GM17 agar medium for *L. lactis;* MRS agar medium for *Lactobacillus reuteri*; LB agar medium for *E. coli* and *C. glutamicum*) was cooled to 50 ~ 60 °C and mixed with 1% overnight-cultured bacteria. The solidified bioassay plates (containing 25 mL of medium) were punched with a diameter of 5 mm with sterile punches and filled with 50 µL of sample. After incubation under the appropriate conditions (30 °C overnight for *C. glutamicum*, 30 °C for 2 d for *L. lactis*, 37 °C overnight for *E. coli*, or 37 °C for 2 d for *Lactobacillus reuteri*), the zone of inhibition in each well was observed.

The MIC was determined as previously described.^48^ Overnight-cultured bacterial strains were adjusted to 5.0 × 10^5^ cfu/mL in culture medium (GM17 medium for *L. lactis*, MRS medium for *Lactobacillus reuteri*, and LB medium for *C. glutamicum*). A series of two-fold dilutions of each test compound was prepared. A total of 180 µL of the diluted bacteria and 20 µL of compound were mixed in 96-well microtiter plates and incubated under the corresponding conditions (30 °C overnight for *C. glutamicum* and *L. lactis* and 37 °C overnight for *L. reuteri*). Bacterial growth was monitored by measuring the OD_600 nm_ using a Tecan Spark Spectrophotometer. The MIC was defined as the lowest concentration at which no bacterial growth occurred.

### Liquid chromatography-tandem mass spectrometry analysis of the corynekropbactins

Fifteen milliliters of cell culture were absorbed with polystyrene-divinylbenzene copolymers (Amberlite XAD 16, Macklin) to enrich corynekropbactins, and then eluted with methanol. The eluted methanol was concentrated to 2 mL for liquid chromatography-tandem mass spectrometry (LC-MS/MS) analysis.

Breast tissue from rats was first cut into mung-bean-sized pieces, added to grinding beads, rapidly frozen in liquid nitrogen and quickly ground with a tissue grinder (65 Hz, interval of 10 s, repeated three times). Rapid freezing and grinding was repeated twice, and the tissue homogenate was obtained. Next, 1 mL of methanol was added for grinding and extraction, and the mixture was centrifuged at 10,000 r/min for 5 min to obtain the supernatant. All supernatant samples from same group were combined and concentrated to 200 μL for LC-MS/MS analysis.

Fifty milliliters of the bacterial culture broth supernatants were stirred with preprocessed 2% XAD-16 resin (w/v) under strain cultivation conditions for 8–12 h. The resins were filtered away and washed three times with deionized water, and the corynekropbactins were eluted with 6 mL of methanol and sonicated for 1 h. Two hundred microliters of eluent was used for for LC-MS/MS analysis.

LC-MS/MS datasets were acquired using a liquid chromatograph (Dionex Ultimate 3000, Thermo Fisher Scientific, CN) and a Q Exactive benchtop quadrupole-Orbitrap mass spectrometer (Q Exactive, Thermo Fisher Scientific), or a liquid chromatograph (ACQUITY UPLC H-Class PLUS, Waters, SG) and triple quadrupole mass spectrometer (TripleTOF 6600 + , AB Sciex, CA). Analytical-scale chromatographic separations were performed using a 2.1 mm × 150 mm reversed-phase column (Hypersil gold AQ, 2.1 × 150 mm column 0.2 mL/min) and binary solvent system comprising water (solvent A) and ACN (solvent B) gradient at a constant flow rate of 0.2 mL/min as follows: 0–30 min, 10–100% B; 30–50 min, 100% B. Negative-mode electrospray ionization was performed. The samples were analyzed in the m/z range of 50–1300 or 200–1500.

### Antibiotic susceptibility of *C. parakroppenstedtii*

The antibiotic susceptibility of the strains was determined using the Kirby–Bauer disk diffusion method, according to a previous study, with some modifications.^49^ McFarland turbidity was adjusted to 0.5, and the bacteria were inoculated using sterile cotton swabs on the surface of a Columbia blood agar plate (Dijing, Guangzhou, China). The following seven antibiotic disks (Oxoid, Hampshire, United Kingdom) were used: ciprofloxacin (5 µg), gentamicin (10 µg), metronidazole (5 µg), clindamycin (2 µg), rifampicin (5 µg), moxifloxacin (5 µg), and ceftazidime (30 µg). The antibiotic disks were placed on Columbia blood agar plates within 15 min of inoculation. The plates were incubated for 48–72 h at 30 °C in a 5% CO_2_ atmosphere. The diameters of the inhibition zones formed around the antibiotic disks were measured and interpreted according to the European Committee on Antimicrobial Susceptibility Testing guidelines (http://www.eucast.org).

### Whole-genome phylogenetic tree method

Multiple sequence alignments (MSAs) generated using GTDB-Tk software (version 2.1.0)^50^ were used to construct a phylogenetic tree for the whole genome. IQ-Tree (version 2.2.0.3)^51^ was used to calculate the maximum likelihood phylogeny for the MSAs with the following parameters: -alrt 1,000, -bb 1,000, and -nt AUTO. The best-fitting model determined using ModelFinder,^52^ JTT + F + I, was well supported by the Akaike information criterion (AIC), corrected AIC (AICc), and Bayesian information criterion values. Tree branches were tested using an SH-like aLRT with 1,000 replicates. Finally, a phylogenetic tree was generated using the ITOL-v5 online tool.^53^

### Exploring the influence of different washing agents on the growth of *C. parakroppenstedtii*

Fifty milliliters of *C. parakroppenstedtii* broth (BHIY culture medium with Tween 80) in the logarithmic growth phase was centrifuged at 3000 × g for 15 min at 4 °C, an equal amount of PBS was added to resuspend the cells, and each sample was then centrifuged to remove supernatant. After washing three times with PBS, an appropriate volume of PBS was added to resuspend the pellet to an OD_600_ of 4. Subsequently, 100 µL of OD_600_ = 4 seed bacteria was transferred to 10 mL of BHIY culture medium with different washing agents (detergent, soap, or disinfectant) and cultured at 30 °C for 96 h. BHIY culture medium was used as the control group, and BHIY with Tween 80 culture medium was used as the positive control group. The cultures were then serially diluted 10 times, and 2 µL of each diluted culture was spotted on a plate with BHIY with 1% Tween 80 and cultured at 30 °C for observation for 48–72 h.

### Exploring the influence of different drying methods on the growth of *C. parakroppenstedtii*

Fifty milliliters of *C. parakroppenstedtii* broth (BHIY culture medium with Tween 80) in the logarithmic growth phase was centrifuged at 3000 × g for 15 min at 4 °C, an equal amount of PBS was added to resuspend the cells, and each sample was centrifuged to remove the supernatant. After washing three times with PBS, an appropriate volume of PBS was then added to resuspend the pellet to an OD_600_ of 4. Next, 5 mL of bacteria suspension was placed in a sterile Petri dish to determine the effect of different drying methods on bacterial activity.

A Petri dish containing 5 mL of *C. parakroppenstedtii* with an OD_600_ of 4 was placed in a biological safety cabinet. The lid was removed, and the plate was irradiated with a UV lamp (20 W, 30 cm) for 10 or 30 min. A non-UV lamp was used for the control group. The bacteria were then serially diluted 10 times, and 2 µL of each diluted culture was spotted on a plate BHIY with 1% Tween 80 and cultured at 30 °C for observation for 48–72 h.

A Petri dish containing 5 mL of *C. parakroppenstedtii* with an OD_600_ = 4 was placed in a 50 °C oven for 2 h to simulate common machine drying conditions. An appropriate volume of PBS was then added to supplement the evaporated liquid, and the volume in the Petri dish was restored to 5 mL. Next, the bacteria were serially diluted 10 times, and 2 µL of each diluted culture was spotted on a plate with BHIY with 1% Tween 80 and cultured at 30 °C for observation for 48–72 h.

### Statistical analysis

Data are presented as the mean ± standard error of the mean, interquartile range, or standard deviation. Statistical significance was determined using the Kruskal–Wallis test, Wilcoxon signed rank test, Fisher’s exact test, and Dunn’s test, followed by the Benjamini–Hochberg correction, as appropriate. Two-sided *P*-values < 0.05 were considered statistically significant. Statistical analyses were performed using R (version 4.2.1), and graphical representations and data visualization were created using GraphPad Prism 8.2.1 (GraphPad, San Diego, CA, USA).

## Supplementary information

Supplementary information_marked up

Change of authorship request form

## Data Availability

16 s rRNA sequencing data have been deposited in the National Omics Data Encyclopedia (NODE, https://www.biosino.org/node) under project ID OEP004181. The aligned sequences of Corynebacterium strains have been deposited in the eLibrary of Microbial Systematics and Genomics (eLMSG, https://www.biosino.org/elmsg/index) under accession codes LMSG_G000026862.1-LMSG_G000026883.1. The data of HPLC and LC-MS have been deposited in the National Microbiology Date Center (NMDC, https://nmdc.cn/) under accession code NMDCX0001707. Other data can be obtained by contacting the corresponding author with suitable reasons.
