# Supplementary material for: Corynebacterium parakroppenstedtii secretes a novel glycolipid to promote the development of granulomatous lobular mastitis
Source: Signal Transduct Target Ther. 2024 Oct 21;9:292. doi: 10.1038/s41392-024-01984-0 (PMC11491465; doi:10.1038/s41392-024-01984-0)
Supplement: Supplementary file 1 — Supplementary information_marked up [file 41392_2024_1984_MOESM1_ESM.docx]

Supplementary Materials for

*Corynebacterium parakroppenstedtii* secretes a new glycolipid to promote the development of granulomatous lobular mastitis

Ran Liu, Zixuan Luo, Chong Dai, Yuchen Wei, Shuqing Yan, Xinwen Kuang, Kuan Qi, Aisi Fu, Yinxin Li, Shuai Fu, Zhengning Ma, Wen Dai, Xiao Xiao, Qing Wu, Haokui Zhou, Yan Rao, Jingping Yuan, Ting Shi, Zixin Deng, Chuang Chen, Tiangang Liu

Correspondence to: Tiangang Liu (liutg@whu.edu.cn), Chuang Chen (chenc2469@whu.edu.cn), or Zixin Deng (zxdeng@sjtu.edu.cn)

**This PDF file includes:**

Materials and Methods

Supplementary Text

Figures. S1 to S28

Tables S1 to S9

Materials and Methods

**Determination of the sugar sequences of 1 and 2**

The 1D-TOCSY spectrum of each sugar unit was obtained by comparing the coupling constants with a standard D-glucose spectrum ^1^, and both sugar units were identified as D-glucose.

**Determination of the fatty acid moieties of 1 and 2**

NaOH (0.8 mg, 20 µmol) was added to a solution of **2** (0.5 mg) in MeOH-H_2_O (2 mL, MeOH:H_2_O=5:1) at room temperature. The reaction mixture was allowed to warm to 42 °C for 2 h. The methanol was removed in vacuo, and the residual aqueous solution was partitioned with AcOEt (2 mL). Then, the organic phase was extracted with H_2_O (3 mL, two times). The combined aqueous extracts were acidified to pH 2 with 1 mol/L HCl. The aqueous phase was extracted with CH_2_Cl_2_, and the combined organic extract was analyzed by GC‒MS.

Supplementary Text

**Structure identification of corynekropbactins**

Compound **1** was isolated as a colorless oil. Its molecular formula, C_36_H_60_O_20_, was assigned by analysis of its HR–ESI–MS data [M－H]^－^ at m/z 811.3590 (Figure S10) and ^13^C NMR spectra (Fig. S11). A detailed analysis of the NMR spectra (Table S9) revealed that **1** contains a sugar sequence and two aglycones. Comprehensive analysis of the 1D (^1^H, ^13^C, and 1D-TOCSY) (Figs. S11–S15), and 2D (HSQC, HMBC, and ^1^H–^1^H COSY, respectively) (Figs. S16–S18) NMR spectra permitted assignment of the sugar sequence consisting of three D-glucose residues, and the aglycones were determined to be succinic acid (Agly′) and 5*E*-tetradecenoic acid (Agly″). The absolute configurations of the sugar units were determined using the coupling constants of the anomeric proton signals. glu′ and glu″ were found to be in the *α*-configuration based on the relatively small coupling constants (*J* = 3.8, 3.7 Hz) of the anomeric proton signals at *δ*_H_ 5.05 and 5.26 (Figs. S13-S14), while glu‴ was determined to be a *β*-configuration [*δ*_H_ 4.44 (*J* = 7.8 Hz)] (Fig. S15). Finally, glycosidic connections were determined using the HMBC spectrum (Fig. S17). The oligosaccharide moiety in **1** was identified as 1-*O*-(*α*-d-glucopyranosyl)-3-*O*-(*β*-d-glucopyranosyl)-*α*-d-lucopyranoside by the HMBC correlations from H- Glu′-1 to C-Glu″-1 and from H-Glu‴-1 to C-Glu″-3. The HMBC correlations from H- Glu″-2 to C-Agly′-1 and from H- Glu″-6 to C-Agly″-1 suggested that Agly′ was connected to C- Glu″-2 and Agly″ was connected to C- Glu″-6. Thus, the planar structure and absolute configuration of **1** were established. Compound **2** was isolated as a colorless oil. Its molecular formula, C_50_H_86_O_22_, was assigned by analysis of its HR–ESI–MS data [M－H]^－^ at m/z 1037.5498 (Fig. S19) and ^13^C NMR spectra (Fig. S20). A comparison of the 1D and 2D NMR spectra of **2** (Figs. S19–S27) and **1** revealed that both compounds shared the same oligosaccharide moieties and agly′, with the differences mainly located at the fatty acid moieties, as a new *β*-hydroxylated fatty acid chain was connected to C- Glu′-2 in **2**. However, because of the overlap of the NMR signals, the length of the fatty acid chain could not be determined by NMR spectroscopy. To identify the length of the fatty acid chain, alkaline hydrolysis was performed on **2**, which yielded (*Z*)-9-octadecenoic acid (Agly″) and (*S*)-3-hydroxydecanoic acid (Agly‴) by GC‒MS analysis. Thus, the structure of **2** was established.

Moreover, LC-MS analysis suggested that the *C. parakroppenstedtii* culture supernatant contained a series of corynekropbactins, although only **1** and **2** were present in sufficient quantities to obtain their NMR spectra, analysis of the secondary mass spectrum cleavage of the corynekropbactins allowed us to identify the remaining corynekropbactins (**3–8**). Notably, compounds **3–8** possessed a similar secondary mass spectrum as **1** and **2**, and shared several common daughter ions, m/z 485.15 and m/z 503.16, belonging to the trisaccharide, and m/z 101.02, belonging to succinic acid (Fig. S28), strongly suggesting that they have the lipopolysaccharide structures (with differences in R1 and R2) of **1** and **2**. Daughter ions at m/z 187.13, belonging to (*S*)-3-hydroxydecanoic acid, were present in **5–8** and **2**, but absent in **3–4** and **1**, indicating that R2 = (*S*)-3-hydroxydecanoic acid in **5–8** and **2**, as R2 = H in **3–4** and **1.** Moreover, the R1 moieties of **3–8** were deduced as (7*Z*,10*Z*)-7,10-hexadecadienoic acid, tetradecanoic acid, (5*Z*,8*Z*)-5,8-tetradecadienoic acid, (7*Z*,10*Z*)-7, 10-hexadecadienoic acid, dodecanoic acid, and tetradecanoic acid respectively, which was supported by the analysis of the culture medium (only stearic acid, palmitic acid, oleic acid, linoleic acid can be used as precursors for fatty acid chain biosynthesis) combined with derivation of the biogenic pathway and daughter ions m/z 251.2010, 227.2012, 223.1698, 251.2010, 199.1698, and 227.2018 in **3–8**, respectively. Accordingly, a series of corynekropbactins (**3–8**) was identified.


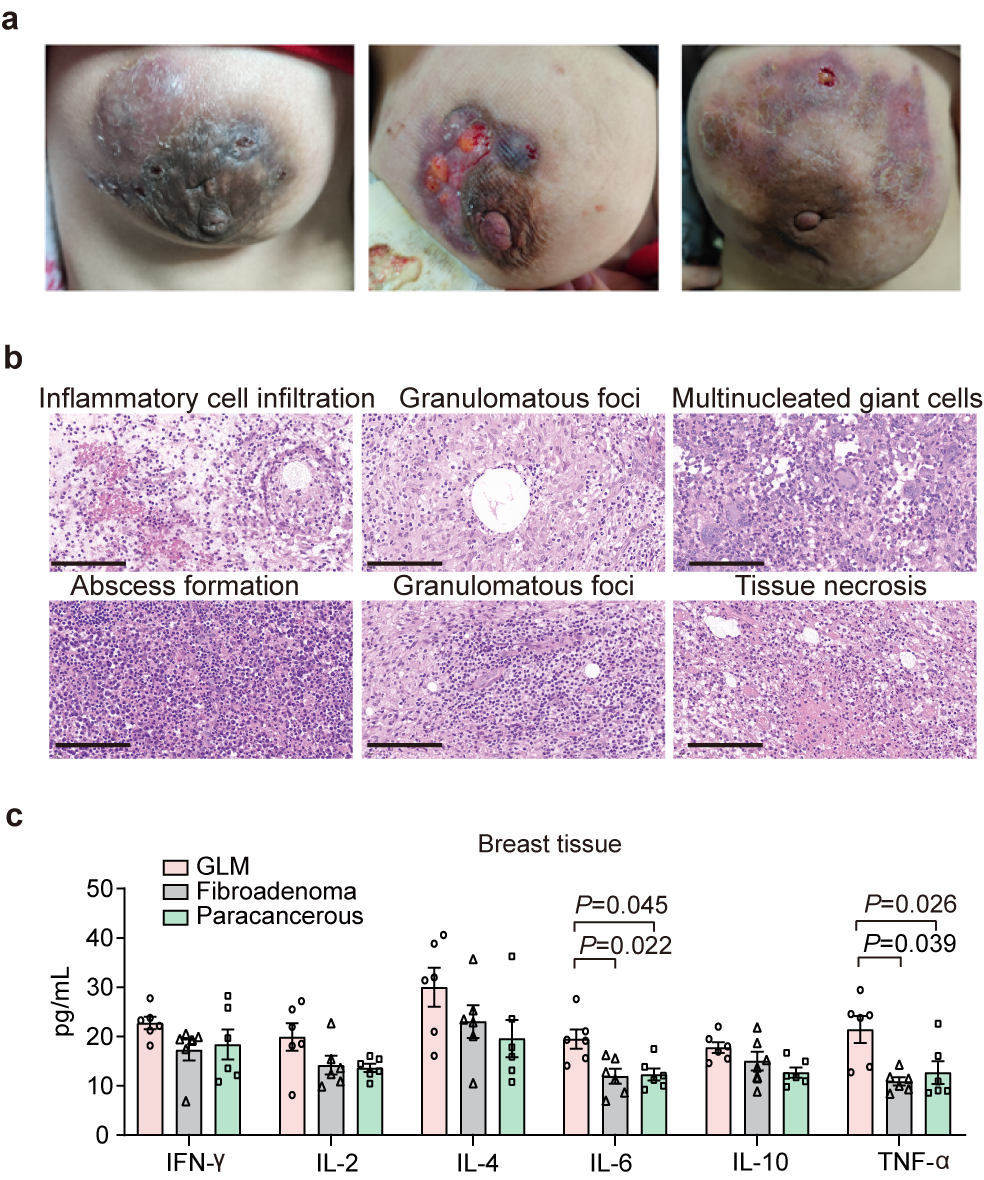


Figure. S1. Characteristics of patients with GLM.

(**a**) photos from patients with GLM. (**b**) Typical HE-stained pathological sections from patients with GLM. Scale bar represents 100 μm. (**c**) Concentrations of cytokines in the pathological tissues of patients with GLM, fibroadenoma tissues, and paracancerous tissues (n=6). Data are expressed as the mean ± SEM, and Dunn’s test with Benjamini-Hochberg correction for multiple comparison to calculate statistical significance.


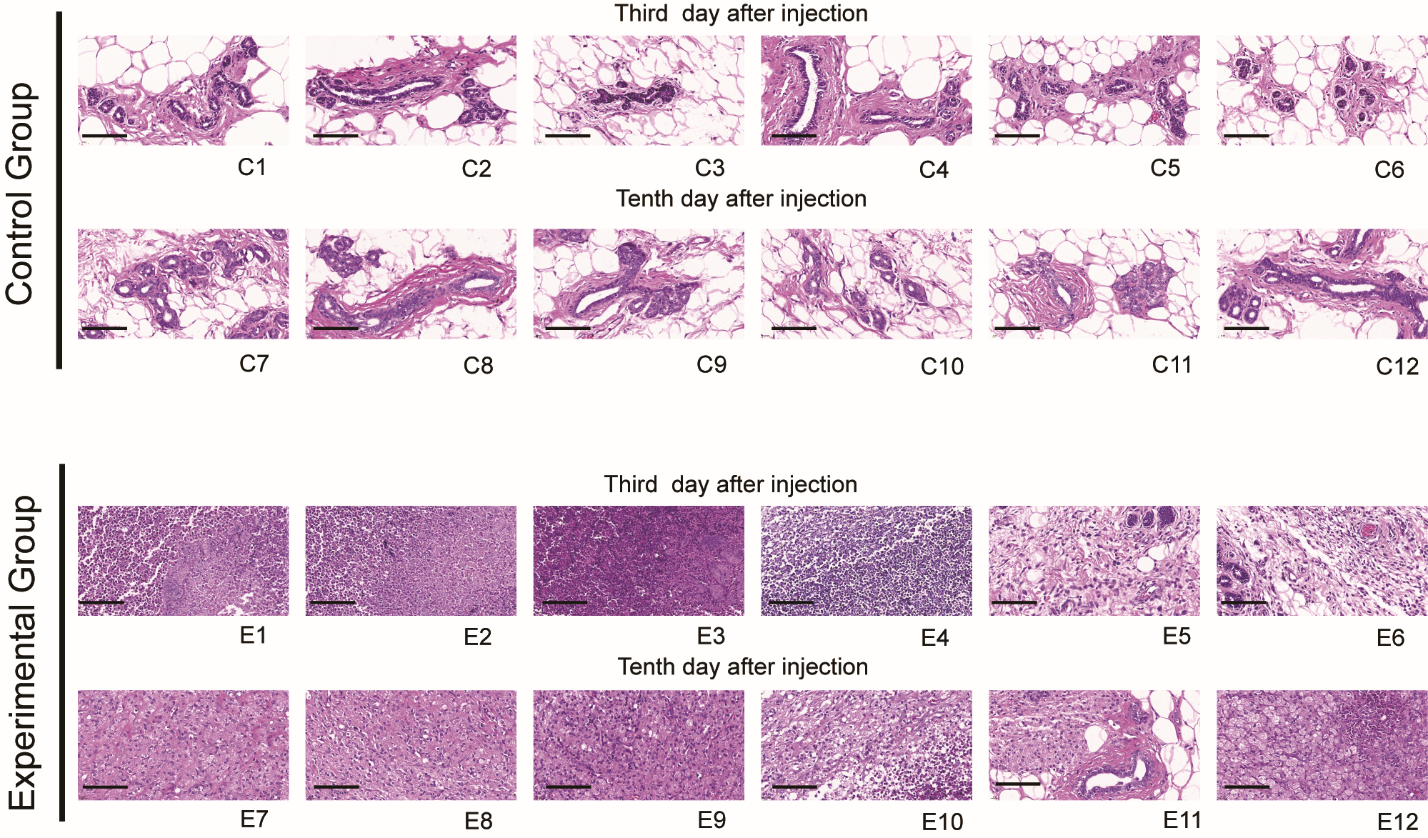


Figure. S2. GLM confirmed by HE staining of mammary tissue in rats.

The experiment including control group (12 rats) and *C. parakroppenstedtii* P1 group (experimental group) (12 rats). After mammary fat pad injection on day 1, six rats in each group and results were observed after sacrificed on day 3 and day 10, respectively. Scale bar represents 100 μm.


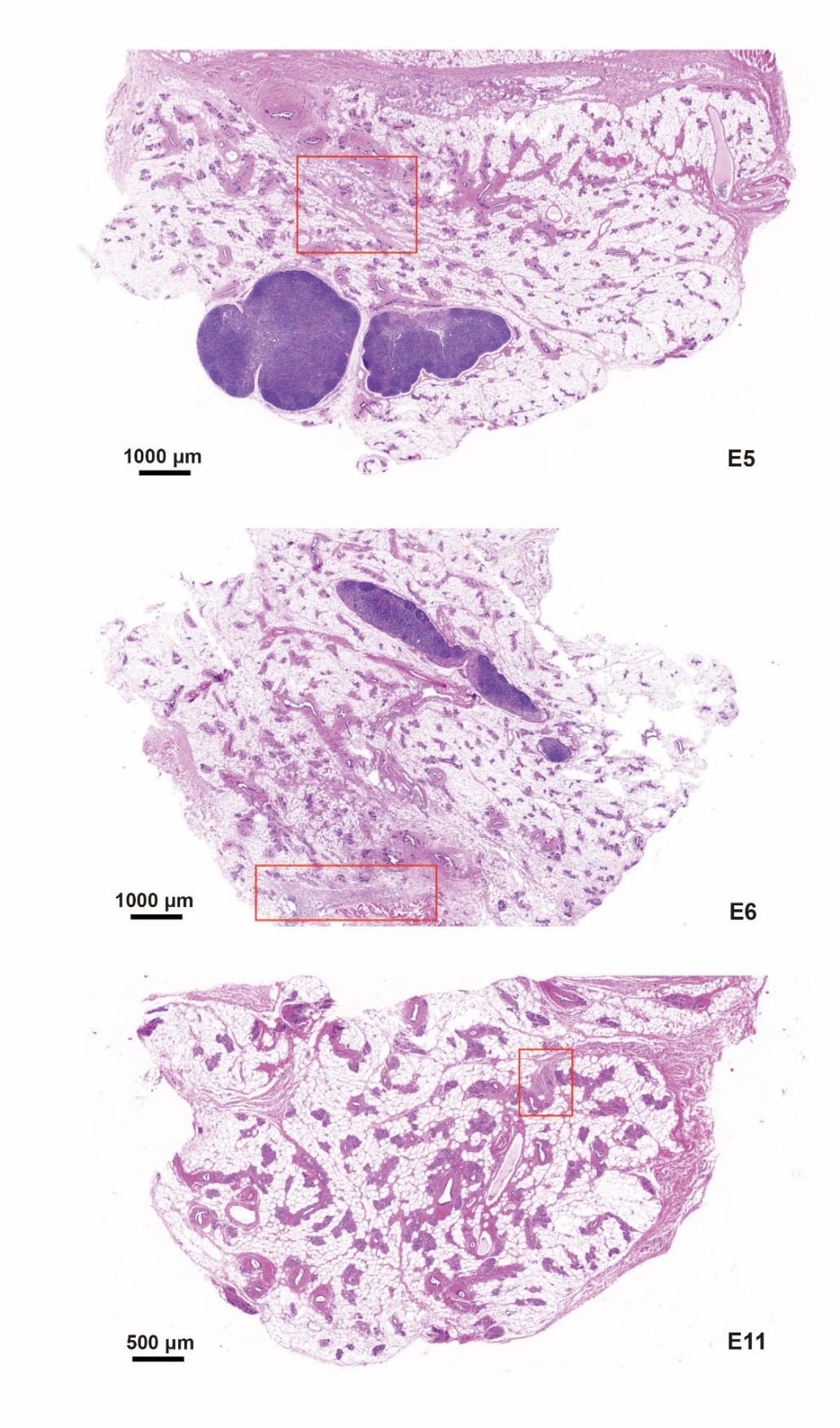


Figure. S3. Histopathological sections of 3 rats with less inflammation.

Two rats (E5 and E6) that sacrifice on day 3 and one rat (E11) that sacrifice on day 10 had a smaller area of inflammation.


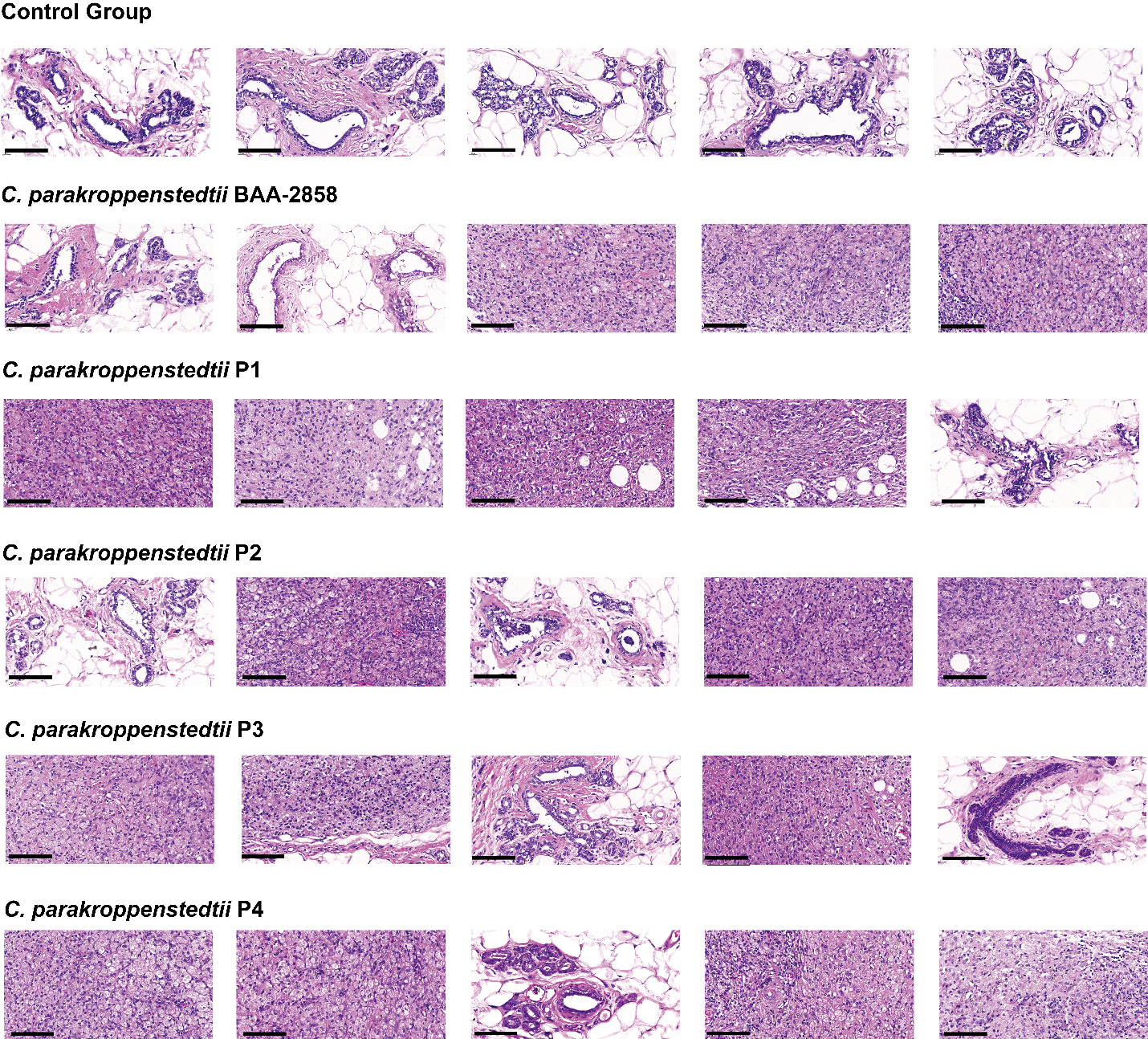


Figure. S4. GLM confirmed by H&E staining of mammary tissue in rats.

The experiment including, control group, *C. parakroppenstedtii* P1 group, *C. parakroppenstedtii* P2 group, *C. parakroppenstedtii* P3 group, *C. parakroppenstedtii* P4 group, and *C. parakroppenstedtii* BAA-2858 group, with five rats in each group. After fat pad injection, results were observed after sacrifice on day 10. Scale bar represents 100 μm.


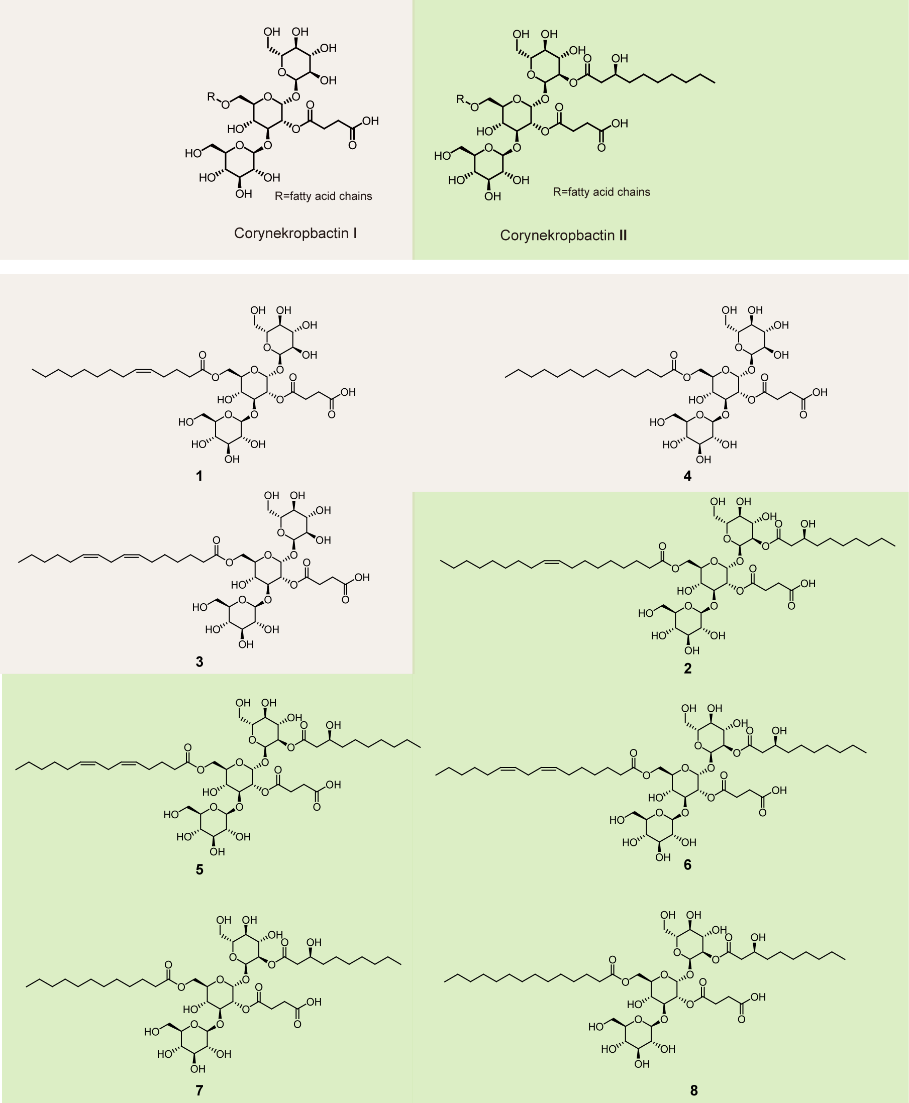


Figure. S5. The structure of corynekropbactins.


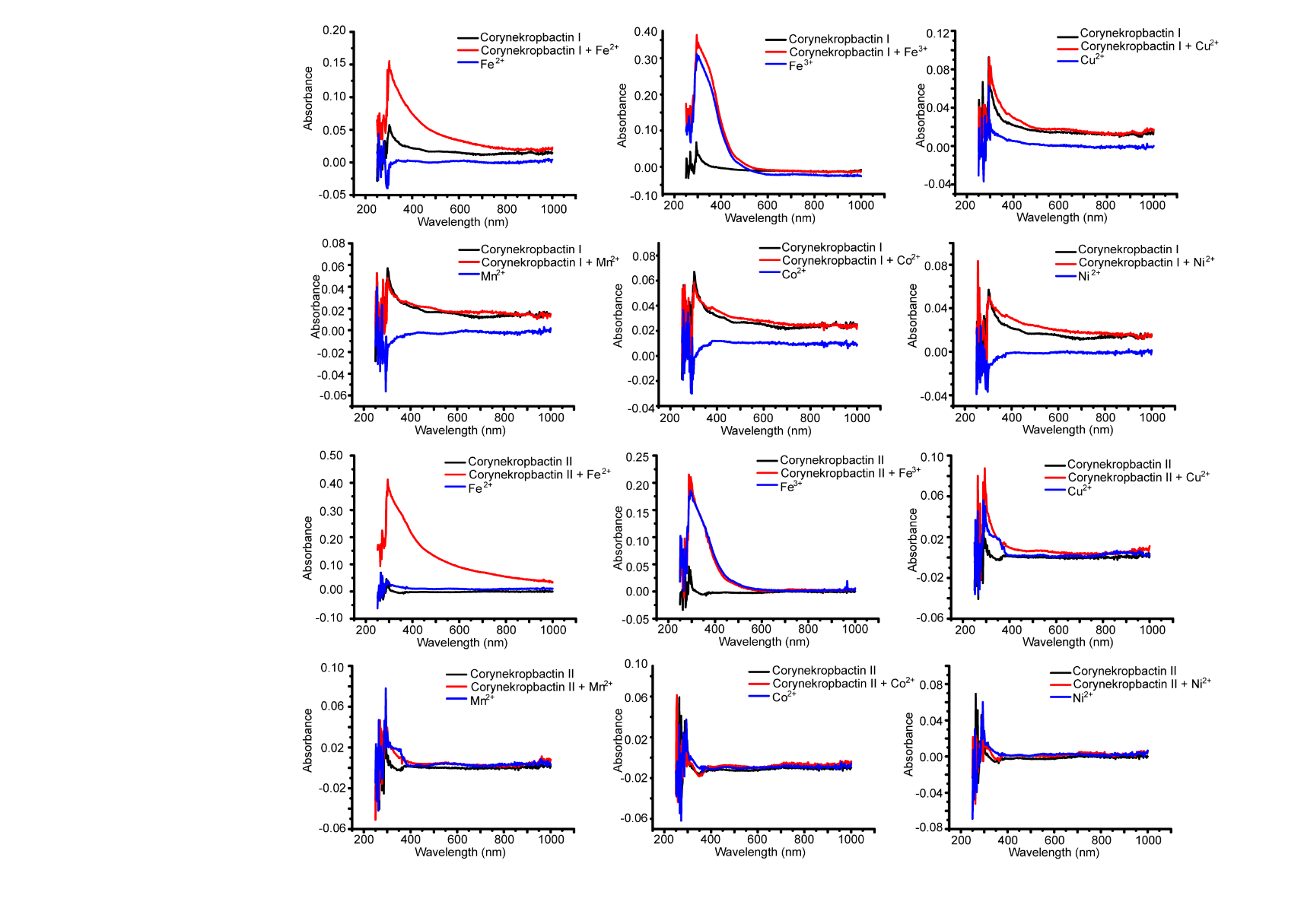


Figure. S6. UV/visible absorption spectra of corynekropbactins and its metal complexes.


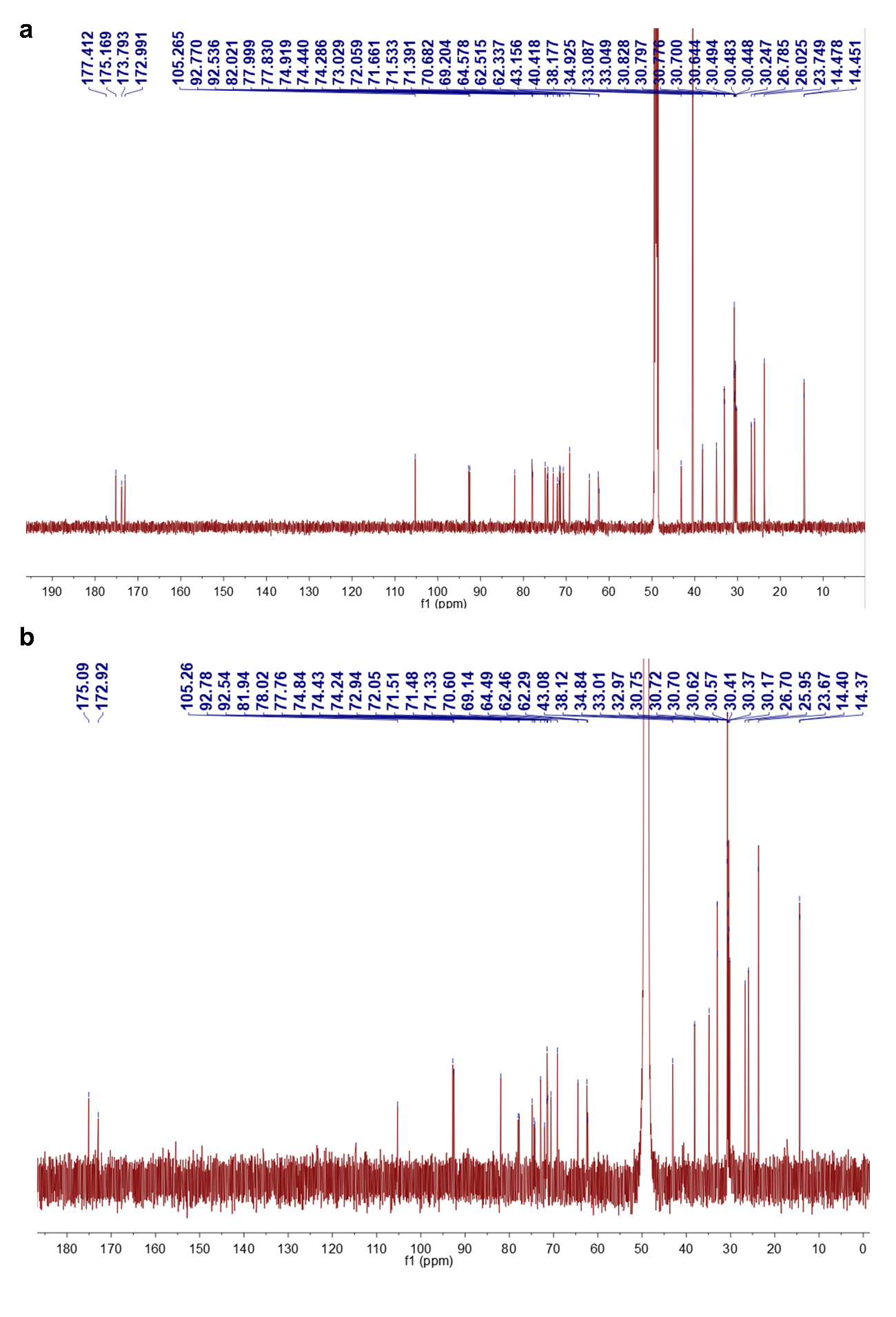

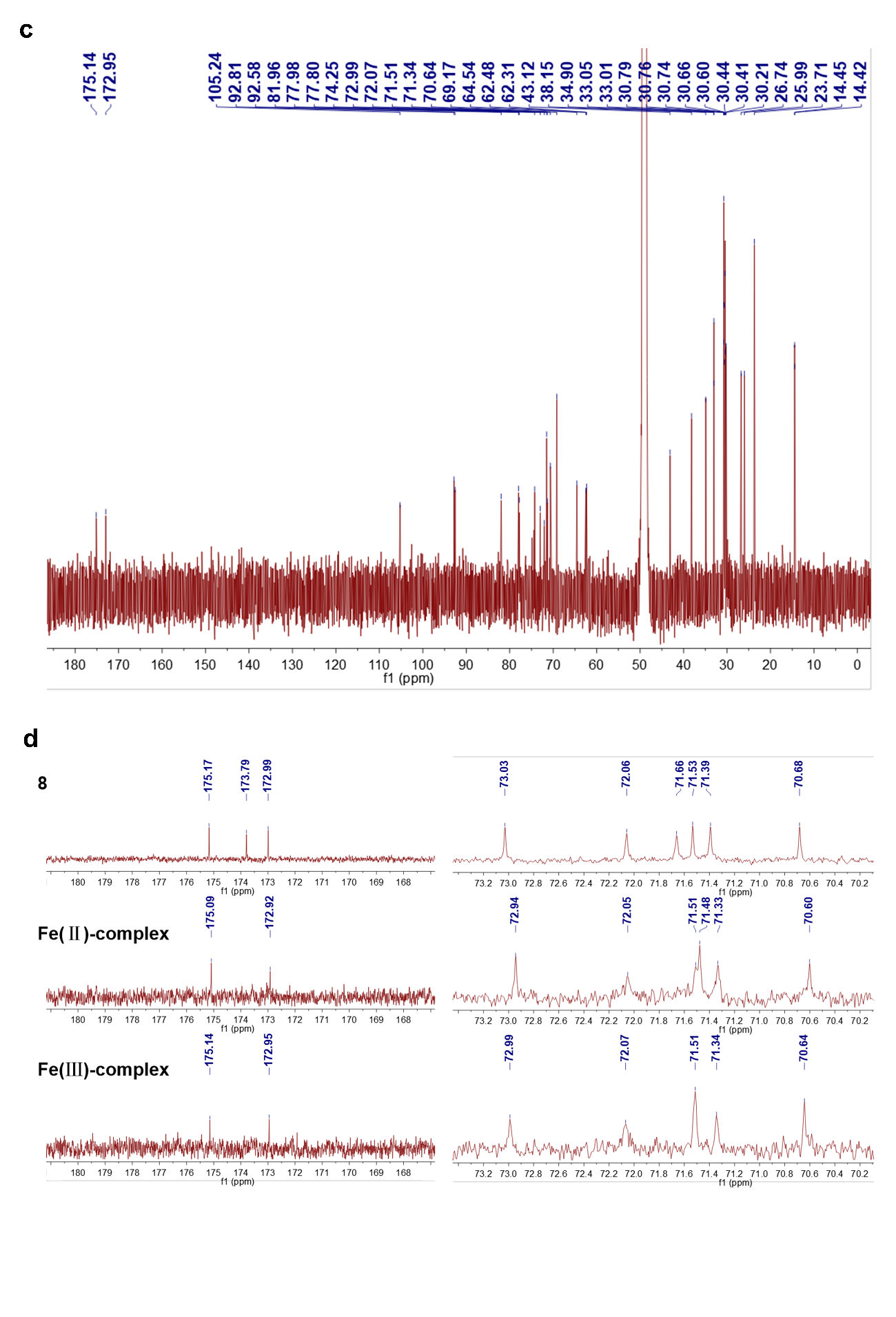


Figure. S7. ^13^C NMR Spectrum of 8, 8-Fe^2+^-complex, and 8-Fe^3+^-complex in CD_3_OD (150 MHz).

(**a**)^13^C NMR Spectrum of **8** in CD_3_OD (150 MHz). (**b**)  ^13^C NMR Spectrum of **8-Fe^2+^-complex** in CD_3_OD (150 MHz). (**c**) ^13^C NMR Spectrum of **8-Fe^3+^-complex** in CD_3_OD (150 MHz). (**d**) ^13^C NMR Spectrum differences of **8**, **8-Fe^2+^-complex**, and **8-Fe^3+^-complex** in CD_3_OD (150 MHz).

**
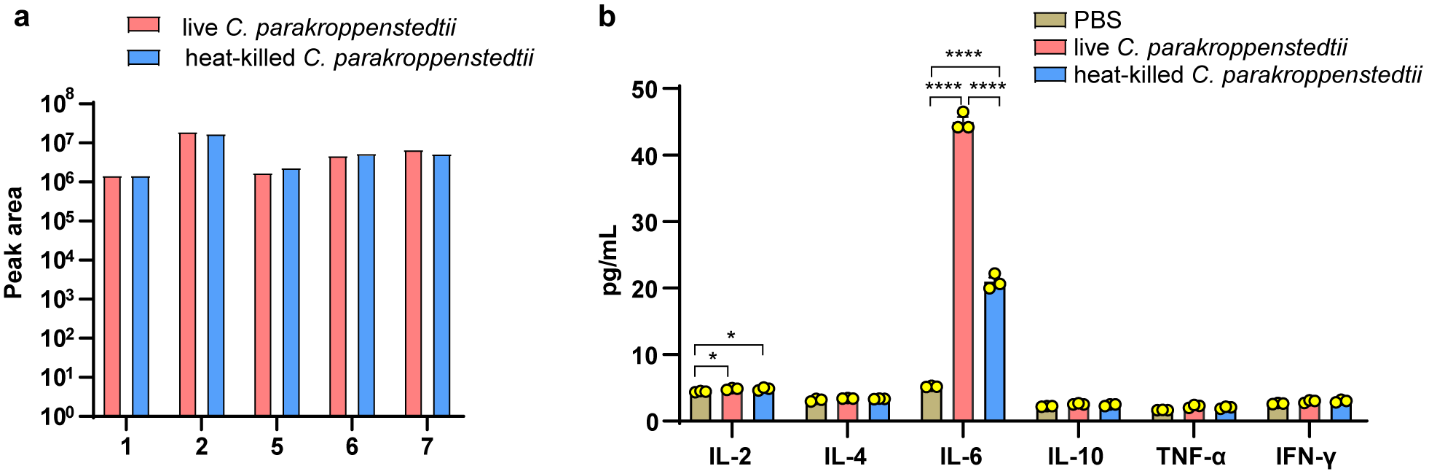
**

Figure. S8. Live or heat killed *C. parakroppenstedtii* changes in cytokine levels in MCF-10A cells.

(**a**). The production of corynekropbactins in live and heat-killed *C. parakroppenstedtii*. The vertical coordinates represent the peak areas extracted of the molecular weights of the corresponding compounds in the LC-MS assay. This batch of samples was prepared and used for the cellular experiments in (**b**). (**b**) Live and heat-killed *C. parakroppenstedtii* cause changes in the levels of cytokines in MCF-10A cells. n=3. Data are presented as mean ± SEM. Group differences were analyzed using one-way ANOVA followed by Tukey's HSD test for post hoc comparisons. *P*-values were adjusted using the Benjamini-Hochberg correction. *p<0.05; **p<0.01; *** p<0.001; **** p<0.0001
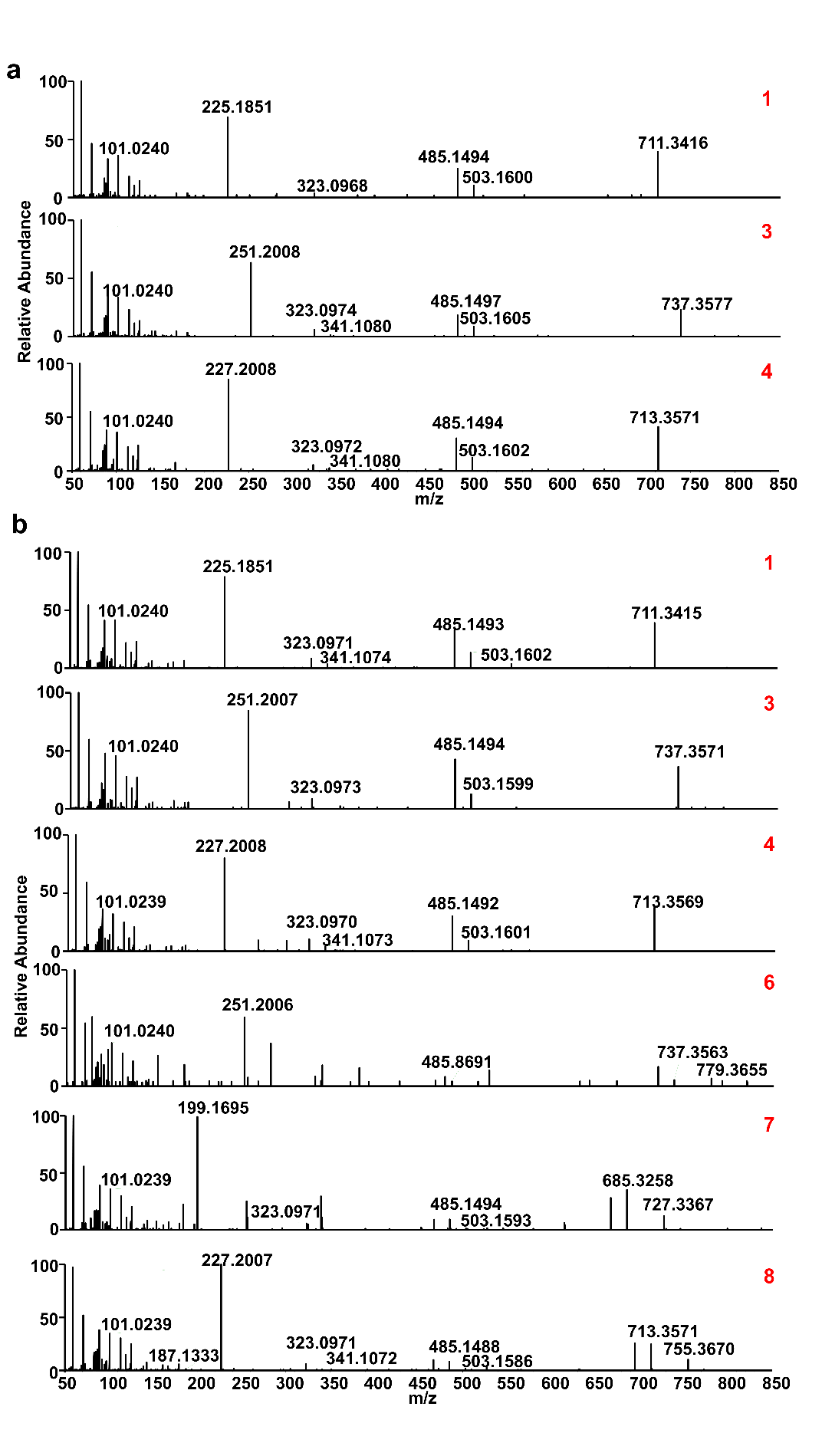


Figure. S9.MS/MS of corynekropbactions.

**(a)**MS/MS of cell supernatants of *C. parakroppenstedtii*-treated MCF-10A. m/z of extracted ions: **1-**811.3582, **3-**837.3735, **4-**813.3735. (**b**) MS/MS of breast extraction of *C. parakroppenstedtii*-treated rats. m/z of extracted ions: **1-**811.3582, **3-**837.3735, **4-**813.3735, **6-**1007.5027, **7-**955.4711, **8**-983.5058.


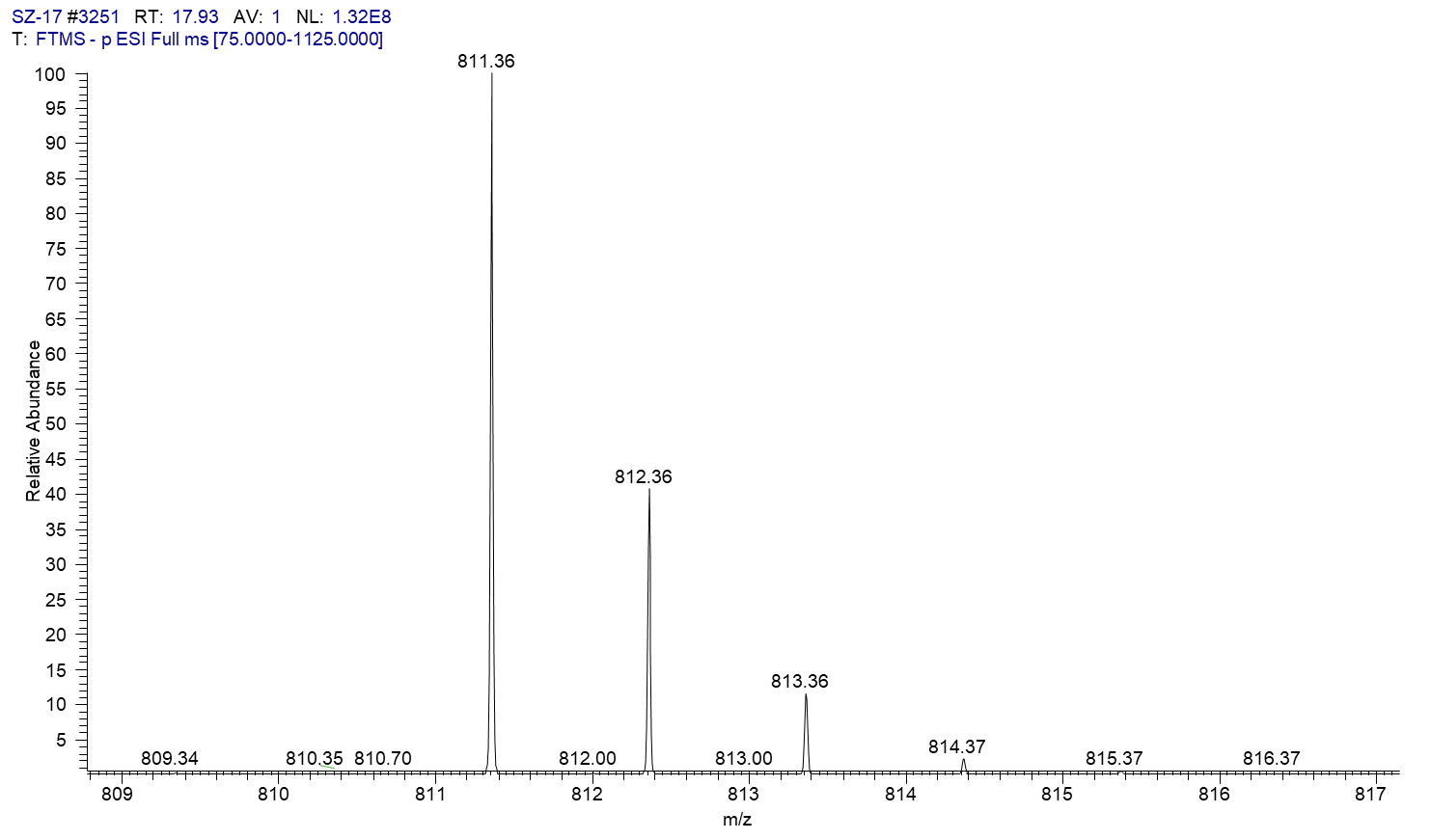


Figure. S10. (-)-HR-ESI-MS Spectrum of 1.


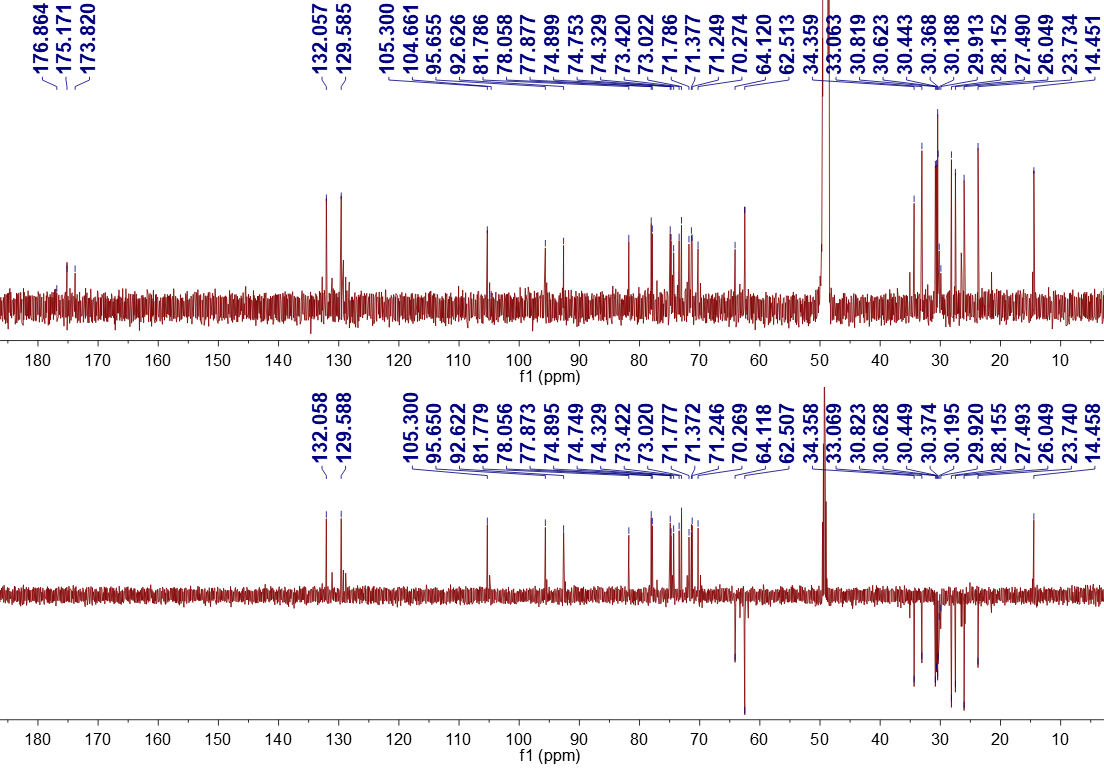


Figure. S11. ^13^C NMR Spectrum of 1 in CD_3_OD (150 MHz).


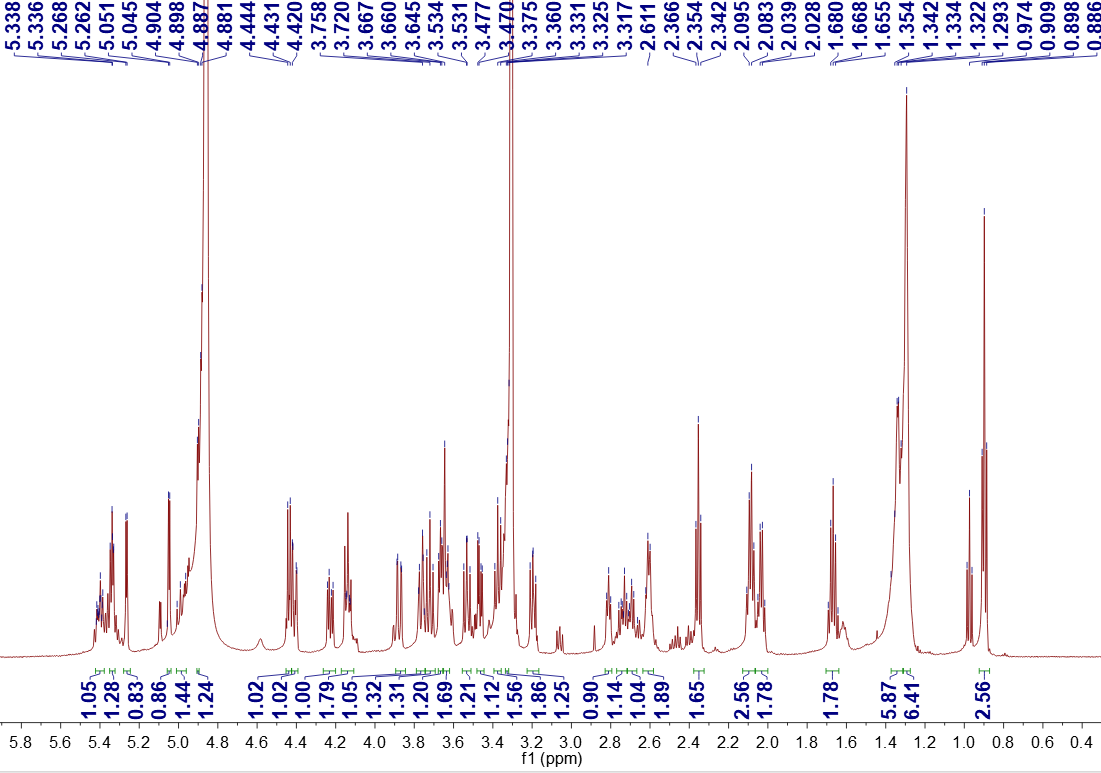


Figure. S12. ^1^H NMR Spectrum of 1 in CD_3_OD (600 MHz).


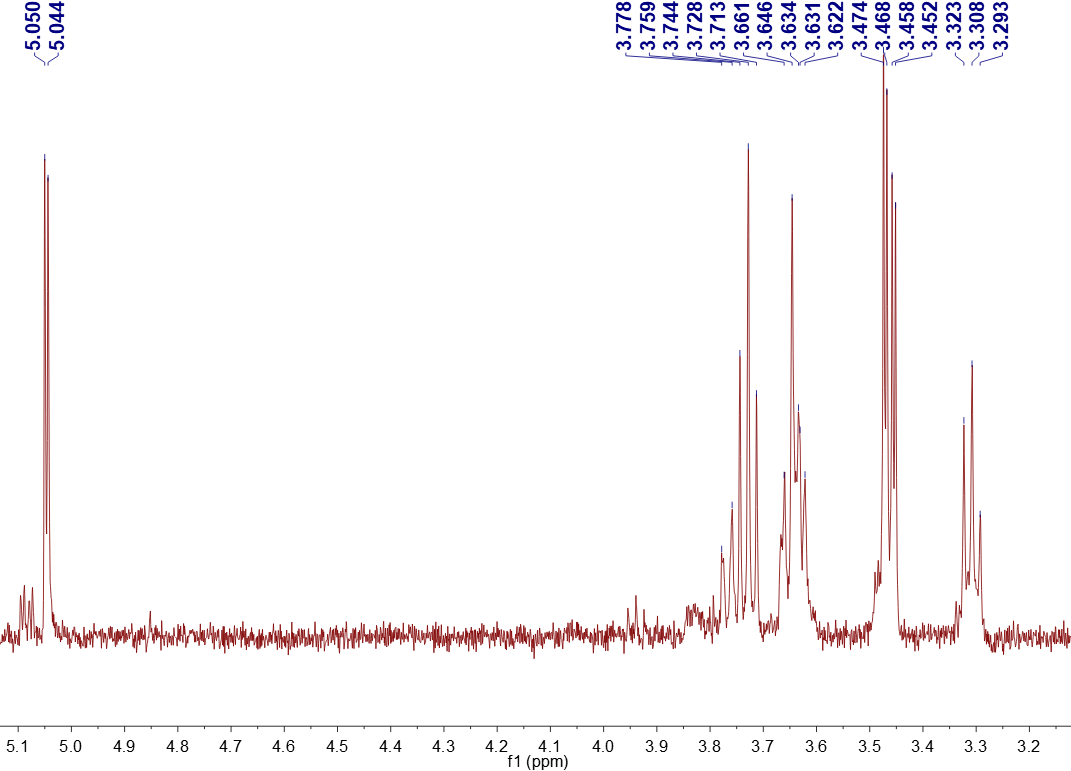


Figure. S13. 1D-TOCSY spectrum at *δ*_H_ 5.05 of 1 in CD_3_OD (600 MHz).


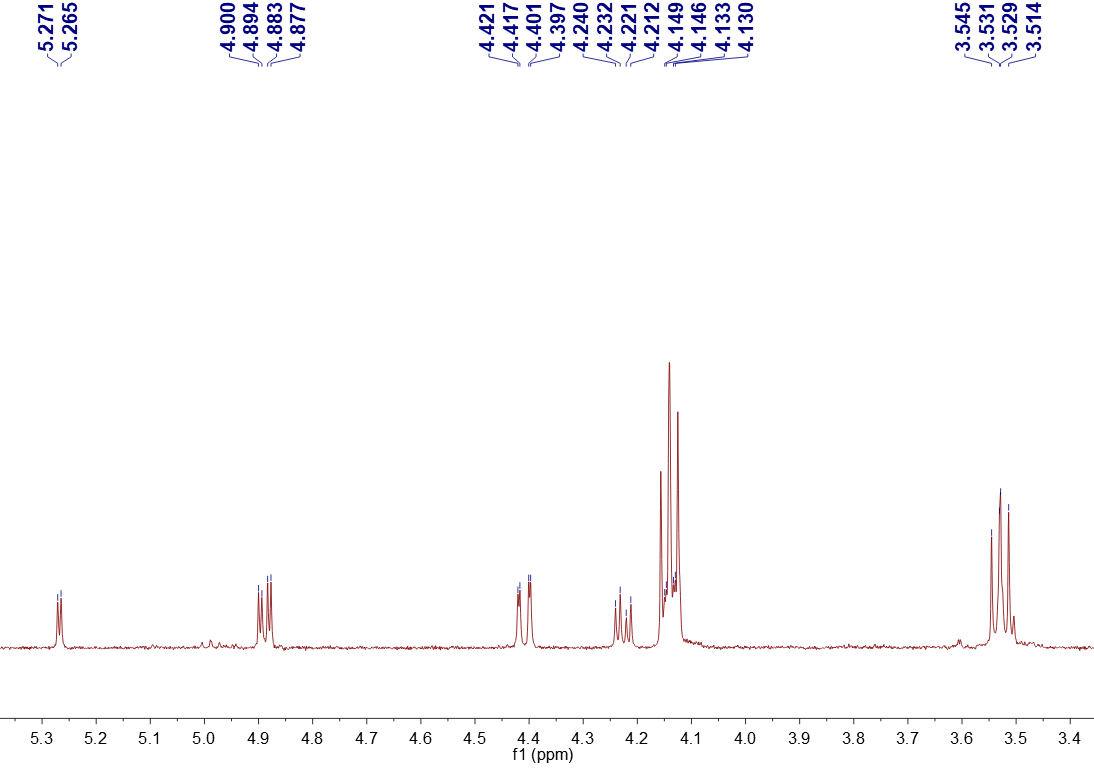


Figure. S14. 1D-TOCSY spectrum at *δ*_H_ 5.26 of 1 in CD_3_OD (600 MHz).


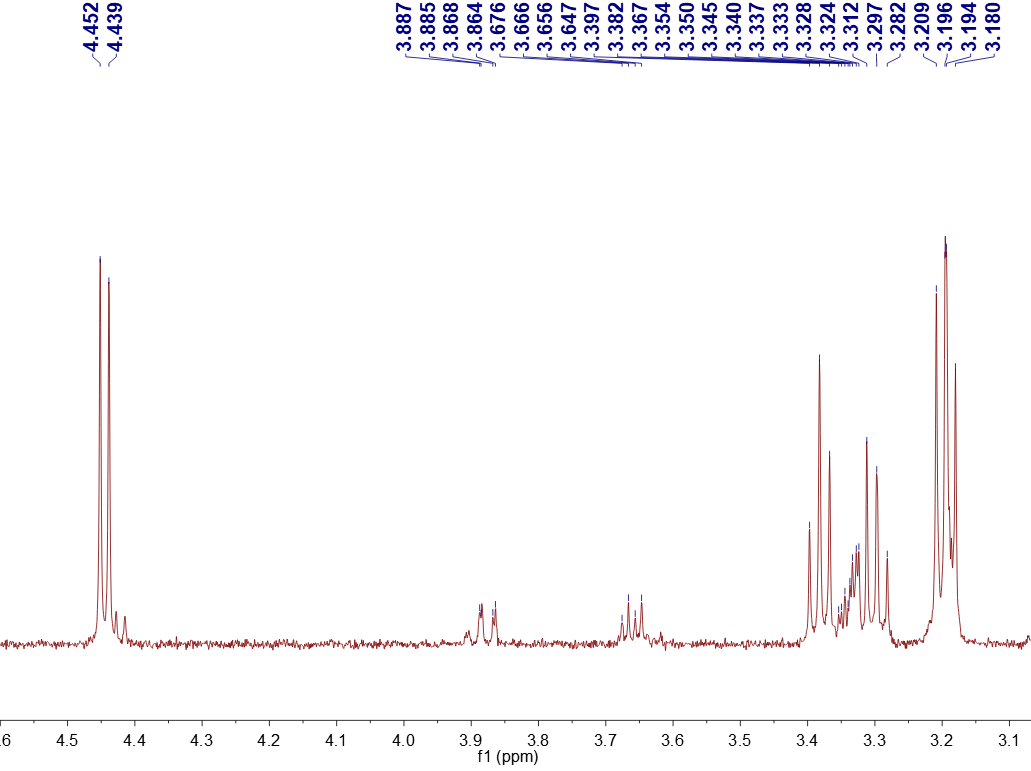


Figure. S15. 1D-TOCSY spectrum at *δ*_H_ 4.45 of 1 in CD_3_OD (600 MHz).


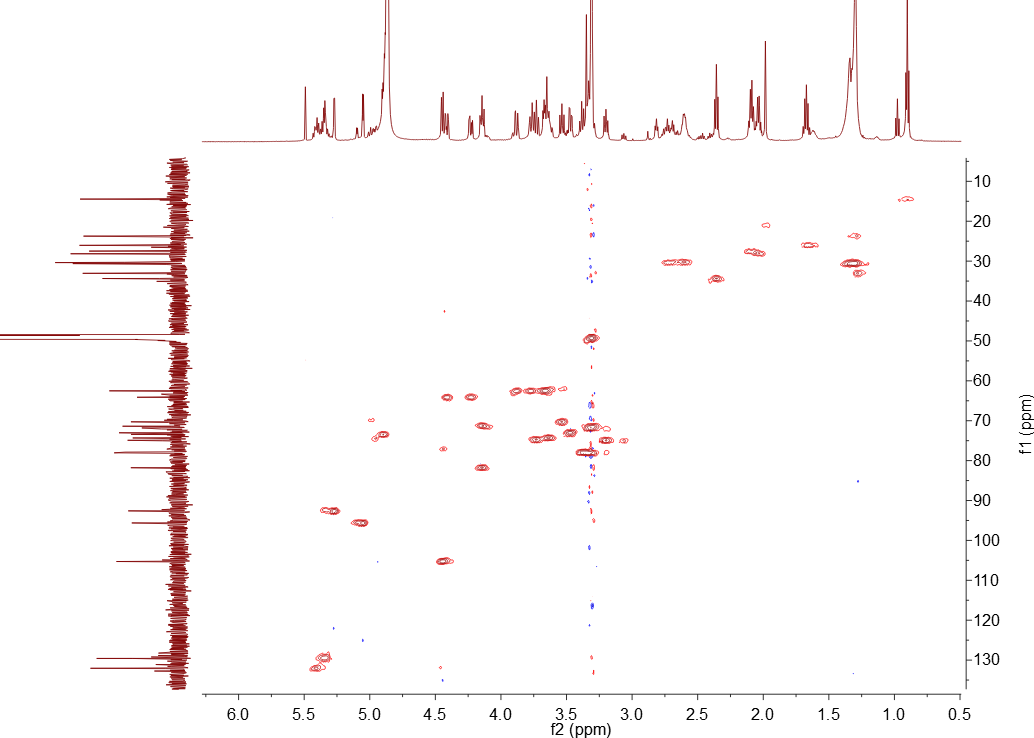


Figure. S16. HSQC Spectrum of 1 in CD_3_OD (150 MHz).


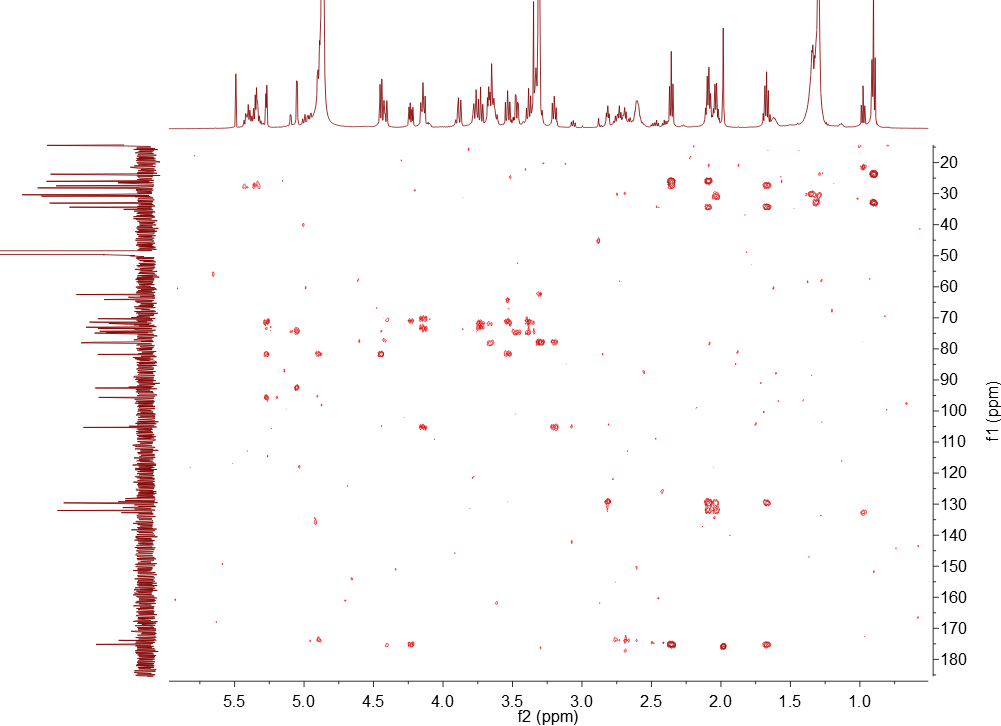


Figure. S17. HMBC Spectrum of 1 in CD_3_OD (150 MHz).


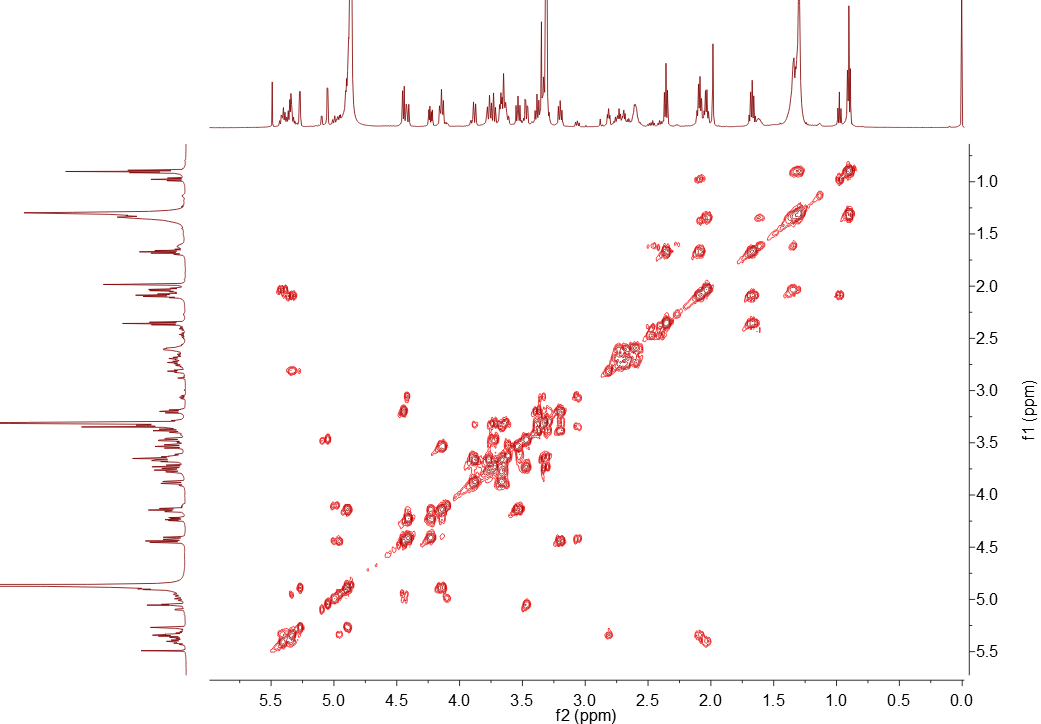


Figure. S18. ^1^H–^1^H COSY Spectrum of 1 in CD_3_OD (150 MHz).


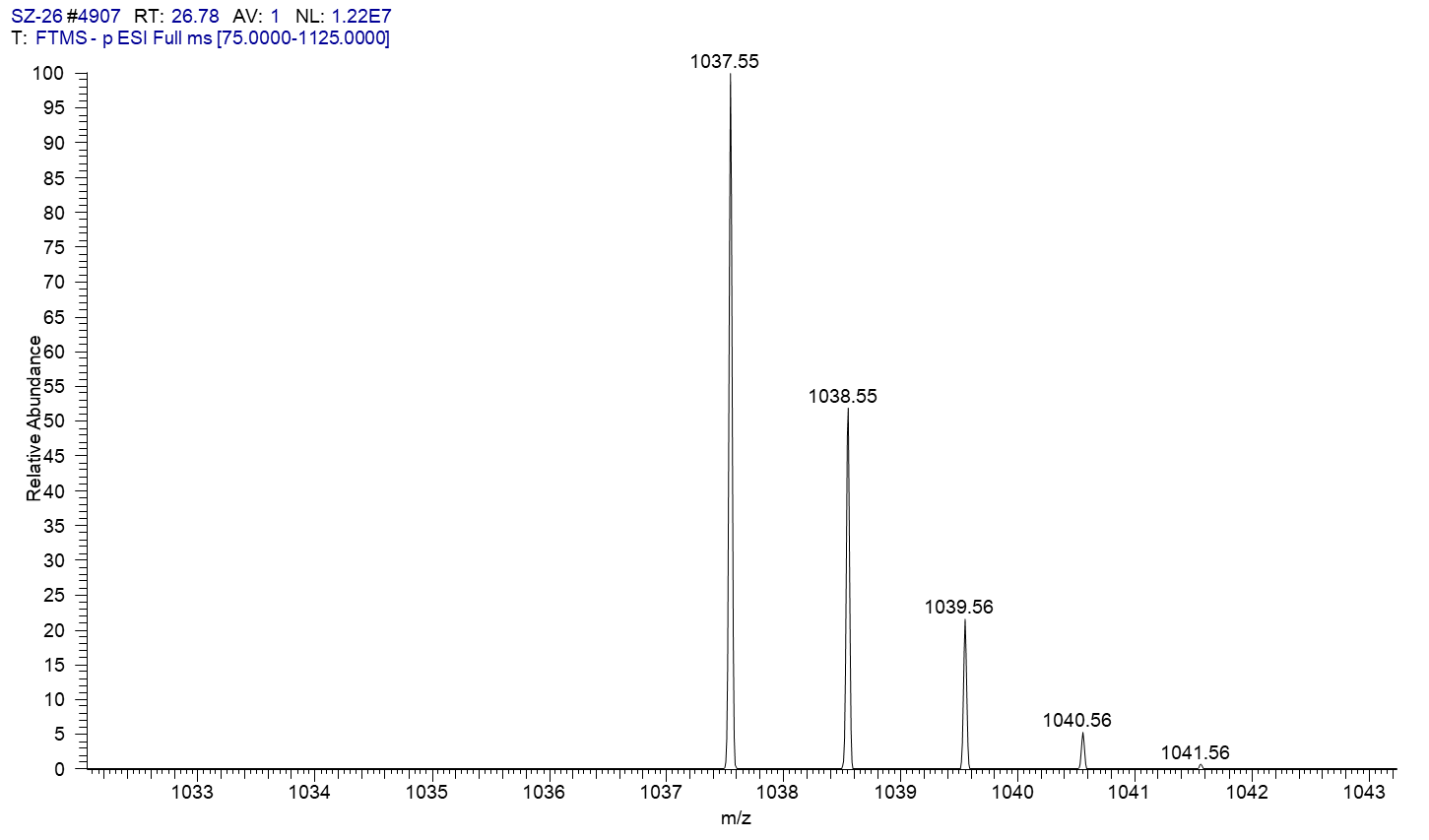


Figure. S19. (-)-HR-ESI-MS Spectrum of 2.


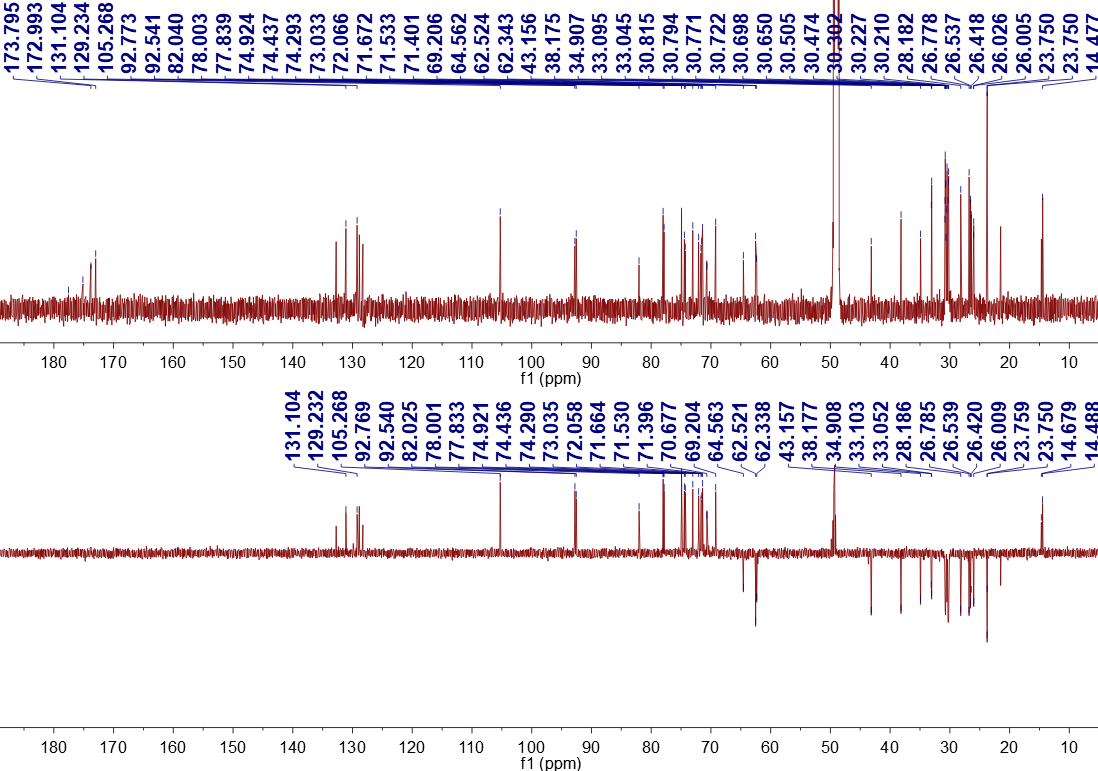


Figure. S20. ^13^C NMR Spectrum of 2 in CD_3_OD (150 MHz).


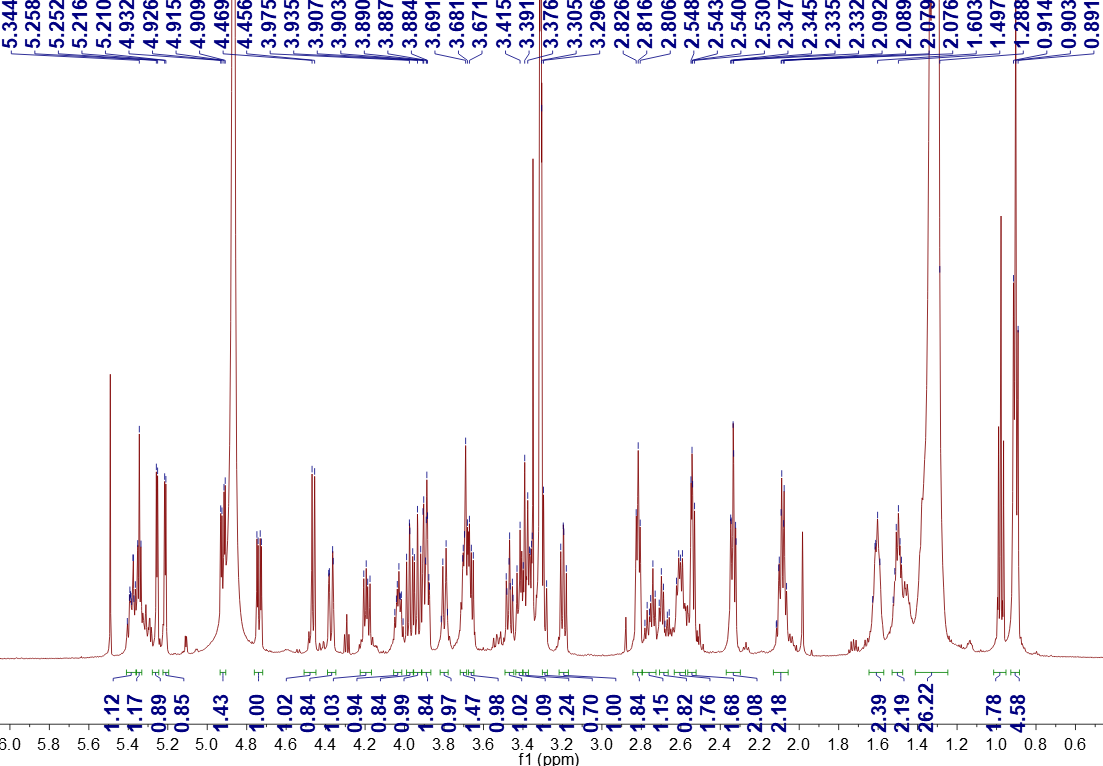


Figure. S21. ^1^H NMR Spectrum of 2 in CD_3_OD (600 MHz).


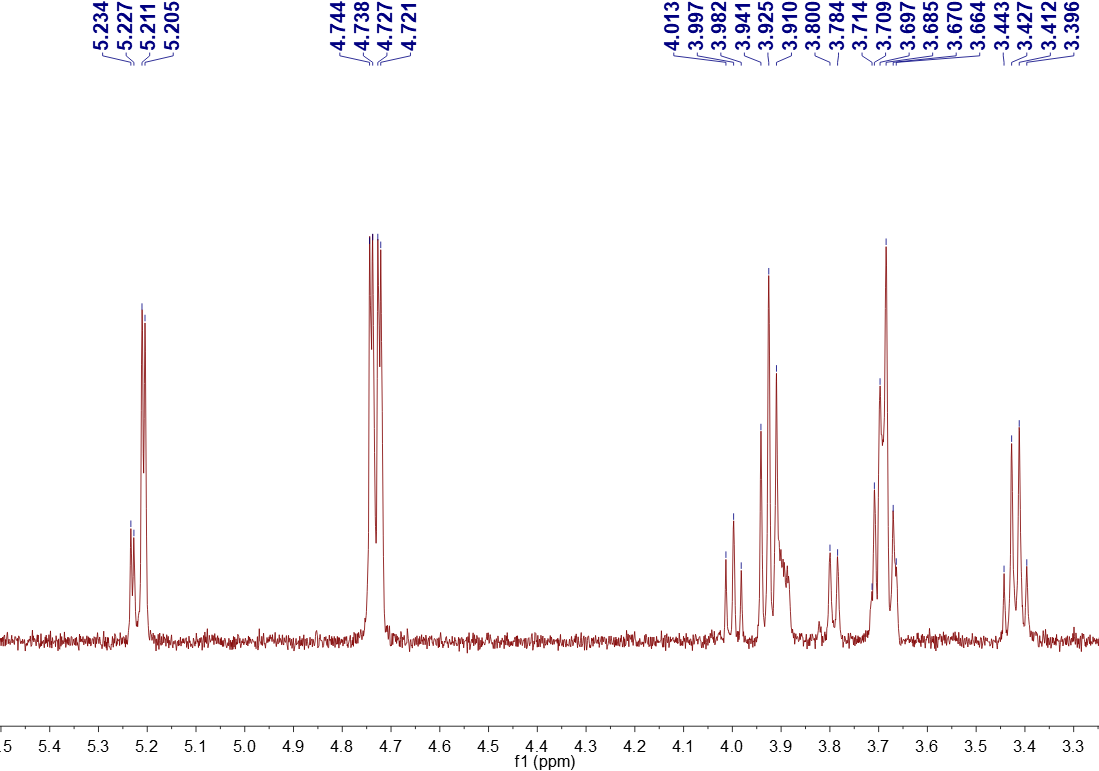


Figure. S22. 1D-TOCSY spectrum at *δ*_H_ 5.21 of 2 in CD_3_OD (600 MHz).


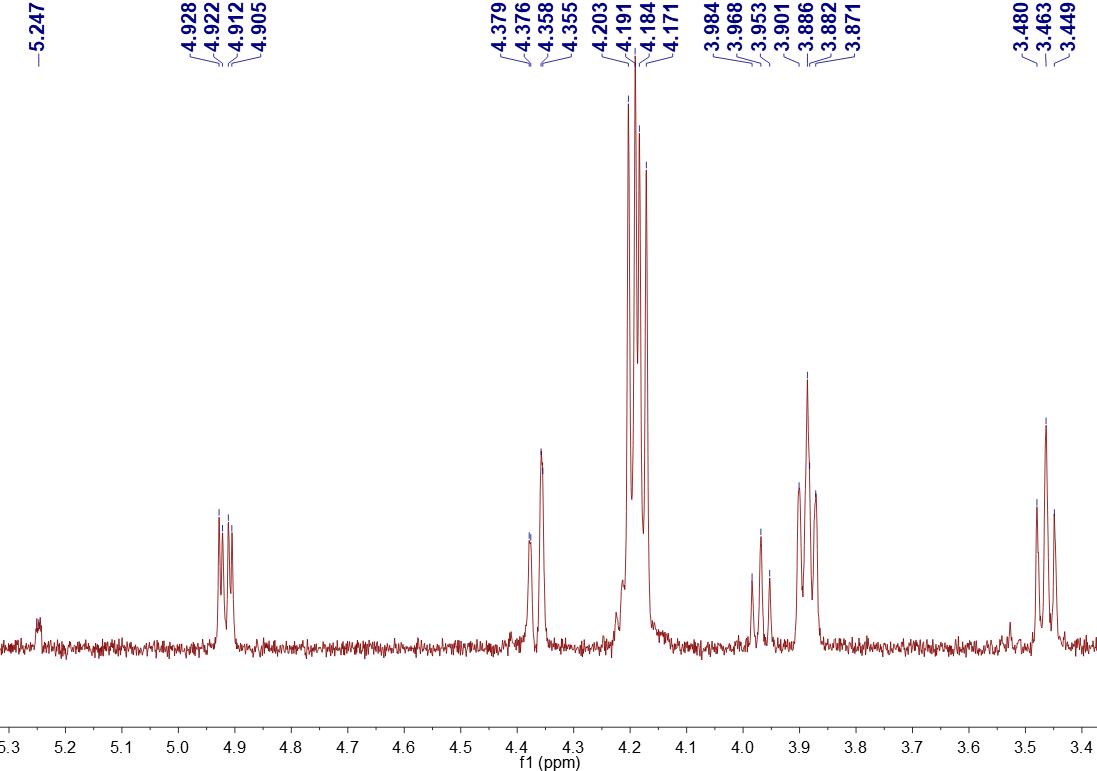


Figure. S23. 1D-TOCSY spectrum at *δ*_H_ 4.20 of 2 in CD_3_OD (600 MHz).


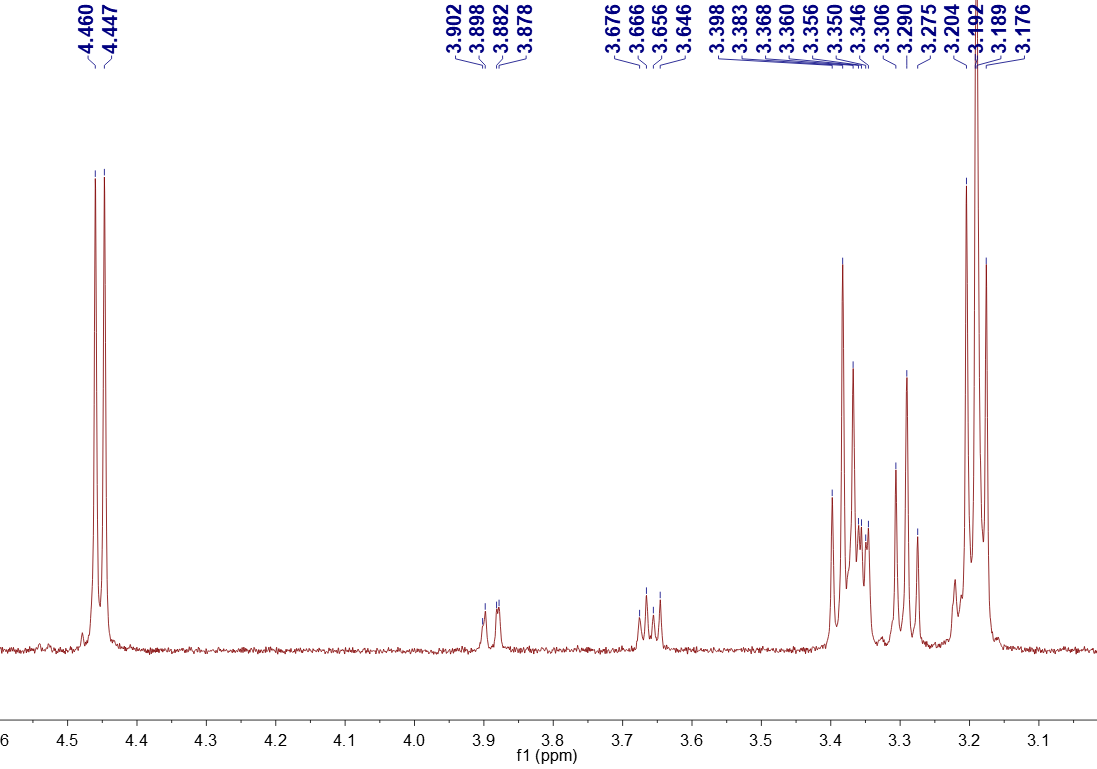


Figure. S24. 1D-TOCSY spectrum at *δ*_H_ 4.45 of 2 in CD_3_OD (600 MHz).


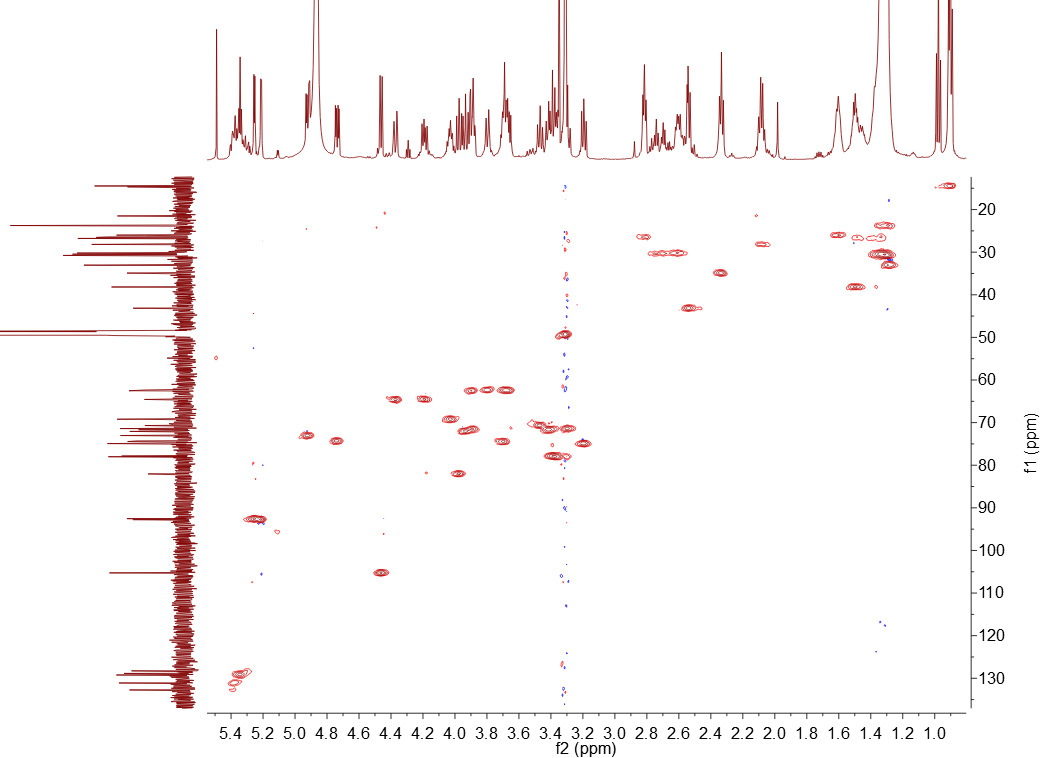


Figure. S25. HSQC Spectrum of 2 in CD_3_OD (150 MHz).


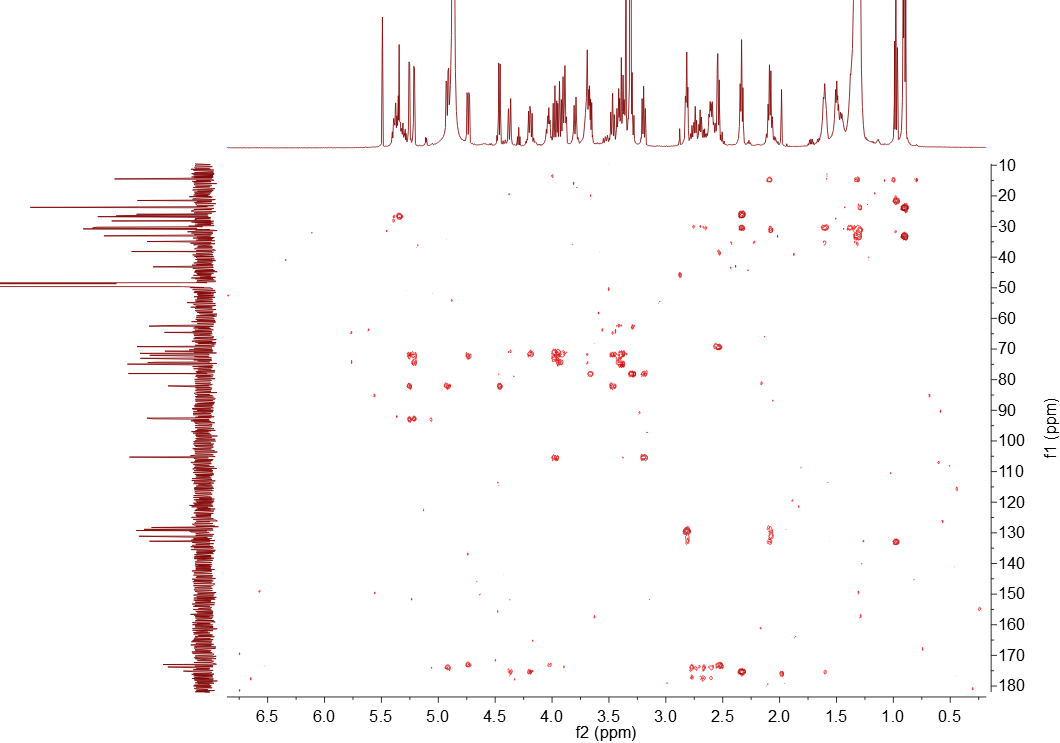


Figure. S26. HMBC Spectrum of 2 in CD_3_OD (150 MHz).


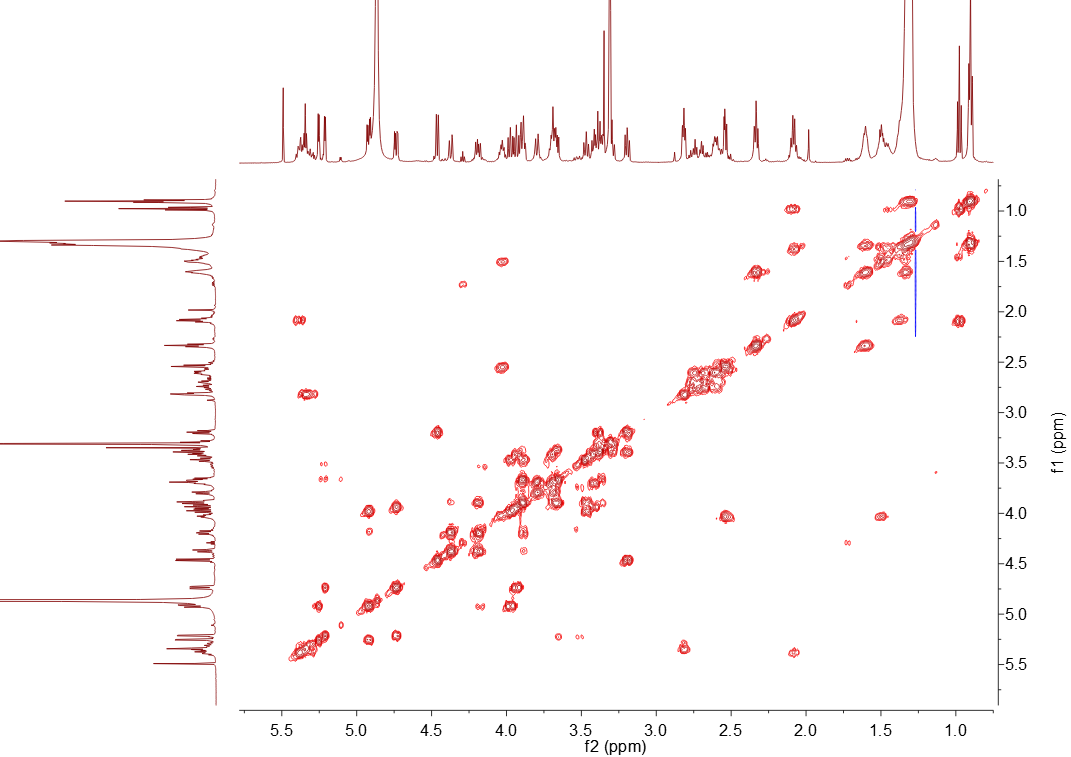


Figure. S27. ^1^H–^1^H COSY Spectrum of 2 in CD_3_OD (150 MHz).


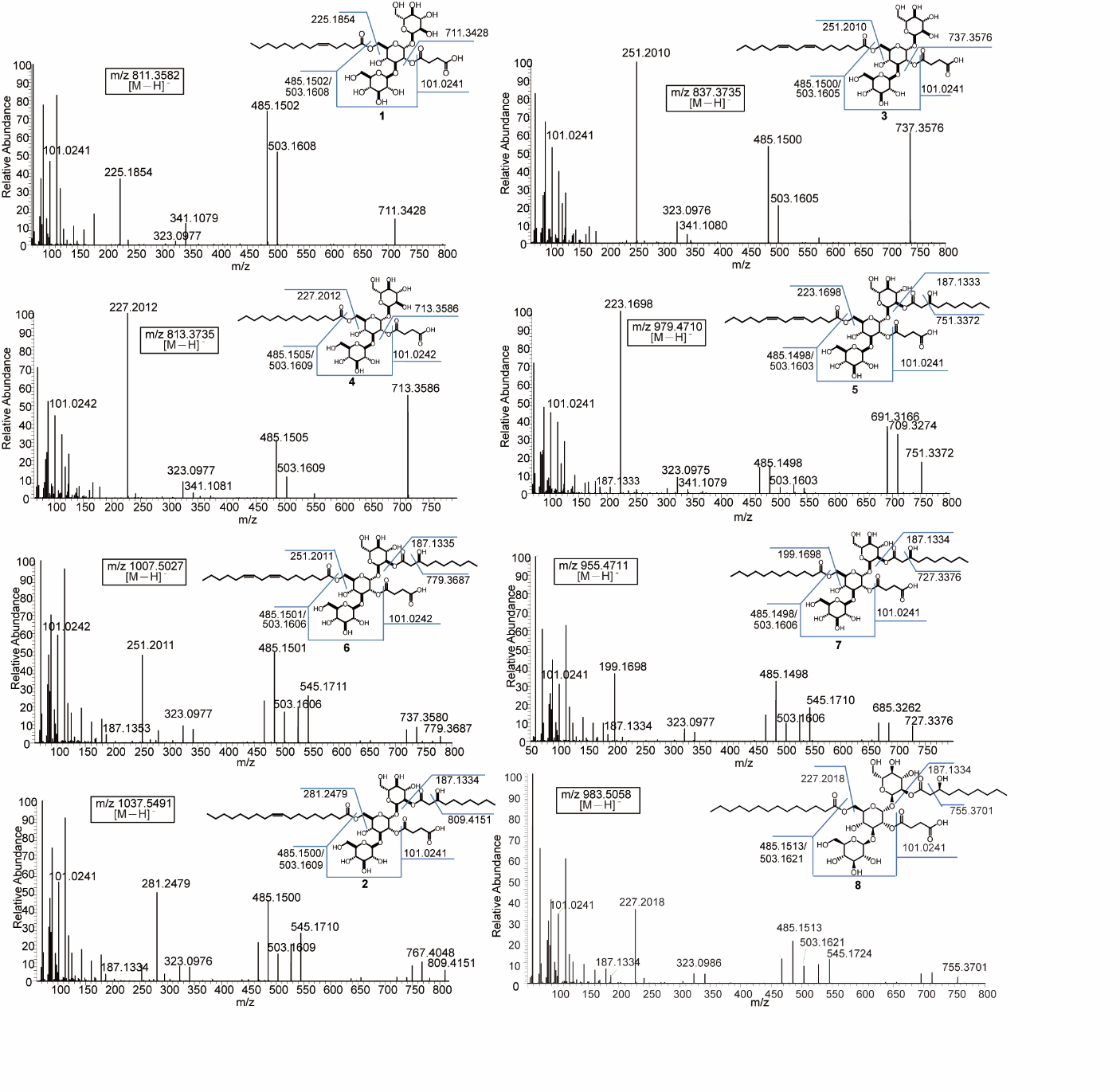


Figure. S28. MS/MS of corynekropbactins (1-8).

Table S1. The information of the included patients with GLM were preformed 16s rRNA sequencing.

| Patient ID | Sample ID in 16s rRNA metagenome Sequencing* | Age | Clinical routine culture | *C. kroppenstedtii* result by sequencing |
| --- | --- | --- | --- | --- |
| GP1 | S90.A.1.H.1 | 35 | Negative | No |
| GP2 | S68.A.1.H.1 | 40 | Negative | No |
| GP3 | S58.A.1.H.1 | 32 | N.A. | Yes |
| GP4 | S40.A.1.H.1 | 26 | Negative | Yes |
| GP5 | S29.A.1.H.1 | 33 | Negative | No |
| GP6 | S48.A.1.H.1 | 30 | Negative | No |
| GP7# | S53.A.1.H.1 | 48 | Negative | Yes |
| GP7# | S52.A.1.H.1 | 49 | Negative | Yes |
| GP8 | S84.A.1.H.1 | 25 | Negative | Yes |
| GP9 | S83.A.1.H.1 | 28 | Negative | Yes |
| GP10 | S44.A.1.H.1 | 33 | N.A. | No |
| GP11 | S28.A.1.H.1 | 39 | Negative | Yes |
| GP12 | S77.A.1.H.1 | 26 | *C. kroppenstedtii* | Yes |
| GP13 | S47.A.1.H.1 | 45 | N.A. | Yes |
| GP14 | S73.A.1.H.1 | 32 | Negative | Yes |
| GP15 | S97.A.1.H.1 | 55 | *Enterobacter sakazakii* | Yes |
| GP16 | S19.A.1.H.1 | 32 | Negative | Yes |
| GP17 | S43.A.1.H.1 | 38 | Negative | Yes |
| GP18 | S87.A.1.H.1 | 26 | Negative | No |
| GP19 | S75.A.1.H.1 | 22 | Negative | Yes |
| GP20 | S32.A.1.H.1 | 32 | Negative | No |
| GP21 | S49.A.1.H.1 | 26 | Negative | Yes |
| GP22 | S33.A.1.H.1 | 28 | N.A. | Yes |
| GP23 | S16.A.1.H.1 | 32 | Negative | Yes |
| GP24 | S34.A.1.H.1 | 38 | N.A. | No |
| GP25 | S108.A.1.H.1 | 31 | Negative | No |
| GP26 | S65.A.1.H.1 | 43 | Negative | No |
| GP27 | S107.A.1.H.1 | 29 | Negative | Yes |
| GP28 | S106.A.1.H.1 | 33 | Negative | No |
| GP29† | S114.A.2.H.1 | 35 | Negative | No |
| GP29† | S114.A.1.H | 35 | Negative | Yes |
| GP30 | S13.A.1.H | 29 | Negative | Yes |
| GP31 | S45.A.1.H | 40 | Negative | Yes |
| GP32 | S7.A.1.H | 25 | Negative | Yes |
| GP33 | S86.A.1.H | 27 | N.A. | Yes |
| GP34 | S63.A.1.H | 32 | N.A. | Yes |
| GP35 | S56.A.1.H | 30 | Negative | Yes |
| GP36 | S78.A.1.H | 33 | *C. kroppenstedtii* | Yes |
| GP37 | S3.A.1.H | 31 | Negative | Yes |
| GP38 | S6.A.1.H | 26 | Negative | Yes |
| GP39‡ | S103.A.1.H | 32 | *Lactobacillus sharpeae* | Yes |
| GP39‡ | S57.A.1.H | 32 | *Candida albicans* | Yes |
| GP40 | S70.A.1.H | 31 | Negative | Yes |
| GP41 | S36.A.1.H | 33 | Negative | Yes |
| GP42 | S42.A.1.H | 34 | Negative | Yes |
| GP43 | S51.A.1.H | 30 | N.A. | Yes |
| GP44 | S67.A.1.H | 30 | N.A. | Yes |
| GP45 | S41.A.1.H | 40 | N.A. | Yes |
| GP46 | S80.A.1.H | 33 | N.A. | Yes |
| GP47 | S96.A.1.H | 33 | *Actinomyces odontolyticus* | Yes |
| GP48 | S95.A.1.H | 36 | Negative | Yes |
| GP49 | S109.A.1.H | 35 | Negative | Yes |
| GP50 | S21.A.1.H | 34 | *Staphylococcus warneri* | Yes |
| GP51 | S111.A.1.H | 30 | Negative | Yes |
| GP52 | S112.A.1.H | 31 | Negative | Yes |
| GP53 | S31.A.1.H | 41 | Negative | Yes |
| GP54 | S92.A.1.H | 36 | Negative | Yes |
| GP55 | S113.A.1.H | 38 | Negative | Yes |
| GP56 | S15.A.1.H | 26 | Negative | Yes |
| GP57 | S115.A.1.H | 36 | Negative | Yes |
| GP58 | S118.A.1.H | 45 | Negative | Yes |
| GP59 | S119.A.1.H | 27 | N.A. | Yes |

#Patient GP7 was hospital admission twice, bilateral breast tissue was sampled

†Patient GP29 was hospital admission once, but bilateral breast tissue was sampled.

‡Patient GP39 was hospital admission twice, bilateral breast tissue was sampled

* Except for the three patients (GP7, 29, and 39), all other samples were from primary patients with breast symptoms, who came to our hospital for diagnosis without treatment.

N.A.: Not application.

Table S2. The information of the patients with GLM with both breast tissue and pus were preformed 16s rRNA sequencing

| Patient ID | Sample name of breast tissue | Sample name of pus |
| --- | --- | --- |
| GP3 | S58.A.1.H.1 | S58_C_1_K_1 |
| GP7 | S52.A.1.H.1 | S52_C_1_K_1 |
| GP9 | S83.A.1.H.1 | S83_C_1_K |
| GP13 | S47.A.1.H.1 | S47_C_1_K |
| GP16 | S19.A.1.H.1 | S19_C_1_K_1 |
| GP21 | S49.A.1.H.1 | S49_C_1_K_1 |
| GP26 | S65.A.1.H.1 | S65_C_1_K_1 |
| GP32 | S7.A.1.H | S7_C_1_K |
| GP38 | S6.A.1.H | S6_C_1_K |
| GP40 | S70.A.1.H | S70_C_1_K |
| GP53 | S31.A.1.H | S31_C_1_K |

Table S3. Th1/Th2-type cytokine levels from patients with GLM.

| Patients ID | Age | IL-2  (pg/mL) | IL-4  (pg/mL) | IL-5 (pg/mL) | IL-6 (pg/mL) | IL-10 (pg/mL) | TNF-α (pg/mL) | IFN-γ (pg/mL) |
| --- | --- | --- | --- | --- | --- | --- | --- | --- |
| GP60 | 29 | 7.94 | 6.50 | 2.38 | 264.72 | 5.72 | 3.11 | 2.72 |
| GP61 | 33 | 6.60 | 4.77 | 2.29 | 24.87 | 4.93 | 3.21 | 4.05 |
| GP62 | 29 | 6.48 | 5.41 | 2.26 | 18.75 | 5.54 | 2.56 | 5.61 |
| GP63 | 33 | 5.28 | 4.25 | 2.33 | 3.72 | 3.39 | 2.72 | 3.00 |
| GP64 | 31 | 4.65 | 3.63 | 2.38 | 22.87 | 2.96 | 4.20 | 5.95 |
| GP65 | 38 | 4.91 | 3.82 | 2.25 | 22.65 | 3.27 | 3.25 | 3.88 |
| GP66 | 39 | 3.40 | 3.31 | 2.23 | 54.34 | 4.35 | 3.88 | 2.56 |
| GP67 | 35 | 8.46 | 6.24 | 2.29 | 35.31 | 3.97 | 3.02 | 2.46 |
| GP68 | 35 | 6.83 | 4.98 | 2.16 | 5.85 | 4.53 | 2.47 | 3.22 |
| GP69 | 31 | 6.53 | 4.98 | 2.29 | 7.33 | 5.76 | 3.85 | 5.05 |
| GP70 | 34 | 5.95 | 4.93 | 2.24 | 5.05 | 4.06 | 2.67 | 3.72 |
| GP71 | 39 | 4.23 | 2.84 | 2.35 | 25.40 | 3.75 | 5.19 | 3.11 |
| GP72 | 28 | 5.84 | 6.20 | 2.26 | 7.16 | 5.32 | 4.99 | 2.74 |
| Normal reference ranges* | | ≤11.4 | ≤12.9 | ≤20 | ≤20.0 | ≤5.9 | ≤5.5 | ≤18 |

*Normal reference range is according to the instrument of kit.

Table S4. The information of the included patients in H&E staining and immunofluorescence staining

| Patient ID | Age | Pathologic Diagnosis | Culture | Lesion Size | History Of Chronic Illness |
| --- | --- | --- | --- | --- | --- |
| GP60 | 29 | GLM | Negative | N.A. | No |
| GP63 | 33 | GLM | Negative | N.A. | No |
| GP64 | 31 | GLM | Negative | N.A. | No |
| GP73 | 28 | GLM | Negative | N.A. | No |
| GP74 | 30 | GLM | Negative | N.A. | No |
| GP75 | 29 | GLM | Negative | N.A. | No |
| FP1 | 32 | fibroadenoma | N.A. | < 2cm | No |
| FP2 | 32 | fibroadenoma | N.A. | < 2cm | No |
| FP3 | 32 | fibroadenoma | N.A. | < 2cm | No |
| FP4 | 32 | fibroadenoma | N.A. | < 2cm | No |
| FP5 | 32 | fibroadenoma | N.A. | < 2cm | No |
| FP6 | 32 | fibroadenoma | N.A. | < 2cm | No |
| IP1 | 33 | invasive ductal carcinoma | N.A. | ≤ 3cm | No |
| IP2 | 36 | invasive ductal carcinoma | N.A. | ≤ 3cm | No |
| IP3 | 35 | invasive ductal carcinoma | N.A. | ≤ 3cm | No |
| IP4 | 36 | invasive ductal carcinoma | N.A. | ≤ 3cm | No |
| IP5 | 36 | invasive ductal carcinoma | N.A. | ≤ 3cm | No |
| IP6 | 32 | invasive ductal carcinoma | N.A. | ≤ 3cm | No |

N.A.: Not application.

Table S5. The treatment information of 16 patients with GLM who were infected with *C. kroppenstedtii*

| Patient ID | Age | Surgery/puncture date | Culture result | NTS result | Drug of irrigation | Drug therapy | Discharge date | Relapse date |
| --- | --- | --- | --- | --- | --- | --- | --- | --- |
| GP2 | 40 | 2017/6/28 | Negtive | *C. kroppenstedtii* | iodophor；metronidazole；gendamycin | prednisone | 2017/7/4 | No |
| GP30 | 29 | 2017/7/12 | Negtive | *C. kroppenstedtii* | Iodophor; metronidazole | prednisone | 2017/7/23 | 2019/6/17 |
| GP36 | 33 | 2017/7/4 | *C. kroppenstedtii* | N.A. | Iodophor; metronidazole | prednisone | 2017/7/11 | No |
| GP12 | 26 | 2017/8/25 | *C. kroppenstedtii* | N.A. | Iodophor; metronidazole; dexamethasone | prednisone | 2017/9/1 | 2019 |
| GP29* | 35 | 2017/10/31 | Negtive | N.A. | Metronidazole; dexamethasone; iodophor | prednisone | 2017/11/7 | 2019 |
| GP86 | 30 | 2018/7/24 | *C. kroppenstedtii* | N.A. | Iodophor; metronidazole; dexamethasone | prednisone；rifampicin | 2018/7/28 | No |
| GP87 | 34 | 2018/7/17 | *C. kroppenstedtii* | N.A. | none | none | 2018/7/22 | No |
| GP88* | 26 | 2018/2/28 | Negtive | N.A. | Iodophor; metronidazole; dexamethasone | prednisone | 2018/3/6 | 2019/8 |
| GP89 | 33 | 2018/12/18 | Negtive | *C. kroppenstedtii; Achromobacter; Cupriavidus metallidurans* | Iodophor; metronidazole; dexamethasone | prednisone；rifampicin | 2018/12/21 | No |
| GP90 | 31 | 2019/1/23 | Negtive | *C. kroppenstedtii* | none | prednisone；rifampicin | 2019/1/28 | No |
| GP91 | 30 | 2019/1/28 | Negtive | *C. kroppenstedtii* | Iodophor; metronidazole; dexamethasone | prednisone；rifampicin | 2019/1/31 | No |
| GP92 | 35 | 2019/2/12 | Negtive | *C. kroppenstedtii* | Iodophor; metronidazole; dexamethasone | prednisone；rifampicin | 2019/2/17 | No |
| GP93 | 36 | 2019/2/15 | Negtive | *C. kroppenstedtii；Acinetobacter ohnsonii；Brevibacterium casei* | none | prednisone；rifampicin | 2019/2/21 | No |
| GP94 | 48 | 2019/4/10 | N.A. | *C. kroppenstedtii* | Iodophor; metronidazole; dexamethasone | prednisone；rifampicin | 2019/4/17 | No |
| GP95 | 36 | 2019/4/18 | *C. kroppenstedtii* | *C. kroppenstedtii* | Iodophor; metronidazole; dexamethasone | prednisone；rifampicin | 2019/4/23 | No |
| GP96 | 44 | 2019/5/21 | Negtive | *Bifidobacterium breve; C. kroppenstedtii* | Iodophor; metronidazole; dexamethasone | prednisone；rifampicin | 2019/5/25 | No |

Cultures result and NTS result were from the initial admission.

*Patient GP29 and GP88 were detected as *C. kroppenstedtii*-infected by NTS at the relapse time.

N.A., not perform that test.

Table S6. The serum iron levels of patients with GLM who has a confirmed diagnosis of *C. kroppenstedtii*.

| Patient ID | Age | Whether refractory GLM | *C. kroppenstedtii* | Primary serum iron concentration (µmol/L) * |
| --- | --- | --- | --- | --- |
| GP76 | 28 | Yes | Positive | 7.3 |
| GP77 | 31 | No | Positive | 12.8 |
| GP78 | 35 | No | Positive | 18.1 |
| GP79 | 30 | No | Positive | 7.5 |
| GP80 | 33 | Yes | Positive | 2.4 |
| GP81 | 33 | No | Positive | 22.5 |
| GP82 | 38 | No | Positive | 5.4 |
| GP83 | 29 | No | Positive | 6.6 |
| GP84 | 32 | Yes | Positive | 7.7 |
| GP85 | 32 | Yes | Positive | 2.2 |

*Normal range of concentration of serum iron: 9-30.4 μmol/L.

Table S7. Inhibitory effect of compounds on bacteria by agar diffusion assay.

|  | 1 (0.01 µmol) | 2 (0.01 µmol) |
| --- | --- | --- |
| *Lactococcus lactis* J1-004 | No | Yes |
| *L. lactis* MG1363 | No | Yes |
| *Lactobacillus reuteri* DSM 17938 | No | No |
| *C. glutamicum* ATCC 13032 | No | Yes |
| *Escherichia coli* DH10b | No | No |

Table S8. The information of *C. kroppenstedtii*-related genomes downloaded from NCBI

| Microorganism Name | Strain | Accession | Assembly | Source | Size(Mb) | GC% | Reference |
| --- | --- | --- | --- | --- | --- | --- | --- |
| *C. parakroppenstedtii* | MC-19 | JAKJKW010000001 | GCA_021713475.1 | Patient with matitis | 2.58996 | 56.7 | 2 |
| *C. parakroppenstedtii* | MC-11 | JAFFSY010000001 | GCA_019754905.1 | Patient with matitis | 2.5838 | 56.9 | 2 |
| *C. parakroppenstedtii* | MC-20 | JAKJKP010000001 | GCA_021713555.1 | Patient with matitis | 2.56153 | 56.8 | 2 |
| *C. parakroppenstedtii* | MC-09 | JAKKNX010000001 | GCA_021742085.1 | Patient with matitis | 2.96289 | 57.2 | 2 |
| *C. parakroppenstedtii* | MC-13 | JAKKFA010000001 | GCA_021726435.1 | Patient with matitis | 2.72548 | 55.7 | 2 |
| *C. parakroppenstedtii* | MC-21 | JAKJKQ010000001 | GCA_021713155.1 | Patient with matitis | 2.58426 | 56.8 | 2 |
| *C. parakroppenstedtii* | MC-23 | JAKJKS010000001 | GCA_021713185.1 | Patient with matitis | 2.58521 | 56.8 | 2 |
| *C. parakroppenstedtii* | MC-27 | JAKJKT010000001 | GCA_021713175.1 | Patient with matitis | 2.55742 | 56.8 | 2 |
| *C. parakroppenstedtii* | MC-25 | JAKKOA010000001 | GCA_021738765.1 | Patient with matitis | 2.63102 | 56.5 | 2 |
| *C. parakroppenstedtii* | MC-12 | JAKJKU010000001 | GCA_021713255.1 | Patient with matitis | 2.63254 | 56.3 | 2 |
| *C. parakroppenstedtii* | MC-08 | JAKLTI010000001 | GCA_022012535.1 | Patient with matitis | 2.54254 | 56.8 | 2 |
| *C. parakroppenstedtii* | MC-22 | JAKJKR010000001 | GCA_021713235.1 | Patient with matitis | 2.54679 | 56.8 | 2 |
| *C. parakroppenstedtii* | MC-16 | JAKJKV010000001 | GCA_021713365.1 | Patient with matitis | 2.59495 | 56.6 | 2 |
| *C. parakroppenstedtii* | MC-04 | JAKJKY010000001 | GCA_021713495.1 | Patient with matitis | 2.55753 | 56.8 | 2 |
| *C. parakroppenstedtii* | MC-01 | JAKJKX010000001 | GCA_021713535.1 | Patient with matitis | 2.53546 | 56.8 | 2 |
| *C. parakroppenstedtii* | MC-29 | JAKJKO010000001 | GCA_021713135.1 | Patient with matitis | 2.53173 | 56.8 | 2 |
| *C. parakroppenstedtii* | MC-24 | JAGSNZ010000001 | GCA_019754975.1 | Patient with matitis | 2.52726 | 56.8 | 2 |
| *C. parakroppenstedtii* | MC-28 | JAGSNY010000001 | GCA_019754895.1 | Patient with matitis | 2.50139 | 56.9 | 2 |
| *C. parakroppenstedtii* | MC-15 | JAKLTJ010000001 | GCA_022012515.1 | Patient with matitis | 2.5602 | 56.8 | 2 |
| *C. parakroppenstedtii* | MC-26 | JAGSOA010000001 | GCA_019754935.1 | Patient with matitis | 2.51553 | 56.9 | 2 |
| *C. parakroppenstedtii* | MC-06 | JAKJLA010000001 | GCA_021713215.1 | Patient with matitis | 2.59334 | 56.7 | 2 |
| *C. parakroppenstedtii* | MC-05 | JAKJKZ000000001 | GCA_021713655.1 | Patient with matitis | 2.59436 | 56.8 | 2 |
| *C. parakroppenstedtii* | MC-10 | JAKKNY010000001 | GCA_021738785.1 | Patient with matitis | 2.73365 | 55.7 | 2 |
| *C. pseudokroppenstedtii* | MC-07 | JAKJLB010000001 | GCA_021713515.1 | Patient with matitis | 2.49094 | 57.2 | 2 |
| *C. pseudokroppenstedtii* | MC-02 | JAKKNZ010000001 | GCA_021738745.1 | Patient with matitis | 2.67249 | 56.1 | 2 |
| *C. pseudokroppenstedtii* | MC-03 | JAKLTK010000001 | GCA_022012575.1 | Patient with matitis | 2.51569 | 57.2 | 2 |
| *C. pseudokroppenstedtii* | MC-17X | JAEUWU010000001 | GCA_019754945.1 | Patient with matitis | 2.47381 | 57.2 | 2 |
| *C. kroppenstedtii* | UMB0869 | NZ_PNHH00000000 | GCA_002871755.1 | female urinary microbiota | 2.51626 | 56.9 | - |
| *C. kroppenstedtii* | tJKWVvX4Fs_bin.3.MAG | CAMIAO000000000 | GCA_946222665.1 | human skin metagenome | 2.4444 | 56.8 | - |
| *C. kroppenstedtii* | S2_003_000_R1_3 | QFRA00000000 | GCA_003243515.1 | NICU indoor metagenome | 2.50701 | 56.8 | 3 |
| *C. kroppenstedtii* | 217 | DYYZ00000000 | GCA_020741425.1 | chicken gut microbiome | 2.08325 | 57.7 | 4 |
| *C. kroppenstedtii* | DSM 44385 | NC_012704 | GCA_000023145.1 | human sputum | 2.4468 | 57.5 | 5 |
| *C. kroppenstedtii* | FDAARGOS_1192 | NZ_CP069509 | GCA_016889365.1 | -- | 2.44681 | 57.5 | - |
| *C. kroppenstedtii* | yu01 | CP104321 | GCA_025252365.1 | mastitis | 2.53682 | 56.8 | - |
| *C. kroppenstedtii* | FDAARGOS_1194 | NZ_CP069512 | GCA_016889405.1 | -- | 2.51188 | 57.1 | - |
| *C. kroppenstedtii* | FDAARGOS_1193 | NZ_CP069792 | GCA_016894245.1 | -- | 2.48371 | 56.1 | - |
| *C. kroppenstedtii* | ITA205 | NZ_JYDD00000000 | GCA_000971855.1 | Patient with GLM | 2.51438 | 56.9 | 6 |
| *C. kroppenstedtii* | ITA105 | NZ_JYCR00000000 | GCA_000971845.1 | Patient with GLM | 2.57461 | 56.8 | 6 |
| *C. kroppenstedtii* | DNF00591 | NZ_LSCY00000000 | GCA_001552955.1 | -- | 2.53574 | 56.8 | - |

Table S9. ^1^H and ^13^C NMR Spectroscopic Data of 1 and 2 (600 and 150 MHz, respectively, in CD_3_OD)

| position^a^ | **1** | | **2** | |
| --- | --- | --- | --- | --- |
|  | *δ*_H_ | *δ*_C_ | *δ*_H_ | *δ*_C_ |
| Glu′-1 | 5.05, d (3.8) | 95.6 | 5.22, d (3.8) | 92.8 |
| 2 | 3.47, d (9.8, 3.8) | 73.0 | 4.74, dd (10.1, 3.8) | 74.3 |
| 3 | 3.72, t (9.4) | 74.8 | 3.93, t (9.6) | 72.1 |
| 4 | 3.31, t (9.2) | 71.8 | 3.42, t (9.4) | 71.7 |
| 5 | 3.63, ddd (9.0, 5.4, 2.4) | 74.3 | 3.70, ddd (9.2, 5.2, 2.1) | 74.4 |
| 6 | 3.66, dd (12.4, 5.4)  3.77, dd (12.4, 2.4) | 62.5 | 3.80, dd (12.0, 5.1)  3.68, dd (12.0, 2.0) | 62.3 |
| Glu″-1 | 5.26, d (3.7) | 92.6 | 5.25, d (3.8) | 92.6 |
| 2 | 4.90, dd (10.0, 3.7) | 73.4 | 4.92, dd (10.0, 3.8) | 73.0 |
| 3 | 4.15, t (10.0) | 81.8 | 3.98, t (9.4) | 82.0 |
| 4 | 3.53, dd (10.1, 9.0) | 70.3 | 3.47, t (9.3) | 70.7 |
| 5 | 4.14, ddd (9.0, 5.1, 2.1) | 71.2 | 3.89, ddd (10.0, 7.2, 2.0) | 71.5 |
| 6 | 4.23, dd (12.0, 5.1)  4.41, dd (12.0, 2.1) | 64.1 | 4.20, dd (11.8, 7.2)  4.38, dd (11.8, 2.0) | 64.6 |
| Glu‴-1 | 4.44, d (7.8) | 105.3 | 4.46, d (7.8) | 105.3 |
| 2 | 3.20, dd (9.2, 7.8) | 74.9 | 3.20, dd (9.3, 7.8) | 74.9 |
| 3 | 3.38, t (9.0) | 77.8 | 3.38, t (9.3) | 77.8 |
| 4 | 3.30, t (9.3) | 71.4 | 3.30, t (9.4) | 71.4 |
| 5 | 3.33, ddd (9.6, 5.6, 2.4) | 78.0 | 3.35, ddd (9.3, 5.9, 2.2) | 78.0 |
| 6 | 3.67, dd (12.3, 5.6)  3.88, dd (12.3, 2.4) | 62.5 | 3.68, dd (12.0, 5.9)  3.90, dd (12.0, 2.2) | 62.5 |
| Agly′-1 |  | 173.8 |  | 173.8 |
| 2 | 2.69, m  2.74, dt (9.9, 6.9) | 30.2 | 2.70, m  2.75, m | 30.2 |
| 3 | 2.61, t (6.7) | 29.9 | 2.61, m | 30.0 |
| 4 |  | 176.8 |  | 176.8 |
| Agly″-1 |  | 175.2 |  | 175.1 |
| 2 | 2.35, t (7.4) | 34.4 | 2.34, t (7.4) | 34.9 |
| 3 | 1.67, p (7.4) | 26.0 | 1.60, m | 26.0 |
| 4 | 2.09, q (7.2) | 27.5 | 1.28-1.36, m | 30.1-30.8 |
| 5 | 5.34, dt (8.4, 7.2) | 129.6 | 1.28-1.36, m | 30.1-30.8 |
| 6 | 5.40, dt (8.4, 7.0) | 132.1 | 1.28-1.36, m | 30.1-30.8 |
| 7 | 2.03, q (7.0) | 28.2 | 1.28-1.36, m | 30.1-30.8 |
| 8 | 1.28-1.36, m | 30.1-30.8 | 2.09, q (7.3) | 28.2 |
| 9 | 1.28-1.36, m | 30.1-30.8 | 5.37, dt (8.2, 7.3) | 129.2 |
| 10 | 1.28-1.36, m | 30.1-30.8 | 5.34, dt (8.2, 7.2) | 131.1 |
| 11 | 1.28-1.36, m | 30.1-30.8 | 2.81, m | 26.4 |
| 12 | 1.27, m | 33.1 | 1.28-1.36, m | 30.1-30.8 |
| 13 | 1.31, m | 23.7 | 1.28-1.36, m | 30.1-30.8 |
| 14 | 0.90, t (6.9) | 14.5 | 1.28-1.36, m | 30.1-30.8 |
| 15 |  |  | 1.28-1.36, m | 30.1-30.8 |
| 16 |  |  | 1.27, m | 33.1 |
| 17 |  |  | 1.31, m | 23.7 |
| 18 |  |  | 0.90, t (7.0) | 14.5 |
| Agly‴-1 |  |  |  | 173.0 |
| 2 |  |  | 2.54, m | 43.2 |
| 3 |  |  | 4.02, m | 69.2 |
| 4 |  |  | 1.50, m | 38.2 |
| 5 |  |  | 1.38, m | 26.5 |
| 6 |  |  | 1.28-1.36, m | 30.1-30.8 |
| 7 |  |  | 1.28-1.36, m | 30.1-30.8 |
| 8 |  |  | 1.28-1.36, m | 30.1-30.8 |
| 9 |  |  | 1.31, m | 23.7 |
| 10 |  |  | 0.90, t (7.0) | 14.5 |

^a^Abbreviations: Glu = glucose, Agly = aglycone.

Reference

1. Roslund MU, Tahtinen P, Niemitz M, Sjoholm R. Complete assignments of the (1)H and (13)C chemical shifts and J(H,H) coupling constants in NMR spectra of D-glucopyranose and all D-glucopyranosyl-D-glucopyranosides. *Carbohydr. Res.* 343, 101-112 (2008).

2. Luo Q, et al. Classification of 27 *Corynebacterium kroppenstedtii*-like isolates associated with mastitis in China and descriptions of *C. parakroppenstedtii* sp. nov. and *C. pseudokroppenstedtii* sp. nov. *Microbiol. Spectr.* 10, e0137221 (2022).

3. Brooks B, et al. Strain-resolved analysis of hospital rooms and infants reveals overlap between the human and room microbiome. *Na.t Commun.* 8, 1814 (2017).

4. Gilroy R, et al. Extensive microbial diversity within the chicken gut microbiome revealed by metagenomics and culture. *PeerJ* 9, e10941 (2021).

5. Tauch A, et al. Ultrafast pyrosequencing of *Corynebacterium kroppenstedtii* DSM44385 revealed insights into the physiology of a lipophilic *Corynebacterium* that lacks mycolic acids. *J. Biotechnol.* 136, 22-30 (2008).

6. Fernandez-Natal MI, et al. Draft genome sequences of *Corynebacterium kroppenstedtii* CNM633/14 and CNM632/14, multidrug-resistant and antibiotic-sensitive isolates from nodules of granulomatous mastitis patients. *Genome Announc.* 3, e00525-15 (2015).
