# Supplementary material for: Corynebacterium parakroppenstedtii secretes a novel glycolipid to promote the development of granulomatous lobular mastitis
Source: Signal Transduct Target Ther. 2024 Oct 21;9:292. doi: 10.1038/s41392-024-01984-0 (PMC11491465; doi:10.1038/s41392-024-01984-0)
Supplement: Supplementary file 2 — Change of authorship request form [file 41392_2024_1984_MOESM2_ESM.pdf]

SPRINGER NATURE

Change of authorship request form - Journals (pre-acceptance)

Important information. Please read.

- This form should be used by authors to request any change in authorship (adding/deleting authors) including changes in corresponding authors. This form should not be used for name changes. Please fully complete all sections. Use black ink and block capitals and provide each author's full name with the given name first followed by the family name.

- By signing this declaration, all authors guarantee that the order of the authors are in accordance with their scientific contribution, if applicable as different conventions apply per discipline, and that only authors have been added who made a meaningful contribution to the work.

- Please note, in author collaborations where there is formal agreement for representing the collaboration, it is sufficient for the representative or legal guarantor (usually the corresponding author) to complete and sign the Authorship Change Form on behalf of all authors, **next to the added/removed author(s)**. **(Complete Section 3, followed by Section 6.)**
  In author collaborations where there is no formal agreement for representing the collaboration and **there are more than 10 authors**, one may sign for all, provided the signer appends correspondence that attests that each of the authors have agreed to the change **and the added/removed authors sign the form**. **(Complete Section 3, followed by Section 6.)**

- Please note, we cannot investigate or mediate any authorship disputes. If you are unable to obtain agreement from all authors (including those who you wish to be removed) you must refer the matter to your institution(s) for investigation. Please inform us if you need to do this.

- If you are not able to return a fully completed form within **30 days** of the date that it was sent to the author requesting the change, we may have to withdraw your manuscript. We cannot publish manuscripts where authorship has not been agreed by all authors (including those who have been removed).

- Incomplete forms will be rejected.

- Please return/upload this form, fully completed, to the Journals Editorial Office. The Journal and/or Publisher will consider the information you have provided to decide whether to approve the proposed change in authorship. We may decide to contact your institution for more information or undertake a further investigation, if appropriate, before making a final decision.

SPRINGER NATURE Springer Nature is one of the world's leading global research, educational and professional publishers, created in May 2015 through the combination of Nature Publishing Group, Palgrave Macmillan, Macmillan Education and Springer Science+Business Media.

SPRINGER NATURE

Change of authorship request form - Journals (pre-acceptance)

Section 1: Please provide the current title of manuscript

Manuscript ID no.: SIGTRANS-13328R1

Title: *Corynebacterium parakroppenstedtii* secretes a novel glycolipid to promote the development of granulomatous lobular mastitis

Section 2: Please provide the previous authorship, in the order shown on the manuscript before the changes were introduced. Please indicate the corresponding author by adding (CA) behind the name.

	First name(s)	Family name	ORCID or SCOPUS id, if available
1st author	Ran	Liu	ORCID: 0000-0002-9605-231X
2nd author	Zixuan	Luo	ORCID: 0009-0007-2891-4735
3rd author	Chong	Dai	
4th author	Yuchen	Wei	
5th author	Xinwen	Kuang	ORCID: 0000-0002-6876-7844
6th author	Aisi	Fu	
7th author	Yinxin	Li	
8th author	Shuai	Fu	
9th author	Zhengning	Ma	
10th author	Wen	Dai	

Please use an additional sheet if there are more than 10 authors.

SPRINGER NATURE Springer Nature is one of the world's leading global research, educational and professional publishers, created in May 2015 through the combination of Nature Publishing Group, Palgrave Macmillan, Macmillan Education and Springer Science+Business Media.

SPRINGER NATURE

Change of authorship request form - Journals (pre-acceptance)

	First name(s)	Family name	ORCID or SCOPUS id, if available
11th author	Xiao	Xiao	
12th author	Qing	Wu	
13th author	HaoKui	Zhou	ORCID: 0000-0002-6931-3377
14th author	Yan	Rao	ORCID: 0000-0001-7247-4342
15th author	Jingping	Yuan	ORCID: 0000-0001-7470-6899
16th author	Zixin	Deng (CA)	
17th author	Chuang	Chen (CA)	
18th author	Tiangang	Liu (CA)	ORCID: 0000-0001-8087-0345

SPRINGER NATURE Springer Nature is one of the world's leading global research, educational and professional publishers, created in May 2015 through the combination of Nature Publishing Group, Palgrave Macmillan, Macmillan Education and Springer Science+Business Media.

Change of authorship request form - Journals (pre-acceptance)

Section 3: Please provide a justification for change. Please use this section to explain your reasons for changing the authorship of your manuscript, e.g. what necessitated the change in authorship? Please refer to the (journal) policy pages for more information about authorship. Please explain why omitted authors were not originally included and/or why authors were removed on the submitted manuscript.

Shuqing Yan, Kuan Qi, and Ting Shi were added into the authorship in the revised manuscript. Because in the process of revise, Shuqing Yan and Kuan Qi performed lots of experiments, including culturing *C. parakroppenstedtii*, measuring the growth curves of *C. parakroppenstedtii*, isolating the corynekropbactins, and measuring the characteristics of the corynekropbactins and its Fe-complex. Ting Shi helped to analyse and judge the ability of corynekropbactins to chelate iron.

Section 4: Proposed new authorship. Please provide your new authorship list in the order you would like it to appear on the manuscript. Please indicate the corresponding author by adding (CA) behind the name. <u>If the Corresponding Author has changed, please indicate the reason under section 3</u>.

	First name(s)	Family name (this name will appear in full on the final publication and will be searchable in various abstract and indexing databases)	Affiliated institute	E-mail address
1st author	Ran	Liu	Renmin Hospital of Wuhan University	l_ran@sjtu.edu.cn
2nd author	Zixuan	Luo	Renmin Hospital of Wuhan University	luozixuan@whu.edu.cn
3rd author	Chong	Dai	Wuhan University	daichong@whu.edu.cn
4th author	Yuchen	Wei	The Chinese University of Hong Kong	yuchenwei@cuhk.edu.hk
5th author	Shuqing	Yan	Wuhan University	yanshuqing1120@163.com
6th author	Xinwen	Kuang	Renmin Hospital of Wuhan University	abbiekuang17@foxmail.com
7th author	Kuan	Qi	Zhongnan Hospital of Wuhan University	qikuan@whu.edu.cn
8th author	Aisi	Fu	Dgensee Co., Ltd	fuaisi@dgensee.com
9th author	Yinxin	Li	Renmin Hospital of Wuhan University	liyinxin1997@163.com
10th author	Shuai	Fu	Hesheng Tech, Co., Ltd	fushuai@heshengtech.com.cn

Please use an additional sheet if there are more than 10 authors.

SPRINGER NATURE Springer Nature is one of the world's leading global research, educational and professional publishers, created in May 2015 through the combination of Nature Publishing Group, Palgrave Macmillan, Macmillan Education and Springer Science+Business Media.

SPRINGER NATURE

Change of authorship request form - Journals (pre-acceptance)

	First name(s)	Family name (this name will appear in full on the final publication and will be searchable in various abstract and indexing databases)	Affiliated institute	E-mail address
11th author	Zhengning	Ma	Wuhan University	zhengningma@whu.edu.cn
12th author	Wen	Dai	Renmin Hospital of Wuhan University	nickissy@163.com
13th author	Xiao	Xiao	Zhongnan Hospital of Wuhan University	xiaox1017@whu.edu.cn
14th author	Qing	Wu	Renmin Hospital of Wuhan University	wuqing198502@163.com
15th author	Haokui	Zhou	Shenzhen Institute of Advanced Technology, CAS	hk.zhou@siat.ac.cn
16th author	Yan	Rao	Wuhan University	RAO@whu.edu.cn
17th author	Jingping	Yuan	Renmin Hospital of Wuhan University	yuanjingping@whu.edu.cn
18th author	Ting	Shi	Shanghai Jiao Tong University	tshi@sjtu.edu.cn
19th author	Zixin	Deng (CA)	Shanghai Jiao Tong University	zxdeng@sjtu.edu.cn
20th author	Chuang	Chen (CA)	Renmin Hospital of Wuhan University	chenc2469@whu.edu.cn
21st author	Tiangang	Liu (CA)	Shanghai Jiao Tong University	liutg@whu.edu.cn

---
SPRINGER NATURE Springer Nature is one of the world's leading global research, educational and professional publishers, created in May 2015 through the combination of Nature Publishing Group, Palgrave Macmillan, Macmillan Education and Springer Science+Business Media.

SPRINGER NATURE

Change of authorship request form - Journals (pre-acceptance)

Section 5: Author contribution, Acknowledgement and Disclosures. Please use this section to provide a new disclosure statement and, if appropriate, acknowledge any contributors who have been removed as authors and ensure you state what contribution any new authors made (if applicable per the journal or book (series) policy). Please ensure these are updated in your manuscript - after approval of the change(s) - as our production department will not transfer the information in this form to your manuscript.

New acknowledgements:

We thank for Prof. Wenjun Li and Dr. Lan Liu in Sun Yat-sen University for the guidance on strain classification.

New Disclosures (financial and non-financial interests, funding):

This work was supported by the National Key Research and Development Program of China (2018YFA0900400 to T. L.), Leading Talents of the Ten Thousand Talents Program to T. L., Young Elite Scientists Sponsorship Program by China Association for Science and Technology to R. L., the Shenzhen Institute of Synthetic Biology Scientific Research Program (Z. X. and R. L.), funding from State Key Laboratory of Microbial Metabolism, Shanghai Jiao Tong University (MMLZD24-01), the grants from The Interdisciplinary Innovative Talents Foundation from Renmin Hospital of Wuhan University (JCRCFZ-2022-015) to C.C., the Fundamental Research Funds for the Central Universities (2042019kf0229) to C.C., and the Natural Science Foundation of Hubei Province (2023AFB701) to C.C.

New Author Contributions statement (if applicable per the journal policy):

R. L., Z. D., C. C., and T. L. designed the overall study. R. L. performed experiments (bacterial culture and detection, cell experiment), analyzed all data, wrote and revised the manuscript. Z.L., X. K., Y. L, and Y. R. performed animal experiments. C. D., S. Y., and K. Q. isolated corynekropbactins and performed chelate metal ions assay. C. D. identified the structure of corynekropbactins. Z. M. performed antibacterial assay. A. F. designed sequencing experiment. Y. W. performed bioinformatics analysis and statistical analysis. S. F. and C. D. performed HPLC and LC-MS analysis. W. D. performed cytokines and iron-related factors testing. Q. W. and X. X. isolated microorganisms from clinical samples. H. Z. designed the analysis of microbiome data. J. Y. analyzed pathological sections. T. S. did the simulate calculate. Z. D, C. C., and T. L. supervised the overall study, C. C., and T. L. revised the manuscript. All authors have read and approved the article.

State 'Not applicable' if there are no new authors.

SPRINGER NATURE Springer Nature is one of the world's leading global research, educational and professional publishers, created in May 2015 through the combination of Nature Publishing Group, Palgrave Macmillan, Macmillan Education and Springer Science+Business Media.

Change of authorship request form - Journals (pre-acceptance)

Section 6: Declaration of agreement. *All* authors, unchanged, new and removed *must* sign this declaration.
(NB: Please print the form, (docu)-sign and return/upload a scanned copy. Please note that signatures that have been inserted as an image file are acceptable as long as it is handwritten. Typed names in the signature box are unacceptable.) *** Please delete as appropriate. Delete all of the bold if you were on the original authorship list and are remaining as an author.**

	First name	Family name		Signature	Date
1st author	Ran	Liu	I agree to the proposed new authorship shown in section 4	Ran Liu	2024.9.10
2nd author	Zixuan	Luo	I agree to the proposed new authorship shown in section 4	Zixuan Luo	2024.9.10
3rd author	Chong	Dai	I agree to the proposed new authorship shown in section 4	Chong Dai	2024.9.10
4th authors	Yuchen	Wei	I agree to the proposed new authorship shown in section 4	WEI, Yuchen	2024.9.10
5th author	Shuqing	Yan	I agree to the proposed new authorship shown in section 4 **and the addition of my name to the authorship list**	Shuqing Yan	2024.9.10
6th author	Xinwen	Kuang	I agree to the proposed new authorship shown in section 4	Xin wen Kuang	2024.9.10
7th author	Kuan	Qi	I agree to the proposed new authorship shown in section 4 **and the addition of my name to the authorship list**	Qi Kuan	2024.9.10

SPRINGER NATURE Springer Nature is one of the world's leading global research, educational and professional publishers, created in May 2015 through the combination of Nature Publishing Group, Palgrave Macmillan, Macmillan Education and Springer Science+Business Media.

Change of authorship request form - Journals (pre-acceptance)

	First name	Family name		Signature	Date
8th author	Aisi	Fu	I agree to the proposed new authorship shown in section 4	Ai Si Fu	2024.9.10
9th author	Yinxin	Li	I agree to the proposed new authorship shown in section 4	Yinxin Li	2024.9.10
10th author	Shuai	Fu	I agree to the proposed new authorship shown in section 4	Shuai Fu	2024.9.10

Please use an additional sheet if there are more than 10 authors.

SPRINGER NATURE Springer Nature is one of the world's leading global research, educational and professional publishers, created in May 2015 through the combination of Nature Publishing Group, Palgrave Macmillan, Macmillan Education and Springer Science+Business Media.

SPRINGER NATURE

Change of authorship request form - Journals (pre-acceptance)

	First name	Family name		Signature	Date
11th author	Zhengning	Ma	I agree to the proposed new authorship shown in section 4	Zhengning Ma	2024.9.10
12th author	Wen	Dai	I agree to the proposed new authorship shown in section 4	Dai Wen	2024.9.10
13th author	Xiao	Xiao	I agree to the proposed new authorship shown in section 4	Xiao Xiao	2024.9.10
14th authors	Qing	Wu	I agree to the proposed new authorship shown in section 4	Qing Wu	2024.9.10
15th author	Haokui	Zhou	I agree to the proposed new authorship shown in section 4	Haokui Zhou	2024.9.10
16th author	Yan	Rao	I agree to the proposed new authorship shown in section 4	Yan Rao	2024.9.10
17th author	Jingping	Yuan	I agree to the proposed new authorship shown in section 4	Jingping Yuan	2024.9.10

SPRINGER NATURE Springer Nature is one of the world's leading global research, educational and professional publishers, created in May 2015 through the combination of Nature Publishing Group, Palgrave Macmillan, Macmillan Education and Springer Science+Business Media.

Change of authorship request form - Journals (pre-acceptance)

	First name	Family name		Signature	Date
18th author	Ting	Shi	I agree to the proposed new authorship shown in section 4 **and the addition of my name to the authorship list**	Ting Shi	2024.9.10
19th author	Zixin	Deng	I agree to the proposed new authorship shown in section 4	Zixin Deng	2024.9.10
20th author	Chuang	Chen	I agree to the proposed new authorship shown in section 4	Chuang Chen	2024.9.10
21th author	Tiangang	Liu	I agree to the proposed new authorship shown in section 4	Tiangang Liu	2024.9.10

---- End of form ----

SPRINGER NATURE Springer Nature is one of the world's leading global research, educational and professional publishers, created in May 2015 through the combination of Nature Publishing Group, Palgrave Macmillan, Macmillan Education and Springer Science+Business Media.
